# Eutypellaolides A–J, Sesquiterpene diversity expansion of the polar fungus *Eutypella* sp. D-1

**DOI:** 10.3389/fmicb.2024.1349151

**Published:** 2024-01-25

**Authors:** Zhe Ning, Bo Hu, Yuan-Yuan Sun, Jin-Feng Ding, Xiang-Ying Han, Xiao-Ling Lu, Zi-Fei Yin, Ying He, Bing-Hua Jiao, Hao-Bing Yu, Xiao-Yu Liu

**Affiliations:** ^1^Department of Marine Biomedicine and Polar Medicine, Naval Medical Center of PLA, Naval Medical University, Shanghai, China; ^2^Department of Biochemistry and Molecular Biology, College of Basic Medical Sciences, Naval Medical University, Shanghai, China; ^3^School of Traditional Chinese Medicine, Naval Medical University, Shanghai, China

**Keywords:** sesquiterpene, polar fungus, *Eutypella* sp., OSMAC approach, PTP1B inhibition activity

## Abstract

Eight new 12,8-eudesmanolide sesquiterpenes, eutypellaolides A–H (**1**–**8**), and two new eudesmane-type sesquiterpenes, eutypellaolides I–J (**9**–**10**), along with four known 12,8-eudesmanolide compounds **11**–**14**, were isolated from the culture extract of the polar fungus *Eutypella* sp. D-1 by one strain many compounds (OSMAC) approach. The structures of these compounds were determined through comprehensive spectroscopic data and experimental and calculated ECD analysis. Antibacterial, immunosuppressive, and PTP1B inhibition activities of these compounds were evaluated. Compounds **1** and **11** exhibited strong inhibitory activities against *Bacillus subtilis* and *Staphylococcus aureus*, with each showing an MIC value of 2 μg/mL. Compound **9** displayed weak immunosuppressive activity against ConA-induced T-cell proliferation with an inhibitory rate of 61.7% at a concentration of 19.8 μM. Compounds **5**, **11**, and **14** exhibited weak PTP1B inhibition activities with IC_50_ values of 44.8, 43.2, and 49.5 μM, respectively.

## 1 Introduction

The polar regions are renowned for their harsh environmental conditions, characterized by extremely low temperatures, hurricanes, and intense ultraviolet radiation. These extreme conditions contribute to the development of unique physiological adaptations in microorganisms that inhabit the polar regions, leading to a diversity of microbial secondary metabolites (Santiago et al., 2015). Due to the exceptional conditions and abundant microbiological resources, polar regions have been an increasing interest in human activities and scientific research (Lu et al., 2014; Tian et al., 2017). However, compared with the vast number of natural products reported in tropical regions, compounds from the polar regions have been relatively limited, with only over one hundred new compounds being reported in recent years (Dos Santos et al., 2021). Consequently, polar microbiology has gained recognition as a crucial source of bioactive natural products.

**Figure 1:**
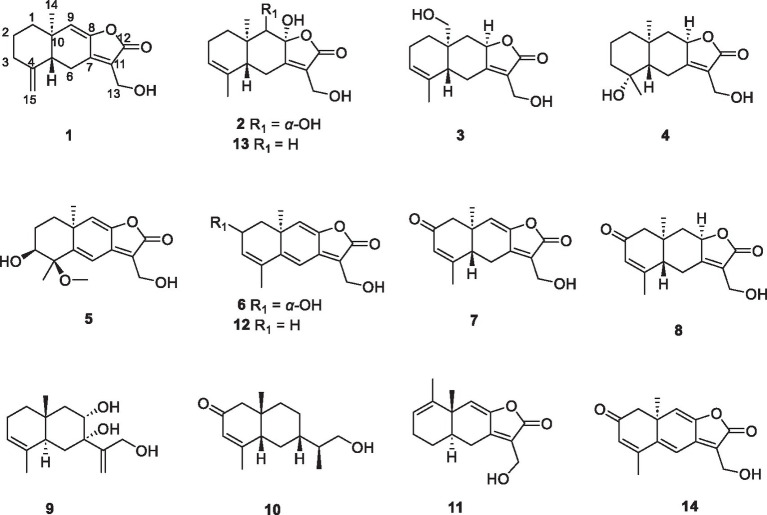
Structures of the compounds **1–14**.

The fungi of *Eutypella* genus have been extensively investigated for decades due to the respected biological and pharmacological activities of their secondary metabolites (Pongcharoen et al., 2006; Ning et al., 2023). The polar fungus *Eutypella* sp. D-1 has been discovered to have the ability to produce a variety of structurally distinct and biologically active secondary metabolites, such as cytochalasins, pimarane diterpenes, cytosporins, and sesquiterpenes, with significant antimicrobial and cytotoxic activities (Liu et al., 2014; Zhou et al., 2017; Wang et al., 2018; Yu et al., 2018a,b; Zhang et al., 2019). Previous research on *Eutypella* sp. D-1 has discovered one sesquiterpene, *eut*-Guaiane (**11**), with significant antibacterial activity (Zhou et al., 2017). The OSMAC (One Strain Many Compounds) approach has been extensively employed for the identification of novel metabolites from microorganisms. To enhance the sesquiterpene chemical diversity of *Eutypella* sp. D-1, drawing inspiration from the OSMAC strategy, we utilized different culture conditions. Subsequently, the EtOAc extracts of the different fermentation broths were subjected to HPLC analysis, and a large number of sesquiterpene analogs were found in solid defined medium compared with PDB medium (Supplementary Figure S110). A follow-up chemical investigation led to the isolation of ten new sesquiterpenes, eutypellaolides A–J (**1**–**10**), and four known related compounds **11**–**14** (Figure 1). Herein, we present the isolation, structure elucidation, and bioactive evaluation of these compounds.

## 2 Materials and methods

### 2.1 General experimental procedures

Optical rotations were obtained on the Shanghai Shenguang SGWzz-2 model automatic polarimeter (Shanghai Shenguang Instrument and Meter Co., Ltd., Shanghai, China). IR spectra of all compounds were recorded on Bruker’s VERTEX 70v FT-IR Spectrometer (Bruker Biospin Corp., Billerica, Mass., USA). UV and CD spectra were measured on a JASCO-810 model spectrometer (Jasco Inc., Tokyo, Japan). HRESIMS data were recorded on an Agilent 6,520 AccuTOF LC-plus 4G instrument (JEOL., Tokyo, Japan). The 1D and 2D spectral data were acquired on Bruker AMX-500 and Bruker AVANCE-600 instruments (Bruker Biospin Corp., Billerica, Mass., USA). Semi-performance liquid chromatography was performed on a Waters 1,525 separation module (Waters Corp., Milford, Mass., USA), with YMC-PackPro C18 RS (5 μm) columns and octadecyl silyl silica gel (50 μm, YMC Co. Ltd., Kyoto, Japan). Column chromatographic purifications were performed on silica gel 60 (200–300 mesh, Qingdao Ocean Chemical Co., Qingdao, China), ODS (50 μm, YMC Co. Ltd., Kyoto, Japan).

### 2.2 Fungal material

The strain *Eutypella* sp. D-1 was collected near the Ny-Ålesund District in the London Island of Kongsfjorden of the Arctic. It was purified at 20°C by using potato dextrose agar (PDA) medium and identified as *Eutypella* sp. through 18S rDNA gene sequence analysis (GenBank Accession number FJ430580). At present, the strain of *Eutypella* sp. D-1 was deposited at the Naval Medical University, Shanghai, China.

### 2.3 Fermentation, extraction, and isolation

The fungal strain *Eutypella* sp. D-1 was cultured on the PDB medium at 28°C for 3 days to activate the strain, and then, 5% of the activated strain was cultivated into 250 mL Erlenmeyer flasks, each containing 100 mL of seed medium (glucose 12.5%, NaNO_3_ 0.33%, MgSO_4_·7H_2_O 0.04%, K_2_HPO_4_·3H_2_O 0.007%, KCl 0.0625%, yeast extract 0.07%, Lornithine hydrochloride 1.5%, and microelement including FeSO_4_·7H_2_O 1.875‰, CoCl_2_·6H_2_O, 0.3125‰, CaCl_2_ 0.65‰). After 2 days of incubation at 28°C on a rotary shaker at 180 r/min, each containing 200 μL of activated liquid strain cultures was transferred on the modified solid defined medium (sucrose 5.14%, NaNO_3_ 0.33%, MgSO_4_·7H_2_O 0.04%, K_2_HPO_4_·3H_2_O 0.007%, KCl 0.0625%, yeast extract 0.07%, CaCl_2_ 0.65%, agar powder 2%), with total 40 pieces (φ20 × 20 cm). The modified solid cultivation was kept for 45 days at 20°C.

After subjecting the solid defined medium to 1 hour of ultrasonic treatment with a mixture of CH_2_Cl_2_/CH_3_OH (v/v, 1:1), it was subsequently extracted three times using the same mixture. The CH_2_Cl_2_/CH_3_OH solution was evaporated under reduced pressure to obtain an aqueous solution and then extracted with EtOAc three times under reduced pressure at 40°C to yield a dark brown gum (6.86 g).

The crude extract was subjected to silica gel column chromatography using petroleum ether (PE)/EtOAc in a gradient elution (v/v, 100:0, 100:1, 80:1, 50:1, 30:1, 15:1, 10:1, 5:1, 2:1, 1:1, and 0:1) to afford twenty fractions (A − T). Fr. K was purified by HPLC (65% CH_3_OH/H_2_O, 2.0 mL/min) and detected at the wavelength of 300 nm, to yield **1** (*t*_R_ = 37.4 min, 2.0 mg), **11** (*t*_R_ = 40.1 min, 234.4 mg), and **12** (*t*_R_ = 27.2 min, 147.6 mg). Fr. O was separated on an ODS (50 μm) column followed by gradient elution with MeOH/H_2_O (from 50 to 100%) to give eight subfractions (O1–O8). Fr. O3 was detected at the wavelength of 300 nm to yield **5** (*t*_R_ = 32.5 min, 3.5 mg) by further purifying using HPLC (25% CH_3_CN /H_2_O, 2.0 mL/min). Fr. O5 was isolated by HPLC with elution of 35% CH_3_CN detected at the wavelength of 240 nm to afford **13** (*t*_R_ = 33.6 min, 18.6 mg). Fr. P was separated by ODS (50 μm) using MeOH/H_2_O (from 60 to 100%) to give five subfractions (P1–P5). Compound **2** (*t*_R_ = 21.8 min, 1.5 mg) was purified from Fr. P2 by HPLC using 30% CH_3_CN/H_2_O at the wavelength of 222 nm. Compound **9** (*t*_R_ = 18.4 min, 3.0 mg) was purified from Fr. P4 by HPLC using 40% CH_3_CN/H_2_O at the wavelength of 222 nm. Compound **10** (*t*_R_ = 29.4 min, 4.1 mg) was purified from Fr. P3 by HPLC using 40% CH_3_CN/H_2_O at the wavelength of 222 nm. Fr. Q was separated by ODS (50 μm) using MeOH/H_2_O (from 60 to 100%) to give six subfractions (Q1 − Q6). Compounds **6** (*t*_R_ = 47.2 min, 9.4 mg) and **14** (*t*_R_ = 66.9 min, 19.8 mg) were isolated from Fr. Q1 by HPLC using 20% CH_3_CN/H_2_O at the wavelength of 240 nm. Fr. R was separated by ODS (50 μm) using MeOH/H_2_O (from 30 to 80%) to give five subfractions (R1–R5). Fr. R1 was separated by HPLC using 20% CH_3_CN/H_2_O to afford **7** (*t*_R_ = 33.7 min, 8.0 mg) at the wavelength of 220 nm. Fr. R2 was further separated using silica gel with a PE/EtOAc gradient elution (v/v, 10:1, 5:1, 3:1, 2:1, 1:1, and 0:1) to give five subfractions (R2a–R2e). Compounds **3** (*t*_R_ = 69.1 min, 3.3 mg) and **4** (*t*_R_ = 73.1 min, 2.9 mg) were obtained from Fr. R2d by HPLC using 37% MeOH/H_2_O at the wavelength of 220 nm. Fr. S was separated by ODS using MeOH/H_2_O (from 20 to 60%) to give three subfractions (S1–S3). Compound **8** (*t*_R_ = 32.0 min, 8.7 mg) was obtained from Fr. S1 by HPLC using 18% CH_3_CN/H_2_O at the wavelength of 245 nm.

### 2.4 Compound characterization data

Eutypellaolide A (**1**): yellowish oil; ${\left[\alpha \right]}_{D}^{25}$ −27.9 (*c* 0.1, MeOH); UV (MeOH) λ_max_ (log ε) 278 (3.98) nm; IR_νmax_ 3,444, 2,929, 2,851, 1757, 1,645, 1,260, 1,091, 1,006, 891, 795, 735 cm^−1^; ^1^H and ^13^C NMR date, table 1; HRESIMS *m/z* 269.1153 [M + Na]^+^ (calcd for C_15_H_18_O_3_ Na, 269.1148).

Eutypellaolide B (**2**): yellowish oil; ${\left[\alpha \right]}_{D}^{25}$ −15.5 (*c* 0.1, MeOH); UV (MeOH) λ_max_ (log ε) 222 (3.64) nm; IR _νmax_ 3,323, 2,924, 2,854, 1742, 1,587, 1,381, 1,204, 1,125, 1,024, 950, 880, 794, 625 cm^−1^; ^1^H and ^13^C NMR date, table 2; HRESIMS *m/z* 303.1203 [M + Na]^+^ (calcd for C_15_H_20_O_5_Na, 303.1203).

Eutypellaolide C (**3**): yellowish oil; ${\left[\alpha \right]}_{D}^{25}$ −31.9 (*c* 0.5, MeOH); UV (MeOH) λ_max_ (log ε) 218 (4.24), 276 (2.98) nm; IR _νmax_ 3,386, 2,927, 1731, 1,675, 1,600, 1,442, 1,345, 1,219, 1,069, 1,014, 956, 798, 702, 617 cm^−1^; ^1^H and ^13^C NMR data, table 3; HRESIMS *m/z* 287.1266 [M + Na]^+^ (calcd for C_15_H_20_O_4_Na, 287.1254).

Eutypellaolide D (**4**): yellowish oil; ${\left[\alpha \right]}_{D}^{25}$ −47.2 (*c* 0.1, MeOH); UV (MeOH) λ_max_ (log ε) 238 (4.16), 278 (4.12) nm; IR _νmax_ 3,420, 2,929, 2,865, 1733, 1,674, 1,600, 1,457, 1,381, 1,351, 1,220, 1,135, 1,078, 1,009, 919, 838, 696 cm^−1^; ^1^H and ^13^C NMR data, table 4; HRESIMS *m/z* 289.1401 [M + Na]^+^ (calcd for C_15_H_22_O_4_Na, 289.1410).

Eutypellaolide E (**5**): yellowish oil; ${\left[\alpha \right]}_{D}^{25}$ −44.0 (*c* 0.1, MeOH); UV (MeOH) λ_max_ (log ε) 302 (4.17) nm; IR _νmax_ 3,424, 2,930, 1748, 1,692, 1,633, 1,597, 1,450, 1,371, 1,312, 1,205, 1,162, 1,096, 1,069, 984, 867, 669 cm^−1^; ^1^H and ^13^C NMR data, table 5; HRESIMS *m/z* 315.1204[M + Na]^+^ (calcd for C_16_H_20_O_5_Na, 315.1203).

Eutypellaolide *F* (**6**): yellowish oil; ${\left[\alpha \right]}_{D}^{25}$ +125.7 (*c* 0.1, MeOH); UV (MeOH) λ_max_ (log ε) 218 (4.17), 325 (4.27) nm; IR _νmax_ 3,396, 2,922, 2,853, 1744, 1,619, 1,452, 1,329, 1,213, 1,071, 996, 958, 885, 788 cm^−1^; ^1^H and ^13^C NMR data, table 6; HRESIMS 283.0942 *m/z* [M + Na]^+^ (calcd for C_15_H_16_O_4_Na, 283.0941).

Eutypellaolide G (**7**): yellowish oil; ${\left[\alpha \right]}_{D}^{25}$ −9.3 (*c* 0.1, MeOH); UV (MeOH) λ_max_ (log ε) 220 (3.99) nm; IR _νmax_ 3,375, 2,925, 2,857, 1743, 1,686, 1,619, 1,453, 1,377, 1,330, 1,214, 1,127, 1,071, 997, 959, 886, 827, 749 cm^−1^; ^1^H and ^13^C NMR data, table 8; HRESIMS *m/z* 283.0947 [M + Na]^+^ (calcd for C_15_H_16_O_4_Na, 283.0941).

Eutypellaolide H (**8**): yellowish oil; ${\left[\alpha \right]}_{D}^{25}$ −91.2 (*c* 0.1, MeOH); UV (MeOH) λ_max_ (log ε) 225 (4.27) nm; IR _νmax_ 3,373, 2,925, 1740, 1,649, 1,434, 1,380, 1,333, 1,078, 1,013, 621 cm^−1^; ^1^H and ^13^C NMR data, table 9; HRESIMS *m/z* 285.1103 [M + Na]^+^ (calcd for C_15_H_18_O_4_Na, 285.1097).

Eutypellaolide I (**9**): white powder; ${\left[\alpha \right]}_{D}^{25}$ +0.6 (*c* 0.1, MeOH); UV (MeOH) λ_max_ (log ε) 194 (3.40) nm; IR _νmax_ 3,251, 2,915, 1,639, 1,434, 1,378, 1,272, 1,215, 1,062, 1,031, 1,021, 900, 795, 674 cm^−1^; ^1^H and ^13^C NMR data, table 10; HRESIMS *m/z* 275.1620 [M + Na]^+^ (calcd for C_15_H_24_O_3_Na, 275.1618).

Eutypellaolide J (**10**): yellowish oil; ${\left[\alpha \right]}_{D}^{25}$ −66.2 (*c* 0.1, MeOH); UV (MeOH) λ_max_ (log ε) 238 (3.90) nm; IR _νmax_ 3,425, 2,928, 2,873, 1,655, 1,617, 1,438, 1,378, 1,347, 1,294, 1,260, 1,037, 985, 905, 869, 837 cm^−1^; ^1^H and ^13^C NMR data, table 10; HRESIMS *m/z* 237.1847 [M + H]^+^ (calcd for C_15_H_25_O_2_, 237.1849).

### 2.5 Biological assay

The antimicrobial activities of compounds **1**–**14** against (*Bacillus subtilis*) (ATCC 21951), (*Staphylococcus aureus*) (ATCC 27217), (*Pseudomonas aeruginosa*) (ATCC 27853), (*Vibrio vulnificus*) (ATCC 27562), and (*Vibrio parahaemolyticus*) (ATCC 17802) were evaluated as previously described (Liao et al., 2017), with levofloxacin positive control. The immunosuppressive activities of compounds **1**–**14** against ConA-induced T-cell proliferation were performed as previously described (Xu et al., 2021), with cyclosporin A used as a positive control. The inhibitory activity of all isolates against PTP1B was tested in 96-well microplates, as previously reported (Hou et al., 2019), with oleanolic acid used as a positive control. PTP1B was purchased from Sino Biological, Inc. (Beijing, China).

## 3 Results and discussion

Eutypellaolide A (**1**) was obtained as a yellowish oil. Its molecular formula C_15_H_18_O_3_ was established through HRESIMS (*m/z* 269.1153 [M + Na]^+^), indicating seven degrees of unsaturation. The IR absorption at 3444, 1757, and 1,645 cm^−1^ confirmed the existence of the α,β-unsaturated γ-lactone carbonyl group (Jang et al., 2017). The ^1^H NMR spectrum showed exocyclic double bond signals at *δ*_H_ 4.92 (1.5) and 4.62 (1.5), one olefinic singlet at *δ*_H_ 5.78, and one methyl singlet at *δ*_H_ 0.96 (Table 1). The ^13^C NMR date for **1** revealed the presence of one ester carbonyl at *δ*_C_ 170.4 and six olefinic carbons at *δ*_C_ 150.3, 147.8, 147.6, 122.3, 122.3, and 107.8. The NMR data accounted for four degrees of unsaturation, implying a tricyclic core structure in **1** (Tables 2−4).

**Table 1:** NMR Data of compounds **1–3**.

position	1^a^		2^b^		3^a^	
	*δ*_C_	*δ*_H_, mult. (*J* in Hz)	*δ*_C_	*δ*_H_, mult. (*J* in Hz)	*δ*_C_	*δ*_H_, mult. (*J* in Hz)
1*α*	38.9, CH_2_	1.64, m	33.0, CH_2_	1.84, m	31.4, CH_2_	1.91, m
1*β*				1.07, m		1.28, m
2	23.0, CH_2_	1.72, m	22.1, CH_2_	1.97, m	22.5, CH_2_	2.10, m
3*α*	36.1, CH_2_	2.05, m	121.6, CH	5.34, s	123.2, CH	5.46, s
3*β*		2.38, m				
4	147.6, C		132.4, C		131.9, C	
5	48.4, CH	2.36, m	45.7, CH	1.89, m	46.6, CH	2.20, s
6*α*	22.7, CH_2_	2.61, dd (15.0, 2.0)	23.2, CH_2_	2.04, t (13.0)	25.8, CH_2_	2.22, s
6*β*		2.88, dd (17.0, 4.0)		3.12, dd (13.0, 3.0)		3.09, dd (22.0, 12.5)
7	150.3, C		162.3, C		165.5, C	
8	147.8, C		104.7, C		78.8, CH	5.15, dd (11.5, 6.5)
9*α*	122.3, CH	5.78, s	80.7, CH	3.05, d (6.0)	41.1, CH_2_	0.99, t (12.5)
9*β*						2.77, dd (12.5, 7.0)
10	38.1, C		38.0, C		38.0, C	
11	122.3, C		125.6, C		123.4, C	
12	170.4, C		170.9, C		174.1, C	
13	55.1, CH_2_	4.48, s	53.0, CH_2_	4.14, s	54.7, CH_2_	4.42, s
14*α*	18.4, CH_3_	0.96, s	9.8, CH_3_	0.90, s	60.1, CH_2_	3.70, d (10.5)
14*β*						3.76, d (10.5)
15*α*	107.8, CH_2_	4.62, d (1.5)	21.3, CH_3_	1.67, s	21.0, CH_3_	1.70, s
15*β*		4.92, d (1.5)				
8-OH				7.09, s		
9-OH				5.49, d (6.5)		
13-OH				5.04, s		

a *Recorded at 500 MHz (^1^H) and 125 MHz (^13^C) in CDCl_3_. ^b^ Recorded at 500 MHz (^1^H) and 125 MHz (^13^C) in DMSO-d_6_*.

**Table 2:** NMR Data of compounds **4–5**.

position	4^a^		5^b^	
	*δ*_C_	*δ*_H_, mult. (*J* in Hz)	*δ*_C_	*δ*_H_, mult. (*J* in Hz)
1*α*	40.6, CH_2_	1.59, m	32.6, CH_2_	1.67, m
1*β*		1.14, m		1.86, td (11.0, 3.5)
2	17.4, CH_2_	1.68, m	23.8, CH_2_	1.64, m
3*α*	41.2, CH_2_	1.42, (13.5, 4.5)	75.2, CH	3.73, s
3*β*		1.68, m		
4	71.6, C		80.1, C	
5	52.3, CH	1.15, m	155.9, C	
6*α*	23.0, CH_2_	2.42, t (13.5)	118.5, CH	7.07, s
6*β*		3.03, dd (13.5, 3.5)		
7	166.3, C		143.8, C	
8	78.8, CH	4.94, dd (12.0, 6.5)	145.6, C	
9*α*	49.7, CH_2_	1.07, t (12.0)	120.9, CH	5.87, s
9*β*		2.18, dd (12.0, 6.5)		
10	34.8, C		42.4, C	
11	122.7, C		116.4, C	
12	174.1, C		171.4, C	
13	54.7, CH_2_	4.40, s	53.6, CH_2_	4.49, s
14	19.0, CH_3_	1.20, s	24.6, CH_3_	1.49, s
15	30.3, CH_3_	1.26, s	18.6, CH_3_	1.54, s
16			49.4, CH_3_	3.13, s

a *Recorded at 500 MHz (^1^H) and 125 MHz (^13^C) in CDCl_3_. ^b^ Recorded at 500 MHz (^1^H) and 125 MHz (^13^C) in CD_3_OD*.

**Table 3:** NMR Data of compounds **6–7**.

position	6^b^		7^a^	
	*δ*_C_	*δ*_H_, mult. (*J* in Hz)	*δ*_C_	*δ*_H_, mult. (*J* in Hz)
1*α*	41.7, CH_2_	2.01, d (14.5)	51.4, CH_2_	2.51(d, 16.2)
1*β*		1.81, dd (14.5, 6.0)		2.56(d, 16.2)
2	65.1, CH	4.41, t (4.5)	196.7, C	
3*α*	132.4, CH	5.95, s	1258.1, CH	6.04(s)
3*β*				
4	134.3, C		159.2, C	
5	157.2, C		45.8, CH	3.00(d, 13.8)
6*α*	113.3, CH	6.95, s	22.3, CH_2_	2.,64(m)
6*β*				3.37(dd, 17.2, 4.2)
7	146.2, C		148.3, C	
8	148.0, C		148.2, C	
9*α*	119.3, CH	5.91, s	119.0, CH	5.77(s)
9*β*				
10	40.8, C		39.1, C	
11	118.5, C		124.0, C	
12	172.9, C		169.6, C	
13	55.2, CH_2_	4.46, s	55.4, CH_2_	4.56(s)
14	29.5, CH_3_	1.42, s	19.3, CH_3_	1.13(s)
15	20.0, CH_3_	2.04, s	21.8, CH_3_	2.07(s)

a *Recorded at 600 MHz (^1^H) and 150 MHz (^13^C) in CDCl_3_. ^b^ Recorded at 500 MHz (^1^H) and 125 MHz (^13^C) in CD_3_OD*.

**Table 4:** NMR Data of compounds **8–10**.

position	8^a^		9^b^		10^a^	
	*δ*_C_	*δ*_H_, mult. (J in Hz)	*δ*_C_	*δ*_H_, mult. (J in Hz)	*δ*_C_	*δ*_H_, mult. (J in Hz)
1*α*	54.0, CH_2_	2.30(d, 16.0)	37.1, CH_2_	1.34(dd, 9.0,4.2)	55.5, CH_2_	2.18, d (16.0)
1*β*		2.39(d, 16.0)				2.28, d (16.0)
2*α*	200.9, C		22.2, CH_2_	1.93(m)	202.6, C	
2*β*				2.04(m)		
3	127.7, CH	5.93(s)	120.4, CH	5.26(s)	127.3, CH	5.86, s
4	164.4, C		131.4, C		168.1, C	
5	49.2, CH	2.67(d, 13.5)	39.7, CH	2.35(d,13.2)	49.6, CH	2.48, m
6*α*	26.1, CH_2_	2.47(t, 13.5)	35.3, CH_2_	1.48(d,13.2)	29.2, CH_2_	1.26, d, 12.0
6*β*		3.51(dd, 13.5, 4.0)		1.63(dd,13.2,4.2)		1.92, d (12.0)
7	167.8, C		75.8, C		42.1, CH	1.60, m
8	79.4, CH	5.08(dd, 12, 6.5)	68.2, CH	3.83(s)	23.9, CH_2_	1.45, m
9*α*	47.1, CH_2_	1.36(t, 12)	43.8, CH_2_	1.38(m)	41.3, CH_2_	1.45, td (12.5, 3.5)
9*β*		2.35(dd, 12, 6.5)		6.06(s)		1.55, m
10	39.7, C		32.9, C		39.2, C	
11	125.5, C		115.3, C		41.3, CH	1.63, m
12*α*	175.6, C		108.9, CH_2_	5.12(d,1.8)	66.5, CH_2_	3.46, q (6.0)
12*β*				5.19(d,1.8)		3.55, q (6.0)
13	54.6, CH_2_	4.35(s)	61.6, CH_2_	4.06(d,5.4)	14.0, CH_3_	0.93, d (7.0)
14	17.4, CH_3_	1.09(s)	15.7, CH_3_	0.8(s)	17.2, CH_3_	0.86, s
15	22.3, CH_3_	2.06(s)	21.0, CH_3_	1.53(s)	22.4, CH_3_	1.95, s
7-OH				3.83 (s)		
8-OH				4.70 (s)		
13-OH				4.13 (s)		

a *Recorded at 500 MHz (^1^H) and 125 MHz (^13^C) in CD_3_OD. ^b^ Recorded at 600 MHz (^1^H) and 150 MHz (^13^C) in DMSO-d_6_*.

**Figure 2:**
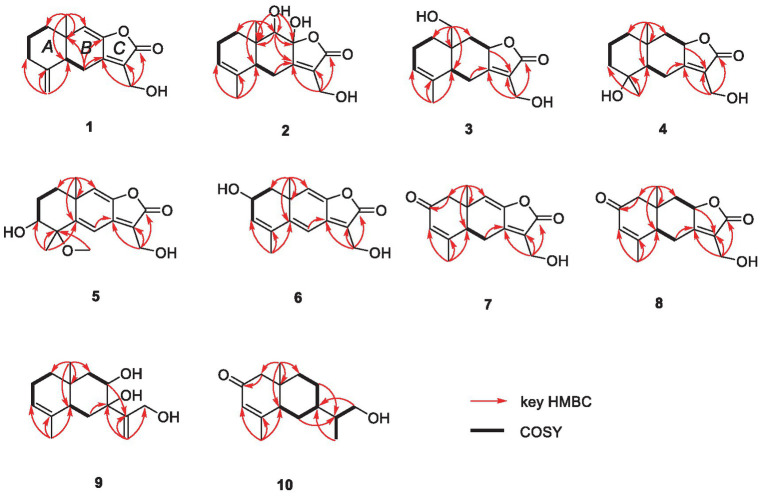
Key HMBC and COSY correlations of **1–10**.

**Figure 3:**
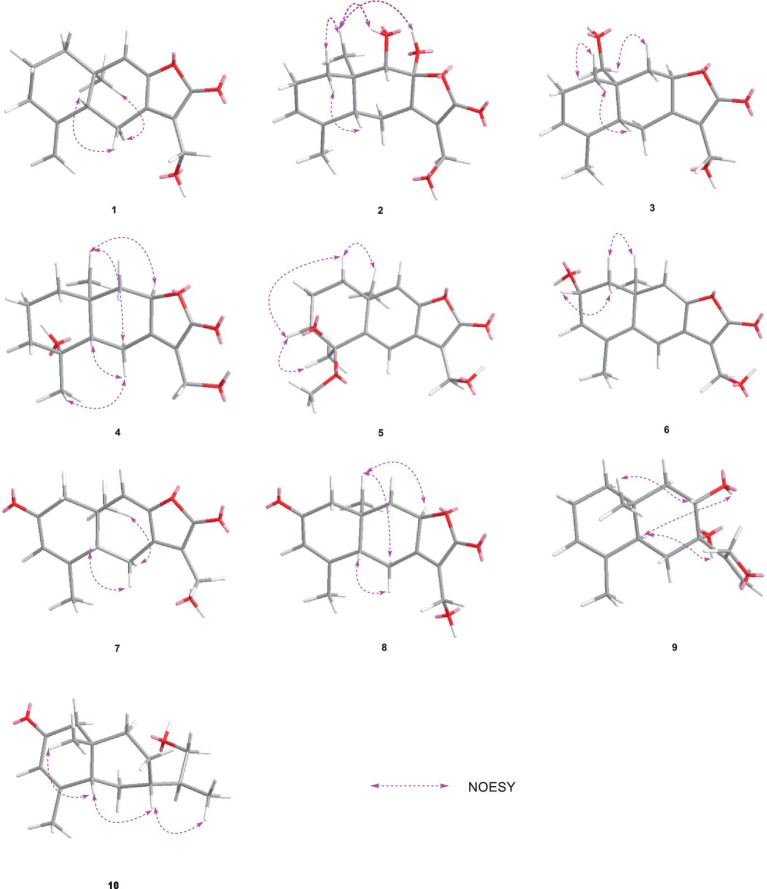
Key NOESY correlations of **1–10**.

**Figure 4:**
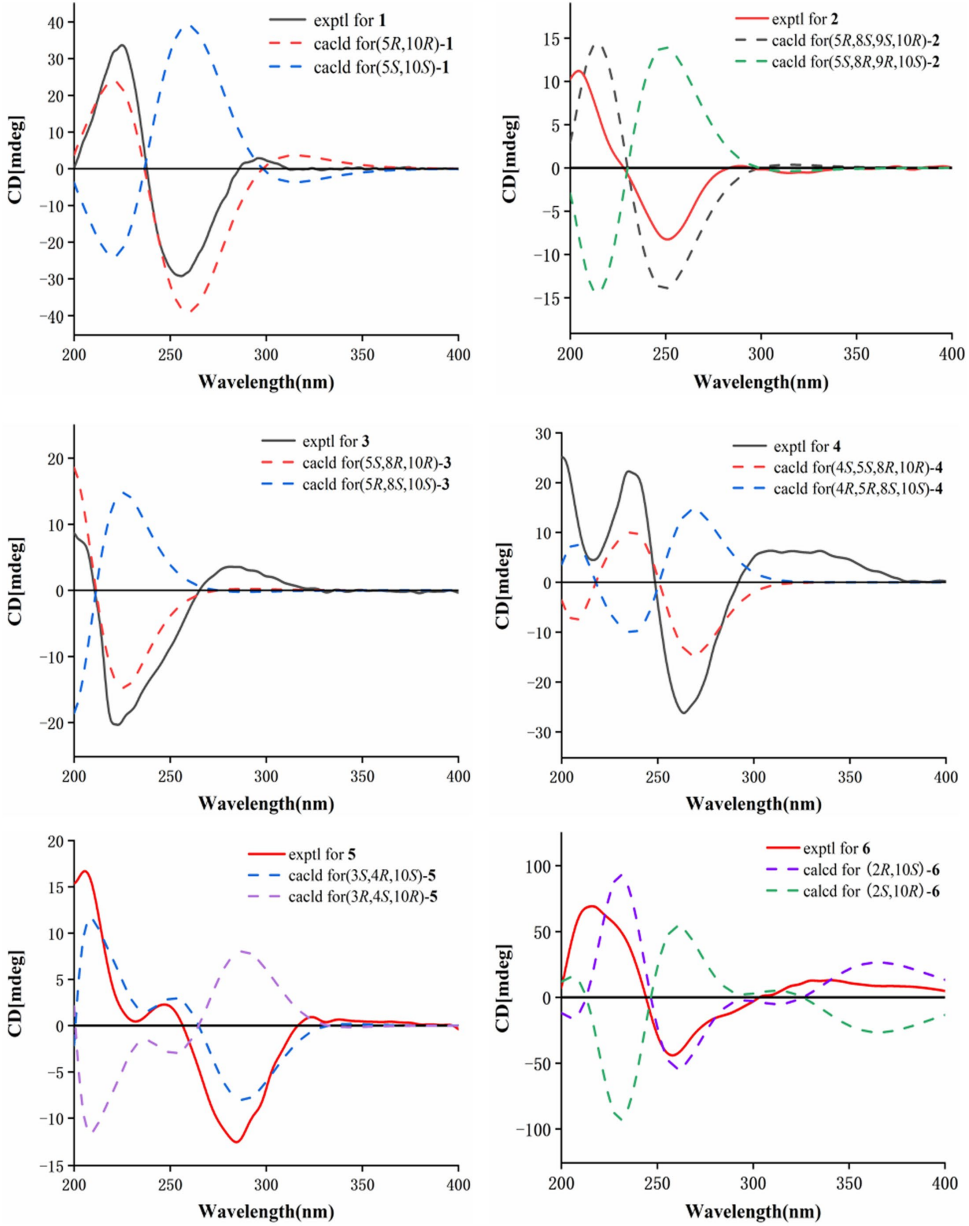
Calculated and experimental ECD spectra of **1–6**.

HMBC correlations from H_2_-6 to C-5, C-7 (*δ*_C_ 150.3), and C-10, and from H-9 to C-7, C-8 (*δ*_C_ 147.8), and C-10, H_3_-14 to C-1, C-9, and C-10, and from H_3_-15 to C-3, C-4, and C-5, together with the COSY correlations of H-1/H-2, H-2/H-3, and H-5/H_2_-6 (Figure 2), established the two six-membered rings A and B with the methyl group CH_3_-14 attached at C-10. Moreover, the HMBC correlations from H_2_-13 (*δ*_H_ 4.48) to C-7, C-11 (*δ*_C_ 122.3), and C-12 (*δ*_C_ 170.4) and the presence of downfield C-8 established a C ring. The relative configuration of **1** was deduced through NOESY correlations of H-6*α* (*δ*_H_ 2.61)/H_3_-14 (*δ*_H_ 0.96) and H-6*β* (*δ*_H_ 2.88)/H-5 (*δ*_H_ 2.36) (Figure 3). The absolute configuration of **1** was determined to be 5*R*,10*R* by comparing its specific rotation (${\left[\alpha \right]}_{D}^{25}$ −27.9, *c* 0.1, MeOH) with the known compound atractylenolide II (${\left[\alpha \right]}_{D}^{25}$ +22.2, *c* 1.05, MeOH) (Li and Yang, 2018). The similarity between the calculated and experimental ECD spectra further provided evidence for the determination of the absolute configuration of **1** (Figure 4).

Eutypellaolide B (**2**) was obtained as yellowish oil. The molecular formula of **2** was established as C_15_H_20_O_5_ by HRESIMS data (*m/z* 303.1203 [M + Na]^+^). The NMR data of **2** were almost identical to those of **13**, except for the presence of the 9-OH proton resonance [*δ*_H_ 5.78 (d, 6.5)], which was supported by the HMBC correlations of 9-OH to C-8 (*δ*_C_ 104.7), C-9 (*δ*_C_ 80.7) and C-10 (*δ*_C_ 38.0). The relative configuration of H-5 and H-9 was determined to be *β*-oriented based on the NOESY correlation of H-1*β*/H-5 and H-9. Additionally, 8-OH, 9-OH, and H_3_-14 were established to be *α*-oriented based on NOESY correlations of H-1*α*/H_3_-14 and 8-OH/H_3_-14 in DMSO-*d*_6_. Furthermore, the similarity between the calculated and experimental ECD spectra further confirmed the absolute configurations of **2** as 5*R*,8*S*,9*S*,10*R* (Figure 4).

Eutypellaolide C (**3**) was obtained as a yellowish oil. The molecular formula was deduced as C_15_H_20_O_4_ based on HRESIMS (*m/z* 287.1266 [M + Na]^+^). The ^1^H and ^13^C NMR spectra of **3** displayed remarkable similarity to those of 13-hydroxy-3,7(11)-eudesmadien-2,8-olide (Wang et al., 2017), except for the presence of an additional hydroxy group at C-14 (*δ*_C_ 60.1). The methylene carbon C-14 was attached to C-10, which was supported by the HMBC correlations from H_2_-14 (*δ*_H_, 3.73) to C-1 (*δ*_C_ 31.4), C-5 (*δ*_C_ 46.6), C-9 (*δ*_C_ 41.1), and C-10 (*δ*_C_ 38.0). Thus, the planar configuration of **3** is presented in Figure 2. The relative configuration of **3** was determined by the NOESY correlations of H_2_-14/H-1*α*, H_2_-14/H-8, and H-5/H-1*β*. The absolute configuration of **3** was subsequently determined to be 5*S*,8*R*,10*R* based on a comparison of its specific rotation (${\left[\alpha \right]}_{D}^{25}$ –31.9, MeOH, *c* 0.1) with that of 13-hydroxy-3,7(11)-eudesmadien-12,8-olide (${\left[\alpha \right]}_{D}^{20}$ –191.4, MeOH, *c* 0.5) (Wang et al., 2017). The validation of the absolute configuration of **3** was strengthened by the similarity observed between the calculated ECD spectrum and the experimental CD spectrum (Figure 4).

Eutypellaolide D (**4**) was obtained as a yellowish oil, with a molecular formula of C_15_H_22_O_4_ as determined by HRESIMS (*m/z* 289.1401 [M + Na]^+^). Comparison of the ^1^H and ^13^C NMR spectra of **4** with those of 4*β*-hydroxy-5*α*,8*β*(*H*)-eudesm-7(11)-en-8,12-olide (Zhang et al., 2012), with a difference in the presence of a hydroxyl group substituted at C-13 (*δ*_C_ 54.7) in **4**, was supported by the HMBC correlations from H_2_-13 to C-7 (*δ*_C_ 166.3), C-11 (*δ*_C_ 122.7), and C-12 (*δ*_C_ 174.1). The relative configuration of **4** was established by NOESY correlations of H-5/H-6*β*, H_3_-15/H-6*β*, H-8/H_3_-14, and H-8/H-6*α*, which indicated that H-5 and H_3_-15 were in the same orientation, while H-8 and H_3_-14 had opposite orientation. The absolute configuration of **4** was established as 4*S*,5*S*,8*R*,10*R* by comparing its specific rotation data (${\left[\alpha \right]}_{D}^{25}$ −47.2, MeOH, *c* 0.1) with that of 4*β*-hydroxy-5*α*,8*β*(*H*)-eudesm-7(11)-en-8,12-olide (${\left[\alpha \right]}_{D}^{25}$ +20.0, *c* 0.57, MeOH) (Wang et al., 2017). The agreement between the calculated ECD spectrum and the experimental CD spectrum also supported the absolute configuration of **4** (Figure 4).

Eutypellaolide E (**5**) was isolated as a yellowish oil and had a molecular formula of C_16_H_20_O_5_ based on HRESIMS (*m/z* 315.1204 [M + Na]^+^). The NMR data of **5** closely resembled those of **12** (Wang et al., 2017), except for the presence of a hydroxy group at C-3 (*δ*_C_ 75.2) and methoxy group at C-4 (*δ*_C_ 80.1) in **5** and the absence of two olefinic carbons (*δ*_C_ 131.2 and *δ*_C_ 131.4) in **12**. The C-3 was attached to C-16 (*δ*_C_ 49.4) via the C-4, which was confirmed by the HMBC correlation from H_3_-16 (*δ*_H_, 3.13, s) to C-4 (*δ*_C_ 80.1). The NOESY correlations from H_3_-14 to H-1*α* and from H-3 to H-1*α* and H_3_-15 suggested that H-3/H_3_-14/H_3_-15 was located at the same orientation. Furthermore, a comparison between the calculated and the experimental ECD spectra confirmed the absolute configurations of **5** as 3*S*,4*R*,10*S* (Figure 4).

Eutypellaolide *F* (**6**), obtained as a yellowish oil, had the molecular formula of C_15_H_16_O_4_, according to its positive HRESIMS (*m/z* 283.0942 [M + Na]^+^). Comparative analysis of the data for **6** with that of **12** revealed a high degree of similarity (Wang et al., 2017), except for the hydroxy group substituted at C-2 (*δ*_C_ 65.1) in **6** instead of the H-2 in **12**, which was confirmed by the COSY correlations between H-1/H-2/H-3, and the HMBC correlations from H-3 to C-1 (*δ*_C_ 41.7) and C-2 (*δ*_C_ 65.1). The same orientation of 2-OH and H_3_-14 was deduced from the NOESY correlation between H-1*α* [*δ*_H_, 2.01 (d, 14.5 Hz)] and H_3_-14 and between H-1*β* [*δ*_H_, 1.81 (dd, 14.5, 6.0 Hz)] and H-2 [*δ*_H_, 4.41 (t, 4.5 Hz)]. A comparison between the calculated and experimental ECD spectrum of **6** determined its absolute configuration as 2*R*,10*S* (Figure 4).

**Figure 5:**
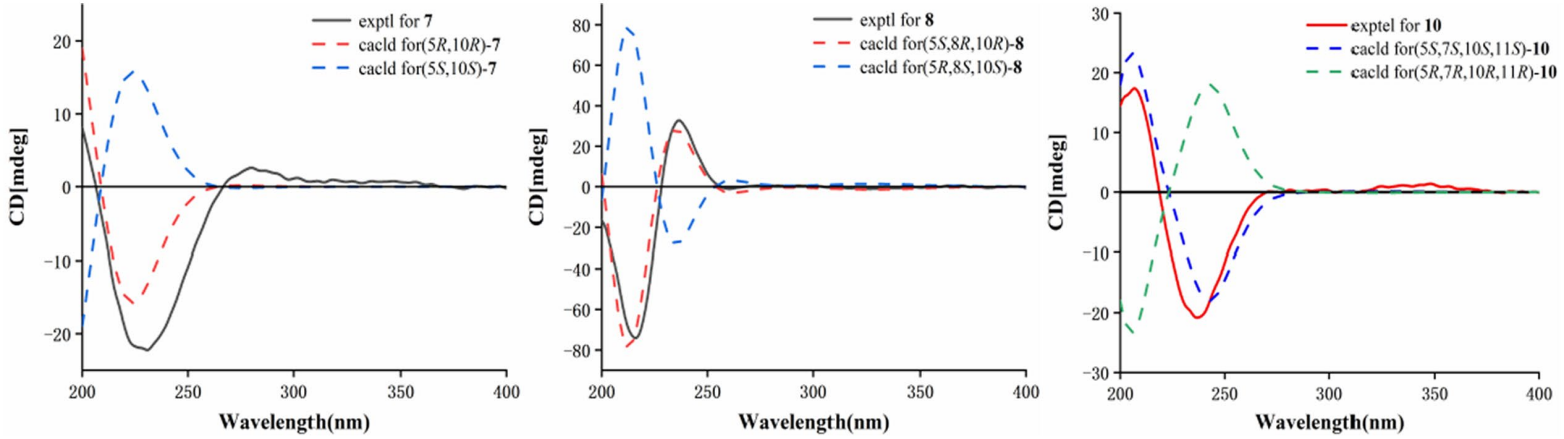
Calculated and experimental ECD spectra of **7**, **8**, and **10**.

Eutypellaolide G (**7**) was obtained as a yellowish oil, and its molecular formula was deduced as C_15_H_16_O_4_ due to its HRESIMS (*m/z* 283.0947 [M + Na]^+^), indicating eight degrees of unsaturation. Notably, the NMR data of **7** closely resembled that of chlorantholide A (Wang et al., 2012), except for the discernible hydroxy group at C-13 in **7**, which was verified by the HRESIMS data and the HMBC correlations from H_2_-13 (*δ*_H_ 4.56) to C-7 (*δ*_C_ 148.3), C-11 (*δ*_C_ 124.0), and C-12 (*δ*_C_ 169.6). The NOESY correlations from H-6*α* (*δ*_H_ 2.64) to H_3_-14 and H-6*β* (*δ*_H_ 3.37) to H-5 confirmed that H_3_-14 and H-5 were in opposite orientations. The absolute configuration of **7** was determined by comparing its specific rotation with chlorantholide A. A comparison of the specific rotation between **7** (${\left[\alpha \right]}_{D}^{25}$ −9.3, MeOH, *c* 0.1) and chlorantholide A (${\left[\alpha \right]}_{D}^{21}$+5.0, MeOH, *c* 0.2) assigned the absolute configuration of **7** as 5*R*,10*R*. The comparison between the calculated and experimental ECD spectra further supported the absolute configuration of **7** (Figure 5).

Eutypellaolide H (**8**), obtained as a yellowish oil, had the molecular formula of C_15_H_18_O_4_ based on the positive HRESIMS (*m/z* 285.1103 [M + Na]^+^). The overall NMR data of **8** indicated a structure similar to **7**, with a notable difference in the absence of the double bond between C-8 (*δ*_C_ 148.2) and C-9 (*δ*_C_ 119.0) in **7**, confirmed by the HMBC correlations from H-8 (*δ*_H_ 5.08, dd, 12.0, 6.5 Hz) to C-7(*δ*_C_ 167.8), C-9 (*δ*_C_ 47.1), C-11 (*δ*_C_ 125.5), and C-12 (*δ*_C_ 175.6) and COSY correlations from H-8 to H-9. The detectable NOESY correlations of H-8/H-6α, H-8/H3-14, and H-6β/H-5 confirmed that H-8 and H3-14 are oriented in the same way, while H-5 are oriented in an opposite direction. The absolute configuration of **8** could be assigned as 5*S*,8*R*,10*R* by a comparison of the specific rotation (${\left[\alpha \right]}_{D}^{25}$ −91.2, MeOH, *c* 0.1) with that of chlorantholide B (${\left[\alpha \right]}_{D}^{20}$ +74.7, MeOH, *c* 0.1) (Wang et al., 2012). The similarity between the calculated ECD spectrum and the experiment further supported the absolute configuration of **8** (Figure 5).

Eutypellaolide I (**9**) was isolated as a white powder, with a molecular formula of C_15_H_24_O_3_ as determined by HRESIMS and NMR data, indicating the presence of four degrees of unsaturation. The ^1^H NMR spectrum showed terminal methylene singlet at *δ*_H_ 5.29 and *δ*_H_ 5.36 and exocyclic methylene at *δ*_H_ 4.20 and *δ*_H_ 4.30. The ^13^C NMR spectrum for **9** revealed the presence of four olefinic carbons at *δ*_C_ 153.5, 133.9, 121.3, and 114.8, one oxygenated quaternary carbon at *δ*_C_ 76.9, one oxymethine carbon at *δ*_C_ 70.4, one oxygenated methylene at *δ*_C_ 64.7, and two methyl carbons at *δ*_C_ 15.9 and *δ*_C_ 21.2, and the remaining two degrees of unsaturation implied that **9** was likely to be dicyclic sesquiterpenes. The HMBC correlations from H_3_-15 to C-3 and C-4, from H_3_-14 to C-1, C-5, C-9, and C-10, from H_2_-6 to C-4, C-7, C-8, and C-10, and from H_2_-9 to C-5, C-7, C-8, and C-10, together with the COSY correlations between H-1/H-2/H-3, H-5/H_2_-6, and H-8/H_2_-9, confirmed the presence of a six-membered ring. Additional HMBC correlations from H-12a/12b to C-7, C-11, and C-12 and from H-13 to C-7 suggested direct linkages between C-7 and C-11. The NOESY correlations from H_3_-14 to H-8 indicated that these protons are on the same side, and those from H-5 to 7-OH and 8-OH confirmed that they were in opposite orientations. The absolute configuration of **9** was subsequently determined to be 5*R*,7*S*,8*R*,10*S* based on a comparison of the specific rotation (${\left[\alpha \right]}_{D}^{25}$ +0.6, MeOH, *c* 0.1) with that of rhaponticol (${\left[\alpha \right]}_{D}^{20}$ +33.3, MeOH, *c* 0.12) (Cheng et al., 1995), following the method as the cases of compounds **1**, **3**, **4**, **7**, and **8**.

Eutypellaolide J (**10**) was a colorless oil and exhibited a planar structure identical to that of thomimarine E (Afiyatullov et al., 2017). However, the chemical shift of C-11 (*δ*_C_ 41.3) in **10** was different from C-11 (*δ*_C_ 33.1) in thomimarine E, confirming that **10** and thomimarine E have different configurations, which was supported by the NOESY spectrum correlations from H-5 (*δ*_H_ 2.48) to H-7 (*δ*_H_ 1.60) and H_3_-14 (*δ*_H_ 0.86) and from H-7 to H_3_-13 (*δ*_H_ 0.93), indicating that these protons were on the same side. The absolute configuration of compound **10** was established as 5*S*,7*S*,10*S*,11*S* by a comparison between experimental and calculated ECD (Figure 5).

The known compounds *eut*-Guaiane (**11**), 13-hydroxy-3,5,8,7(11)-eudesmatetraen-12,8-olide (**12**), 8,13-dihydroxy-3,7(11)-eudesmadien-12,8-olide (**13**), and 2-one-13hydroxy-3,5,8,7(11)-eudesmatetraen-12,8-olide (**14**) were also isolated from *Eutypella* sp. D-1 and were completely characterized by comparison of their NMR data with that previously reported (Wang et al., 2017; Zhou et al., 2017).

All the isolated compounds **1**–**14** were screened for their antibacterial activity against *Staphylococcus aureus* (ATCC 27217), *Bacillus subtilis* (ATCC 21951), *Pseudomonas aeruginosa* (ATCC 27853), *Vibrio vulnificus* (ATCC 27562), and *Vibrio parahaemolyticus* (ATCC 17802). Among them, compounds **1** and **11** displayed potent activity against *B. subtilis* and *S. aureus*, with MIC values of 2 μg/mL for both strains (Table 5). Additional immunosuppressive activity against ConA-induced T-cell proliferation for **1**–**14** was also tested. Among them, only **9** exhibited immunosuppressive activity, with inhibitory rates of 61.7% observed at a concentration of 19.8 μM. Compounds **1**–**14** were subjected to preliminary screening for their inhibitory activity against PTP1B. However, only compounds **5**, **11**, and **14** exhibited moderate activity, with IC_50_ values of 44.8, 43.2, and 49.5 μM, respectively (Table 6).

**Table 5:** Antibacterial activities of compounds **1, 9, 10, and 11**.

compound	MIC (μg /ml)				
	*B. subtilis*	*S. aureus*	*P. aeruginosa*	*V. vulnficus*	*V. parahemolyticus*
**1**	2	2	8	32	32
**9**	16	16	>64	>64	>64
**10**	32	32	>64	>64	>64
**11**	2	2	>64	32	32
levofloxacin	0.5	0.5	0.5	0.5	0.5

**Table 6:** Inhibition against PTP1B enzyme of compounds **5, 11**, and **14**.

Compound	IC_50_ ± SD (μM)
**5**	44.8 ± 1.13
**11**	43.2 ± 0.93
**14**	49.5 ± 0.88
oleanolic acid	11.5 ± 1.33

## 4 Conclusion

In summary, the OSMAC approach effectively induced chemical diversities of the polar fungus *Eutypella* sp. D-1, using a modified solid nutrient medium, to produce fourteen sesquiterpene compounds. Among them, there were ten new sesquiterpenes eutypellaolides A − J (**1**–**10**) and four known 12,8-eudesmanolide compounds **11**–**14**. Fortunately, the production of compound **11**, which exhibits excellent antibacterial activity, increased sharply. Interestingly, these new metabolites were only detected in the solid nutrient medium and were not produced when the fungus was cultivated in potato dextrose broth (PDB) or other liquid media (Lu et al., 2014; Zhou et al., 2017; Wang et al., 2018). Therefore, it could be concluded that the OSMAC approach should be a feasible and effective strategy to trigger the production of bioactive secondary metabolites from the polar fungi. Compounds **5**, **11**, and **14** possess an *α*,*β*-unsaturated *γ*-lactone structure, which serves as a crucial pharmacophore for significant PTP1B inhibitory activity, and this characteristic was shared with sesterterpene phyllofolactones A and phyllofolactones F, as reported in the literature (Abdjul et al., 2015). The findings from this study contribute to the expanding knowledge of natural products from polar fungi and their potential for discovery as new drug leads.

## Data availability statement

The original contributions presented in the study are included in the article/Supplementary material, further inquiries can be directed to the corresponding authors.

## Author contributions

ZN: Data curation, Investigation, Methodology, Writing – original draft. BH: Supervision, Validation, Writing – original draft. Y-YS: Investigation, Writing – review & editing. J-FD: Software, Writing – review & editing. X-YH: Data curation, Formal Analysis, Writing – review & editing. X-LL: Project administration, Writing – review & editing. Z-FY: Visualization, Writing – review & editing. YH: Conceptualization, Writing – review & editing. B-HJ: Resources, Visualization, Writing – review & editing. H-BY: Conceptualization, Funding acquisition, Resources, Writing – review & editing. X-YL: Conceptualization, Funding acquisition, Methodology, Resources, Writing – review & editing.
