# Supplementary material for: Eutypellaolides A–J, Sesquiterpene diversity expansion of the polar fungus Eutypella sp. D-1
Source: Front Microbiol. 2024 Jan 25;15:1349151. doi: 10.3389/fmicb.2024.1349151 (PMC10850561; doi:10.3389/fmicb.2024.1349151)
Supplement: Supplementary file 1 [file Data_Sheet_1.PDF]

Supplementary data

Eutypellaolides A–J, Sesquiterpene Diversity Expansion of the Polar Fungus *Eutypella* sp. D-1

Zhe Ning [1,†], Bo Hu [1,†], Yuan-Yuan Sun [1], Jin-Feng Ding [1], Xiang-Ying Han [1], Xiao-Ling Lu [2], Zi-Fei Yin [3], Ying He [1], Bing-Hua Jiao [2], Hao-Bing Yu [1,*], Xiao-Yu Liu [1,*]

* Correspondence: yuhaobing1986@126.com (H.-B.Y.), biolxy@163.com (X.-Y.L.);
† These authors contributed equally to this work
1 Department of Marine Biomedicine and Polar Medicine, Naval Medical Center of PLA, Naval Medical University, Shanghai 200433, China
2 Department of Biochemistry and Molecular Biology, College of Basic Medical Sciences, Naval Medical University, Shanghai 200433, China
3 School of Traditional Chinese Medicine, Naval Medical University, Shanghai 200433, China

S1. Quantum chemical CD calculation of compound **1**.
S2. Quantum chemical CD calculation of compound **2**.
S3. Quantum chemical CD calculation of compound **3**.
S4. Quantum chemical CD calculation of compound **4**.
S5. Quantum chemical CD calculation of compound **5**.
S6. Quantum chemical CD calculation of compound **6**.
S7. Quantum chemical CD calculation of compound **7**.
S8. Quantum chemical CD calculation of compound **8**.
S9. Quantum chemical CD calculation of compound **10**.
S10. ^{1}H NMR spectrum of Eutypellaolide A (**1**) in $CDCl_3$
S11. ^{13}C NMR spectrum of Eutypellaolide A (**1**) in $CDCl_3$
S12. DEPT135 spectrum of Eutypellaolide A (**1**) in $CDCl_3$
S13. HSQC spectrum of Eutypellaolide A (**1**) in $CDCl_3$
S14. COSY spectrum of Eutypellaolide A (**1**) in $CDCl_3$
S15. HMBC spectrum of Eutypellaolide A (**1**) in $CDCl_3$
S16. NOESY spectrum of Eutypellaolide A (**1**) in $CDCl_3$
S17. HRESIMS of Eutypellaolide A (**1**)
S18. UV spectrum of Eutypellaolide A (**1**) in MeOH
S19. IR spectrum of Eutypellaolide A (**1**)
S20. ^{1}H NMR spectrum of Eutypellaolide B (**2**) in DMSO-*d6*
S21. ^{13}C NMR spectrum of Eutypellaolide B (**2**) in DMSO-*d6*
S22. DEPT135 spectrum of Eutypellaolide B (**2**) in DMSO-*d6*
S23. HSQC spectrum of Eutypellaolide B (**2**) in DMSO-*d6*
S24. COSY spectrum of Eutypellaolide B (**2**) in DMSO-*d6*
S25. HMBC spectrum of Eutypellaolide B (**2**) in DMSO-*d6*
S26. NOESY spectrum of Eutypellaolide B (**2**) in DMSO-*d6*
S27. HRESIMS of Eutypellaolide B (**2**)
S28. UV spectrum of Eutypellaolide B (**2**) in MeOH
S29. IR spectrum of Eutypellaolide B (**2**)
S30. ^{1}H NMR spectrum of Eutypellaolide C (**3**) in $CDCl_3$
S31. ^{13}C NMR spectrum of Eutypellaolide C (**3**) in $CDCl_3$
S32. DEPT135 spectrum of Eutypellaolide C (**3**) in $CDCl_3$
S33. HSQC spectrum of Eutypellaolide C (**3**) in $CDCl_3$
S34. COSY spectrum of Eutypellaolide C (**3**) in $CDCl_3$
S35. HMBC spectrum of Eutypellaolide C (**3**) in $CDCl_3$
S36. NOESY spectrum of Eutypellaolide C (**3**) in $CDCl_3$
S37. HRESIMS of Eutypellaolide C (**3**)
S38. UV spectrum of Eutypellaolide C (**3**) in MeOH
S39. IR spectrum of Eutypellaolide C (**3**)
S40. ^{1}H NMR spectrum of Eutypellaolide D (**4**) in $CDCl_3$
S41. ^{13}C NMR spectrum of Eutypellaolide D (**4**) in $CDCl_3$
S42. DEPT135 spectrum of Eutypellaolide D (**4**) in $CDCl_3$
S43. HSQC spectrum of Eutypellaolide D (**4**) in $CDCl_3$
S44. COSY spectrum of Eutypellaolide D (**4**) in $CDCl_3$

S45. HMBC spectrum of Eutypellaolide D (**4**) in $CDCl_3$
S46. NOESY spectrum of Eutypellaolide D (**4**) in $CDCl_3$
S47. HRESIMS of Eutypellaolide D (**4**)
S48. UV spectrum of Eutypellaolide D (**4**) in MeOH
S49. IR spectrum of Eutypellaolide D (**4**)
S50. ^{1}H NMR spectrum of Eutypellaolide E (**5**) in MeOH
S51. ^{13}C NMR spectrum of Eutypellaolide E (**5**) in MeOH
S52. DEPT135 spectrum of Eutypellaolide E (**5**) in MeOH
S53. HSQC spectrum of Eutypellaolide E (**5**) in MeOH
S54. COSY spectrum of Eutypellaolide E (**5**) in MeOH
S55. HMBC spectrum of Eutypellaolide E (**5**) in MeOH
S56. NOESY spectrum of Eutypellaolide E (**5**) in MeOH
S57. HRESIMS of Eutypellaolide E (**5**)
S58. UV spectrum of Eutypellaolide E (**5**) in MeOH
S59. IR spectrum of Eutypellaolide E (**5**)
S60. ^{1}H NMR spectrum of Eutypellaolide F (**6**) in MeOH
S61. ^{13}C NMR spectrum of Eutypellaolide F (**6**) in MeOH
S62. DEPT135 spectrum of Eutypellaolide F (**6**) in MeOH
S63. HSQC spectrum of Eutypellaolide F (**6**) in MeOH
S64. COSY spectrum of Eutypellaolide F (**6**) in MeOH
S65. HMBC spectrum of Eutypellaolide F (**6**) in MeOH
S66. NOESY spectrum of Eutypellaolide F (**6**) in MeOH
S67. HRESIMS of Eutypellaolide F (**6**)
S68. UV spectrum of Eutypellaolide F (**6**) in MeOH
S69. IR spectrum of Eutypellaolide F (**6**)
S70. ^{1}H NMR spectrum of Eutypellaolide G (**7**) in $CDCl_3$
S71. ^{13}C NMR spectrum of Eutypellaolide G (**7**) in $CDCl_3$
S72. DEPT135 spectrum of Eutypellaolide G (**7**)) in $CDCl_3$
S73. HSQC spectrum of Eutypellaolide G (**7**) in $CDCl_3$
S74. COSY spectrum of Eutypellaolide G (**7**) in $CDCl_3$
S75. HMBC spectrum of Eutypellaolide G (**7**) in $CDCl_3$
S76. NOESY spectrum of Eutypellaolide G (**7**) in $CDCl_3$
S77. HRESIMS of Eutypellaolide G (**7**)
S78. UV spectrum of Eutypellaolide G (**7**) in MeOH
S79. IR spectrum of Eutypellaolide G (**7**)
S80. ^{1}H NMR spectrum of Eutypellaolide H (**8**) in MeOH
S81. ^{13}C NMR spectrum of Eutypellaolide H (**8**) in MeOH
S82. DEPT135 spectrum of Eutypellaolide H (**8**) in MeOH
S83. HSQC spectrum of Eutypellaolide H (**8**) in MeOH
S84. COSY spectrum of Eutypellaolide H (**8**) in MeOH
S85. HMBC spectrum of Eutypellaolide H (**8**) in MeOH
S86. NOESY spectrum of Eutypellaolide H (**8**) in MeOH
S87. HRESIMS of Eutypellaolide H (**8**)
S88. UV spectrum of Eutypellaolide H (**8**) in MeOH

S89. IR spectrum of Eutypellaolide H (**8**)
S90. ^{1}H NMR spectrum of Eutypellaolide I (**9**) in DMSO-*d6*
S91. ^{13}C NMR spectrum of Eutypellaolide I (**9**) in DMSO-*d6*
S92. DEPT135 spectrum of Eutypellaolide I (**9**) in DMSO-*d6*
S93. HSQC spectrum of Eutypellaolide I (**9**) in DMSO-*d6*
S94. COSY spectrum of Eutypellaolide I (**9**) in DMSO-*d6*
S95. HMBC spectrum of Eutypellaolide I (**9**) in DMSO-*d6*
S96. NOESY spectrum of Eutypellaolide I (**9**) in DMSO-*d6*
S97. HRESIMS of Eutypellaolide I (**9**)
S98. UV spectrum of Eutypellaolide I (**9**) in MeOH
S99. IR spectrum of Eutypellaolide I (**9**)
S100. ^{1}H NMR spectrum of Eutypellaolide J (**10**) in MeOH
S101. ^{13}C NMR spectrum of Eutypellaolide J (**10**) in MeOH
S102. DEPT135 spectrum of Eutypellaolide J (**10**) in MeOH
S103. HSQC spectrum of Eutypellaolide J (**10**) in MeOH
S104. COSY spectrum of Eutypellaolide J (**10**) in MeOH
S105. HMBC spectrum of Eutypellaolide J (**10**) in MeOH
S106. NOESY spectrum of Eutypellaolide J (**10**) in MeOH
S107. HRESIMS of Eutypellaolide J (**10**)
S108. UV spectrum of Eutypellaolide J (**10**) in MeOH
S109. IR spectrum of Eutypellaolide J (**10**)
S110. The HPLC analysis of the extract at different culture conditions compared with standard substances

S1. Quantum chemical CD calculation of compound **1**.

Conformational analysis was initially performed using Spartan'14 software (Wavefunction, Inc., Irvine, CA, USA) at MMFF94 force field. The conformers with Boltzmann-population of over 5% were chosen for ECD calculations, and then the conformers were initially optimized at B3LYP/DGTZVP level in MeOH using the intergral equation formalism polarizable continuum model (IEFPCM). Harmonic vibration frequencies were calculated to confirm the stability of these conformers. As revealed by the frequency analysis, no imaginary frequencies were observed in ground states. The theoretical calculation of ECD was conducted in MeOH using Time-dependent Density functional theory (TD-DFT) at the B3LYP/DGTZVP level for all conformers of compound **1**. The CD spectra were generated by the program GaussView 6.0 (University of Würzburg, Würzburg, Germany) using a Gaussian band shape with 0.3 eV exponential half-width from dipole-length dipolar and rotational strengths.

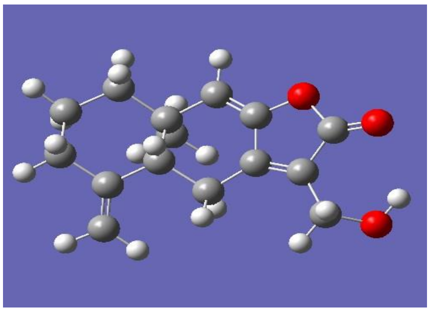

1a

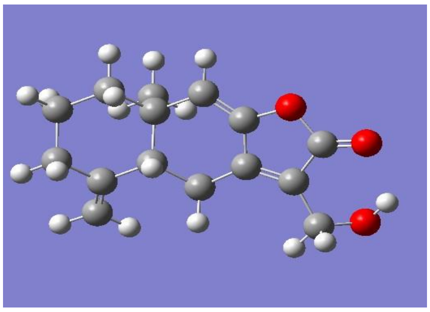

1b

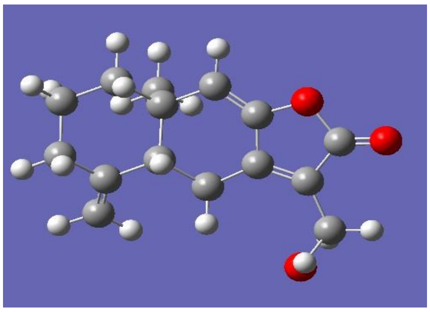

1c

	Conformer	ΔE(kcal/mol)	Population(%)
Compound Model-**1**	**1a**	0.00	34.2
	**1b**	0.01	33.5
	**1c**	0.03	32.3

S2. Quantum chemical CD calculation of compound **2**.

Conformational analysis was initially performed using Spartan’14 software (Wavefunction, Inc., Irvine, CA, USA) at MMFF94 force field. The conformers with Boltzmann-population of over 5% were chosen for ECD calculations, and then the conformers were initially optimized at B3LYP/6-311G level in MeOH using the intergral equation formalism polarizable continuum model (IEFPCM). Harmonic vibration frequencies were calculated to confirm the stability of these conformers. As revealed by the frequency analysis, no imaginary frequencies were observed in ground states. The theoretical calculation of ECD was conducted in MeOH using Time-dependent Density functional theory (TD-DFT) at the B3LYP/6-311G+ level for all conformers of compound **2**. The CD spectra were generated by the program GaussView 6.0 (University of Würzburg, Würzburg, Germany) using a Gaussian band shape with 0.3 eV exponential half-width from dipole-length dipolar and rotational strengths.

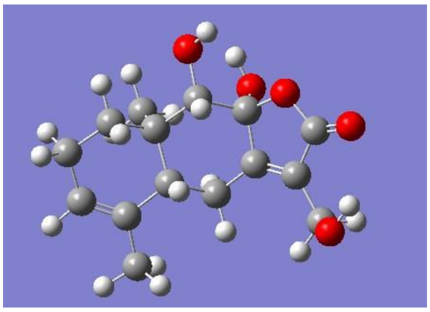

2a

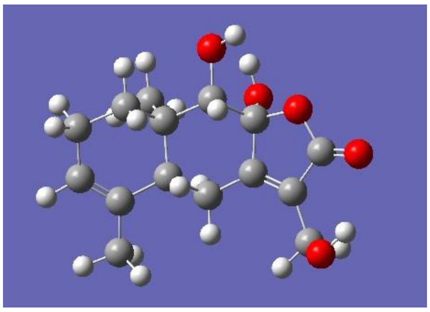

2b

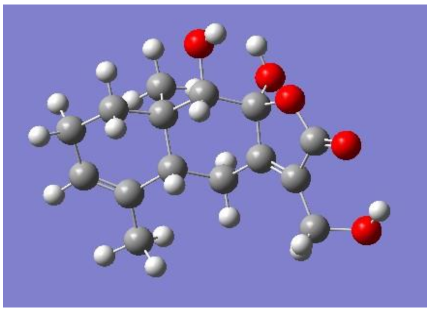

2c

	Conformer	ΔE(kcal/mol)	Population(%)
Compound Model-**2**	**2a**	0.00	39.0
	**2b**	0.00	38.9
	**2c**	0.34	22.1

S3. Quantum chemical CD calculation of compound **3**.

Conformational analysis was initially performed using Spartan’14 software (Wavefunction, Inc., Irvine, CA, USA) at MMFF94 force field. The conformers with Boltzmann-population of over 5% were chosen for ECD calculations, and then the conformers were initially optimized at B3LYP/DGTZVP level in MeOH using the intergral equation formalism polarizable continuum model (IEFPCM). Harmonic vibration frequencies were calculated to confirm the stability of these conformers. As revealed by the frequency analysis, no imaginary frequencies were observed in ground states. The theoretical calculation of ECD was conducted in MeOH using Time-dependent Density functional theory (TD-DFT) at the B3LYP/DGTZVP level for all conformers of compound **3**. The CD spectra were generated by the program GaussView 6.0 (University of Würzburg, Würzburg, Germany) using a Gaussian band shape with 0.3 eV exponential half-width from dipole-length dipolar and rotational strengths.

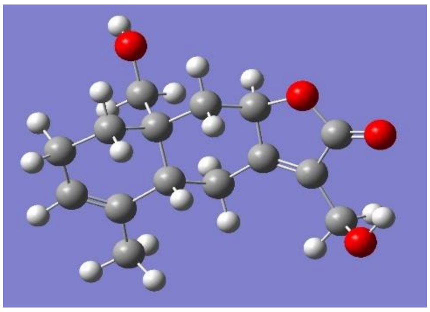

3a

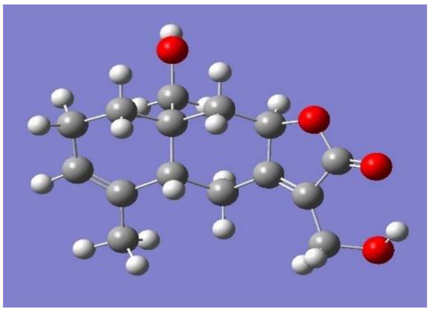

3b

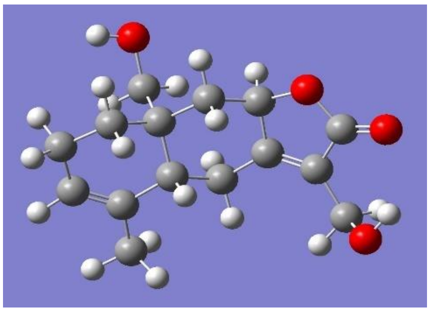

3c

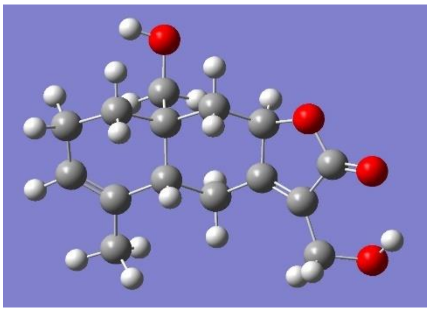

3d

	Conformer	ΔE(kcal/mol)	Population(%)
Compound Model-**3**	**3a**	0.00	42.0
	**3b**	0.20	29.8
	**3c**	0.56	16.3
	**3d**	0.75	11.9

S4. Quantum chemical CD calculation of compound **4**.

Conformational analysis was initially performed using Spartan'14 software (Wavefunction, Inc., Irvine, CA, USA) at MMFF94 force field. The conformers with Boltzmann-population of over 5% were chosen for ECD calculations, and then the conformers were initially optimized at B3LYP/DGTZVP level in MeOH using the intergral equation formalism polarizable continuum model (IEFPCM). Harmonic vibration frequencies were calculated to confirm the stability of these conformers. As revealed by the frequency analysis, no imaginary frequencies were observed in ground states. The theoretical calculation of ECD was conducted in MeOH using Time-dependent Density functional theory (TD-DFT) at the B3LYP/DGTZVP level for all conformers of compound **4**. The CD spectra were generated by the program GaussView 6.0 (University of Würzburg, Würzburg, Germany) using a Gaussian band shape with 0.3 eV exponential half-width from dipole-length dipolar and rotational strengths.

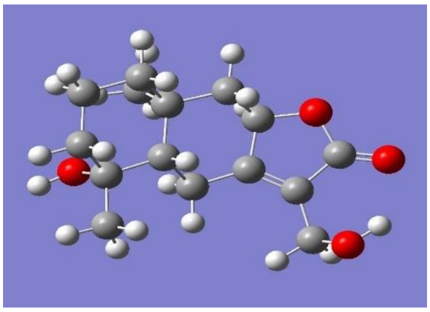

4a

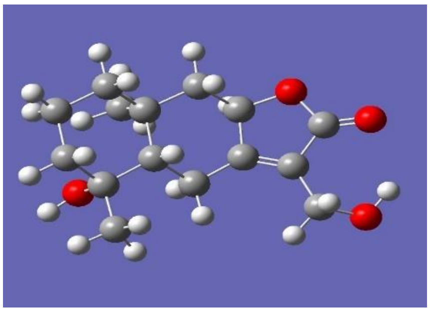

4b

Compound Model-**4**	Conformer	ΔE(kcal/mol)	Population(%)
	**4a**	0.00	57.7
	**4b**	0.18	42.3

S5. Quantum chemical CD calculation of compound **5**.

Conformational analysis was initially performed using Spartan'14 software (Wavefunction, Inc., Irvine, CA, USA) at MMFF94 force field. The conformers with Boltzmann-population of over 5% were chosen for ECD calculations, and then the conformers were initially optimized at B3LYP/6-311G level in MeOH using the intergral equation formalism polarizable continuum model (IEFPCM). Harmonic vibration frequencies were calculated to confirm the stability of these conformers. As revealed by the frequency analysis, no imaginary frequencies were observed in ground states. The theoretical calculation of ECD was conducted in MeOH using Time-dependent Density functional theory (TD-DFT) at the B3LYP/6-311G+ level for all conformers of compound **5**. The CD spectra were generated by the program GaussView 6.0 (University of Würzburg, Würzburg, Germany) using a Gaussian band shape with 0.3 eV exponential half-width from dipole-length dipolar and rotational strengths.

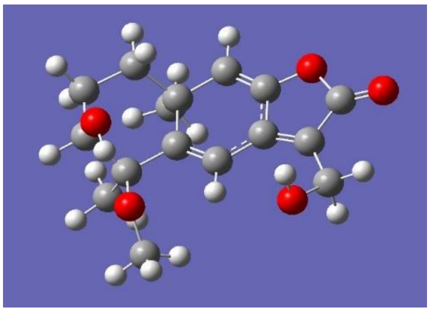

5a

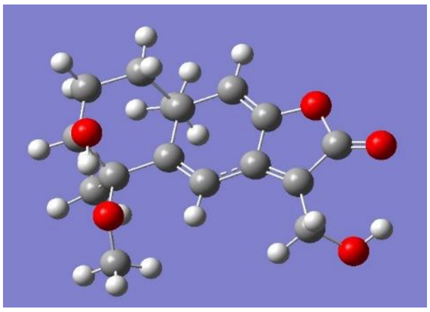

5b

	Conformer	ΔE(kcal/mol)	Population(%)
Compound Model-**5**	**5a**	0	51.5%
	**5b**	0.04	48.5%

S6. Quantum chemical CD calculation of compound **6**.

Conformational analysis was initially performed using Spartan’14 software (Wavefunction, Inc., Irvine, CA, USA) at MMFF94 force field. The conformers with Boltzmann-population of over 5% were chosen for ECD calculations, and then the conformers were initially optimized at B3LYP/6-311G level in MeOH using the intergral equation formalism polarizable continuum model (IEFPCM). Harmonic vibration frequencies were calculated to confirm the stability of these conformers. As revealed by the frequency analysis, no imaginary frequencies were observed in ground states. The theoretical calculation of ECD was conducted in MeOH using Time-dependent Density functional theory (TD-DFT) at the B3LYP/6-311G+ level for all conformers of compound **6**. The CD spectra were generated by the program GaussView 6.0 (University of Würzburg, Würzburg, Germany) using a Gaussian band shape with 0.3 eV exponential half-width from dipole-length dipolar and rotational strengths.

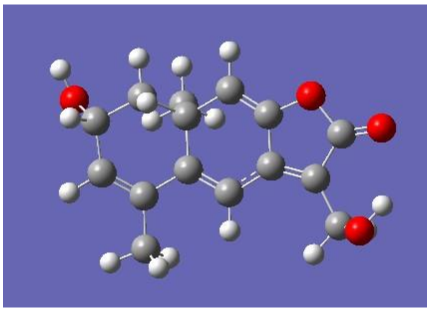

6a

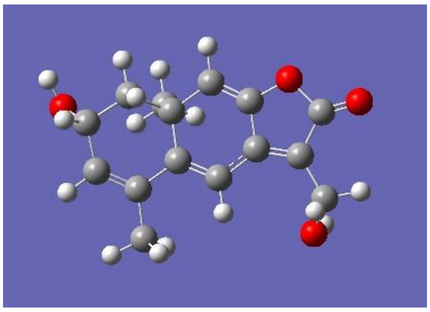

6b

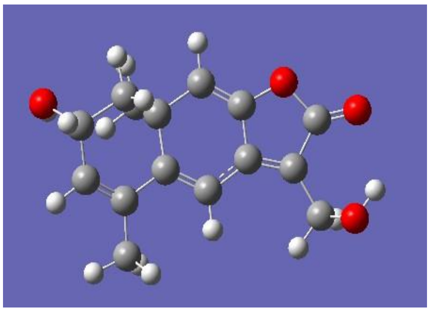

6c

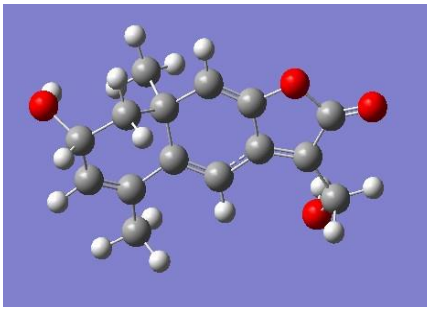

6d

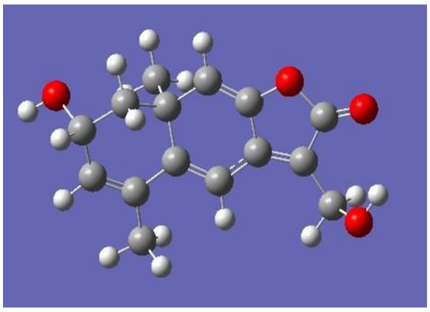

6e

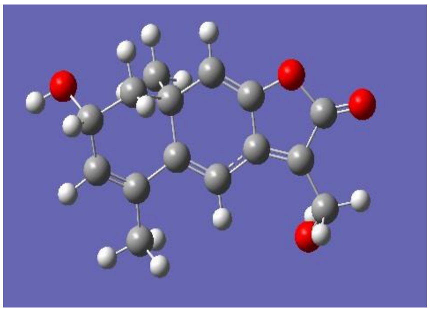

6f

	Conformer	ΔE(kcal/mol)	Population(%)
Compound Model-**6**	**6a**	0	29.0
	**6b**	0.13	23.4
	**6c**	0.24	19.2
	**6d**	0.38	15.2
	**6e**	0.84	7.0
	**6f**	0.92	6.2

S7. Quantum chemical CD calculation of compound **7**.

Conformational analysis was initially performed using Spartan'14 software (Wavefunction, Inc., Irvine, CA, USA) at MMFF94 force field. The conformers with Boltzmann-population of over 5% were chosen for ECD calculations, and then the conformers were initially optimized at B3LYP/DGTZVP level in MeOH using the intergral equation formalism polarizable continuum model (IEFPCM). Harmonic vibration frequencies were calculated to confirm the stability of these conformers. As revealed by the frequency analysis, no imaginary frequencies were observed in ground states. The theoretical calculation of ECD was conducted in MeOH using Time-dependent Density functional theory (TD-DFT) at the B3LYP/DGTZVP level for all conformers of compound **7**. The CD spectra were generated by the program GaussView 6.0 (University of Würzburg, Würzburg, Germany) using a Gaussian band shape with 0.3 eV exponential half-width from dipole-length dipolar and rotational strengths.

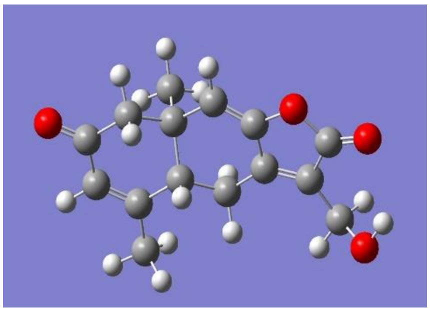

7a

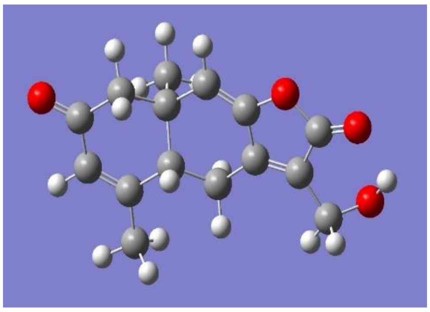

7b

	Conformer	ΔE(kcal/mol)	Population(%)
Compound Model-**7**	**7a**	0.00	57.7
	**7b**	0.18	42.3

S8. Quantum chemical CD calculation of compound **8**.

Conformational analysis was initially performed using Spartan'14 software (Wavefunction, Inc., Irvine, CA, USA) at MMFF94 force field. The conformers with Boltzmann-population of over 5% were chosen for ECD calculations, and then the conformers were initially optimized at B3LYP/DGTZVP level in MeOH using the intergral equation formalism polarizable continuum model (IEFPCM). Harmonic vibration frequencies were calculated to confirm the stability of these conformers. As revealed by the frequency analysis, no imaginary frequencies were observed in ground states. The theoretical calculation of ECD was conducted in MeOH using Time-dependent Density functional theory (TD-DFT) at the B3LYP/DGTZVP level for all conformers of compound **8**. The CD spectra were generated by the program GaussView 6.0 (University of Würzburg, Würzburg, Germany) using a Gaussian band shape with 0.3 eV exponential half-width from dipole-length dipolar and rotational strengths.

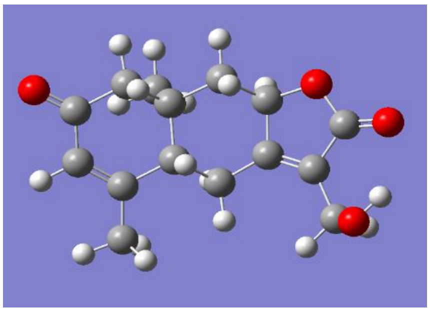

8a

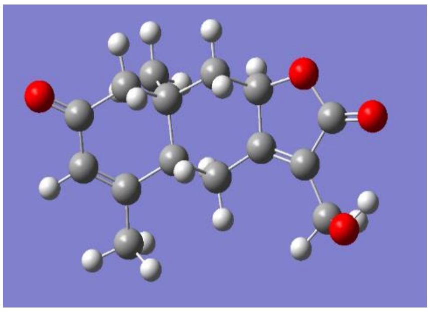

8b

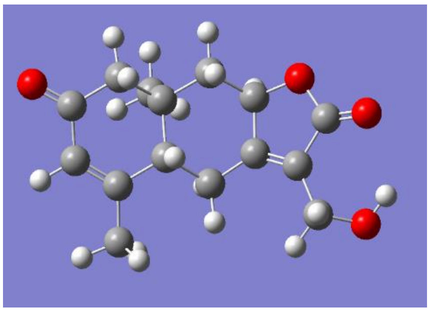

8c

	Conformer	ΔE(kcal/mol)	Population(%)
Compound Model-**8**	**8a**	0.00	35.8
	**8b**	0.00	35.7
	**8c**	0.13	28.5

S9. Quantum chemical CD calculation of compound **10**.

Conformational analysis was initially performed using Spartan'14 software (Wavefunction, Inc., Irvine, CA, USA) at MMFF94 force field. The conformers with Boltzmann-population of over 5% were chosen for ECD calculations, and then the conformers were initially optimized at B3LYP/6-311G level in MeOH using the intergral equation formalism polarizable continuum model (IEFPCM). Harmonic vibration frequencies were calculated to confirm the stability of these conformers. As revealed by the frequency analysis, no imaginary frequencies were observed in ground states. The theoretical calculation of ECD was conducted in MeOH using Time-dependent Density functional theory (TD-DFT) at the B3LYP/6-311G+ level for all conformers of compound **10**. The CD spectra were generated by the program GaussView 6.0 (University of Würzburg, Würzburg, Germany) using a Gaussian band shape with 0.3 eV exponential half-width from dipole-length dipolar and rotational strengths.

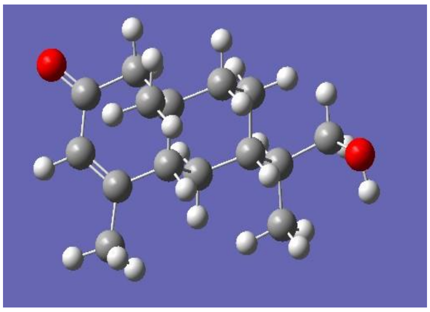

10a

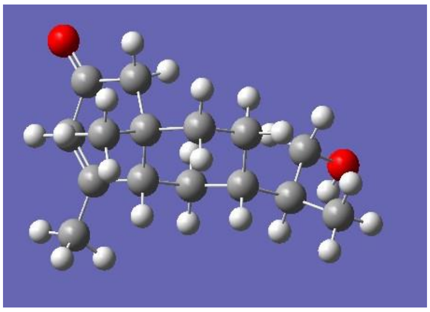

10b

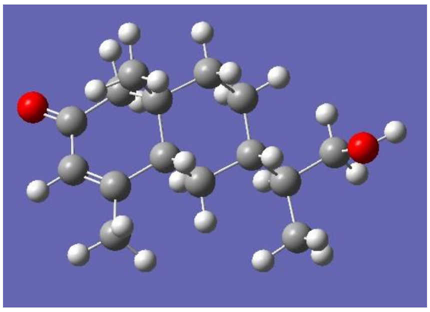

10c

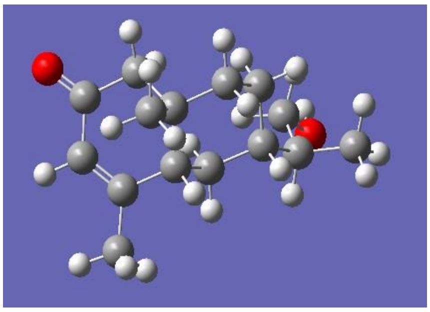

10d

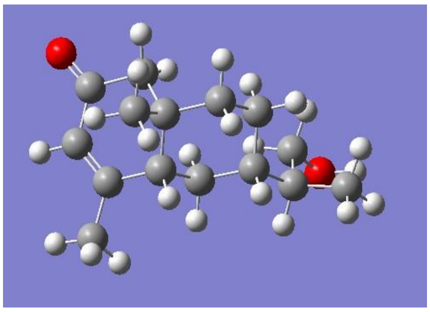

10e

	Conformer	ΔE(kcal/mol)	Population(%)
Compound Model-**10**	**10a**	0	25.4
	**10b**	0.08	22.2
	**10c**	0.18	18.8
	**10d**	0.20	18.1
	**10e**	0.29	15.5

S10. ^{1}H NMR spectrum of Eutypellaolide A (**1**) in $CDCl_3$

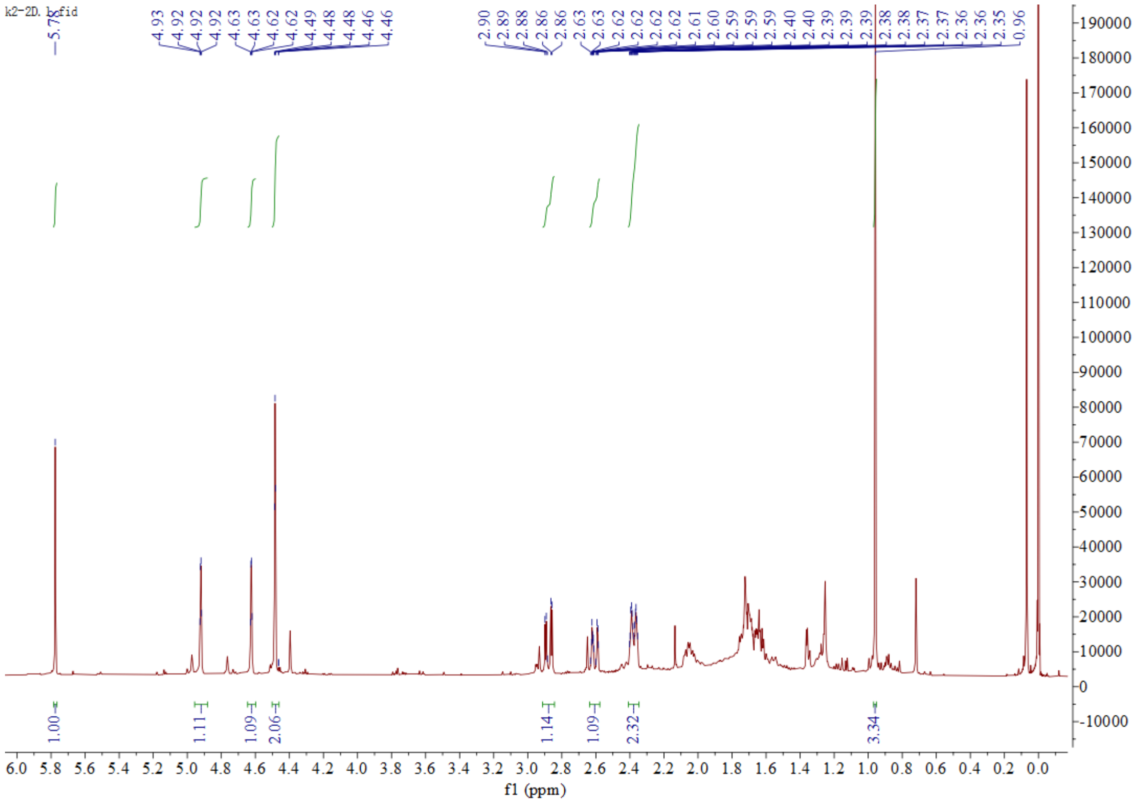

S11. ^{13}C NMR spectrum of Eutypellaolide A (**1**) in $CDCl_3$

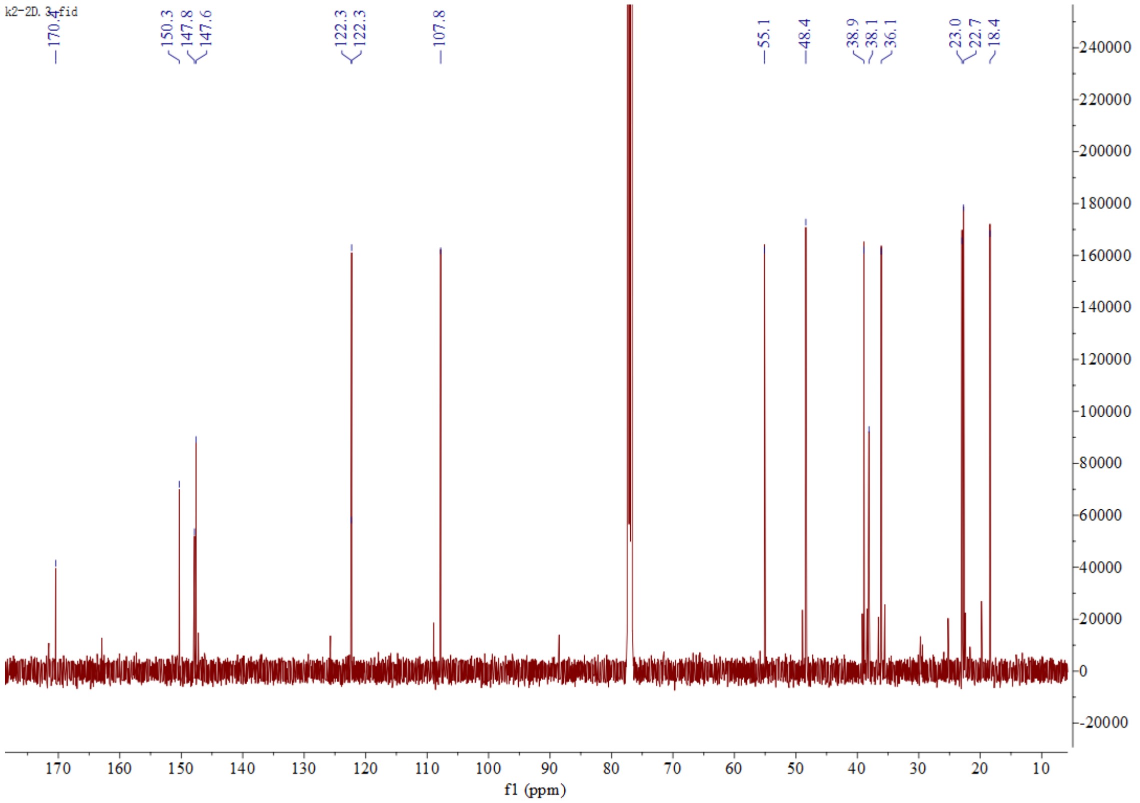

S12. DEPT135 spectrum of Eutypellaolide A (**1**) in $CDCl_3$

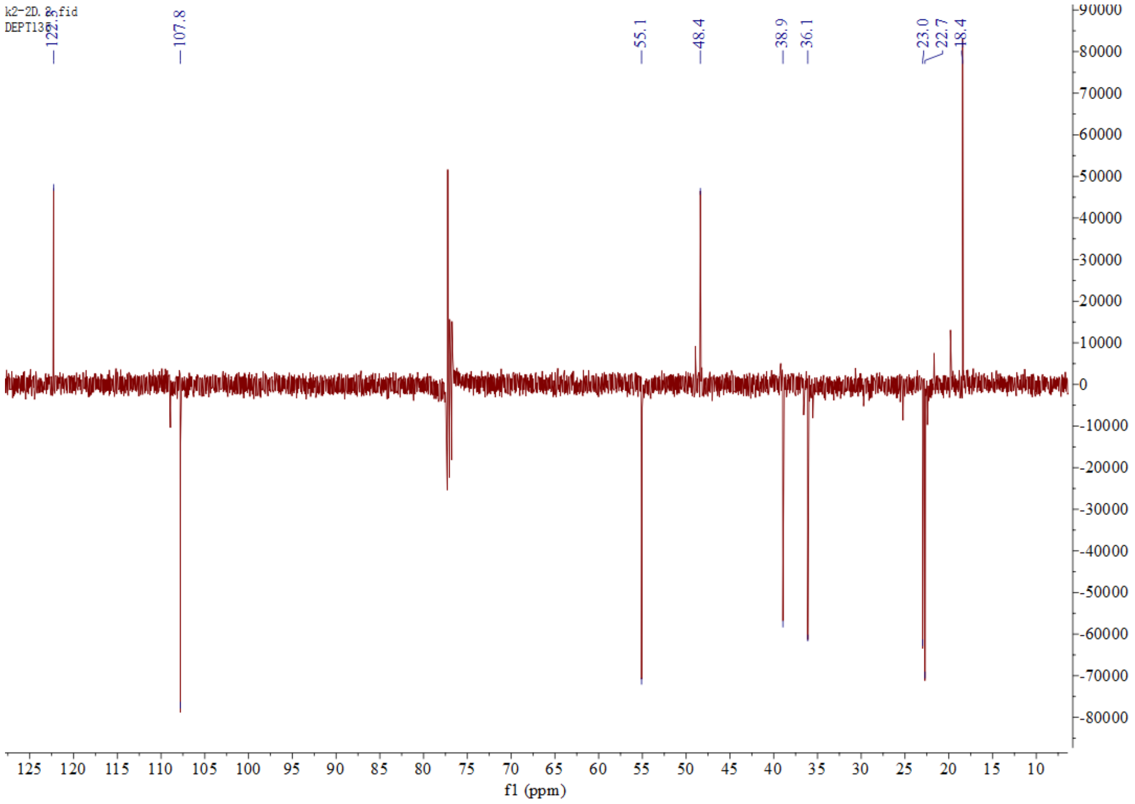

S13. HSQC spectrum of Eutypellaolide A (**1**) in $CDCl_3$

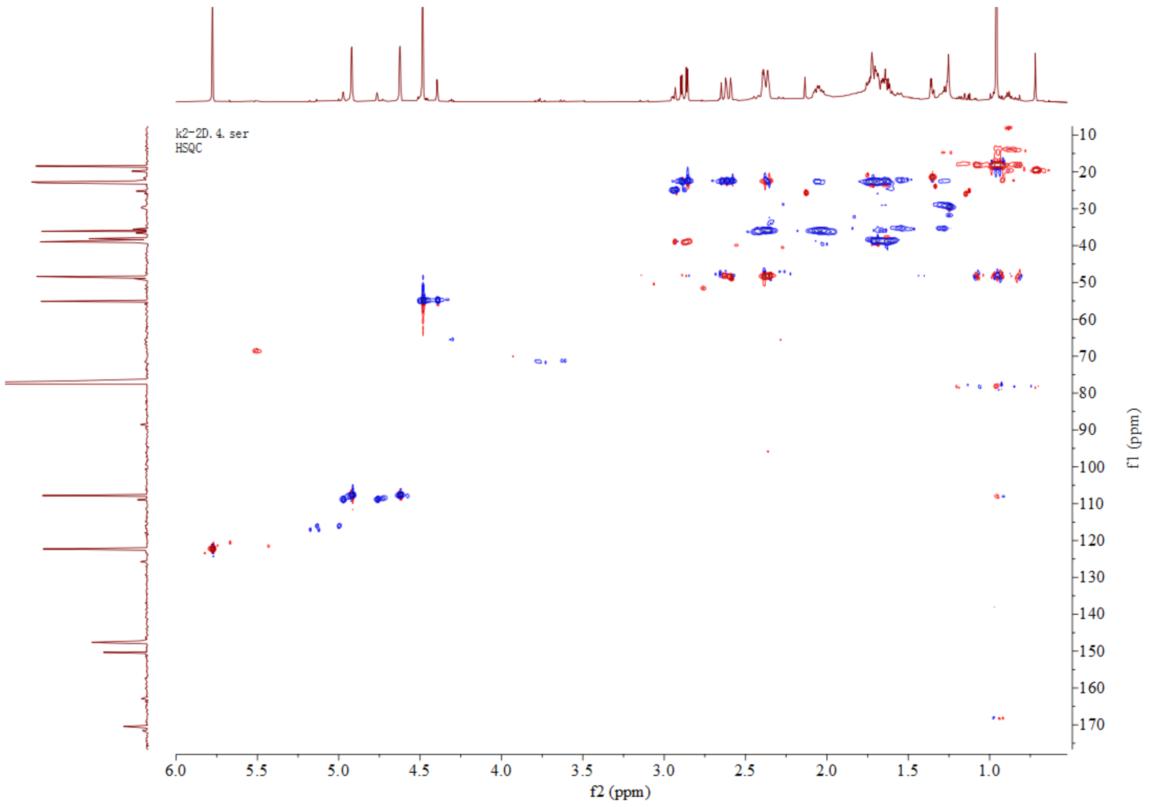

S14. COSY spectrum of Eutypellaolide A (**1**) in $CDCl_3$

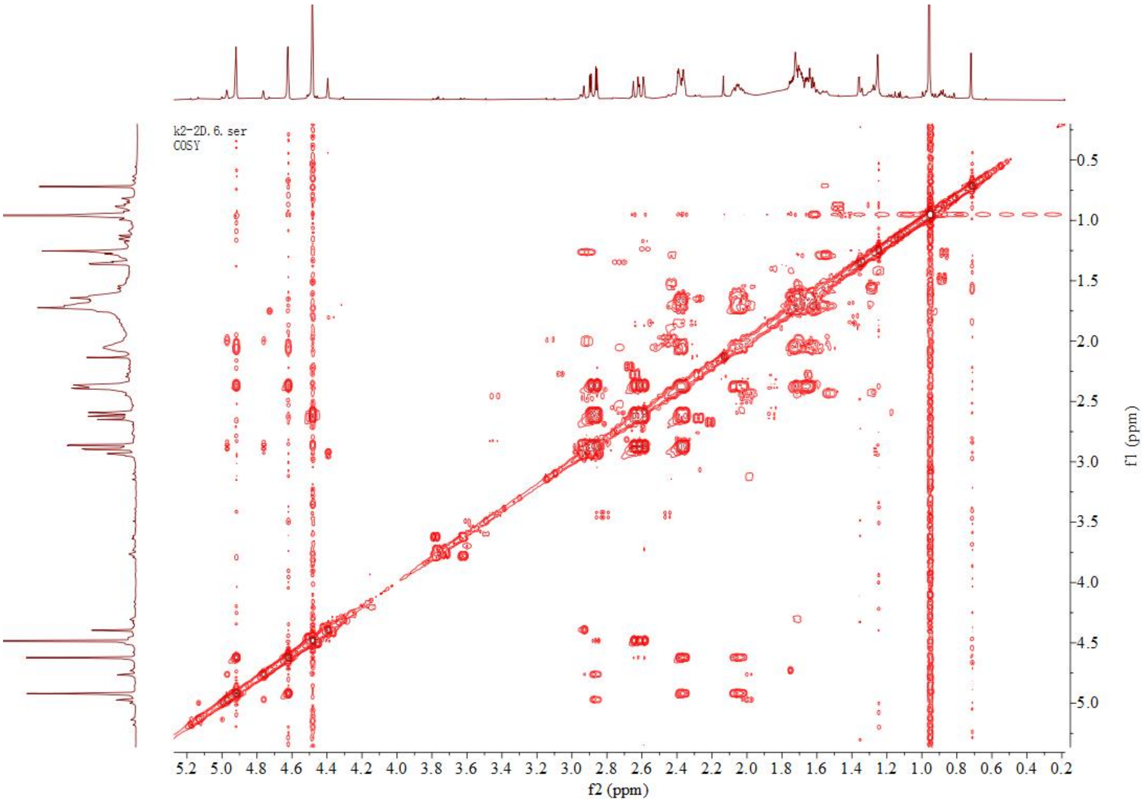

S15. HMBC spectrum of Eutypellaolide A (**1**) in $CDCl_3$

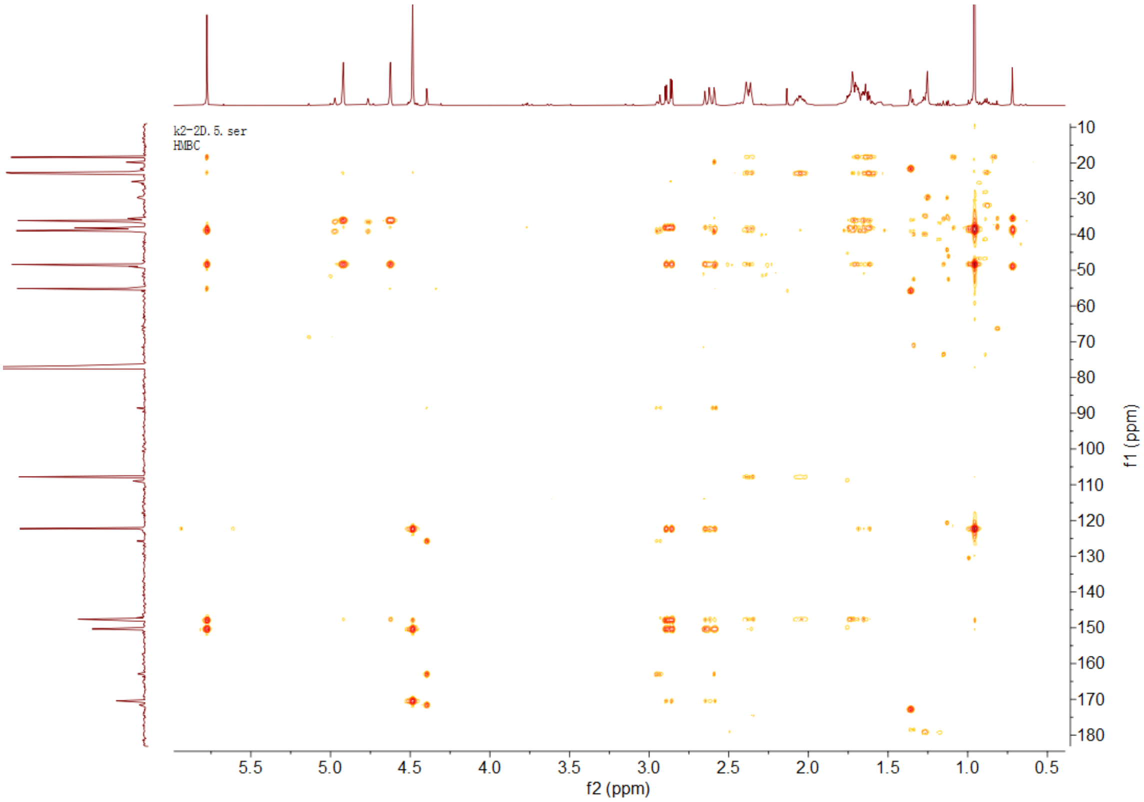

S16. NOESY spectrum of Eutypellaolide A (**1**) in $CDCl_3$

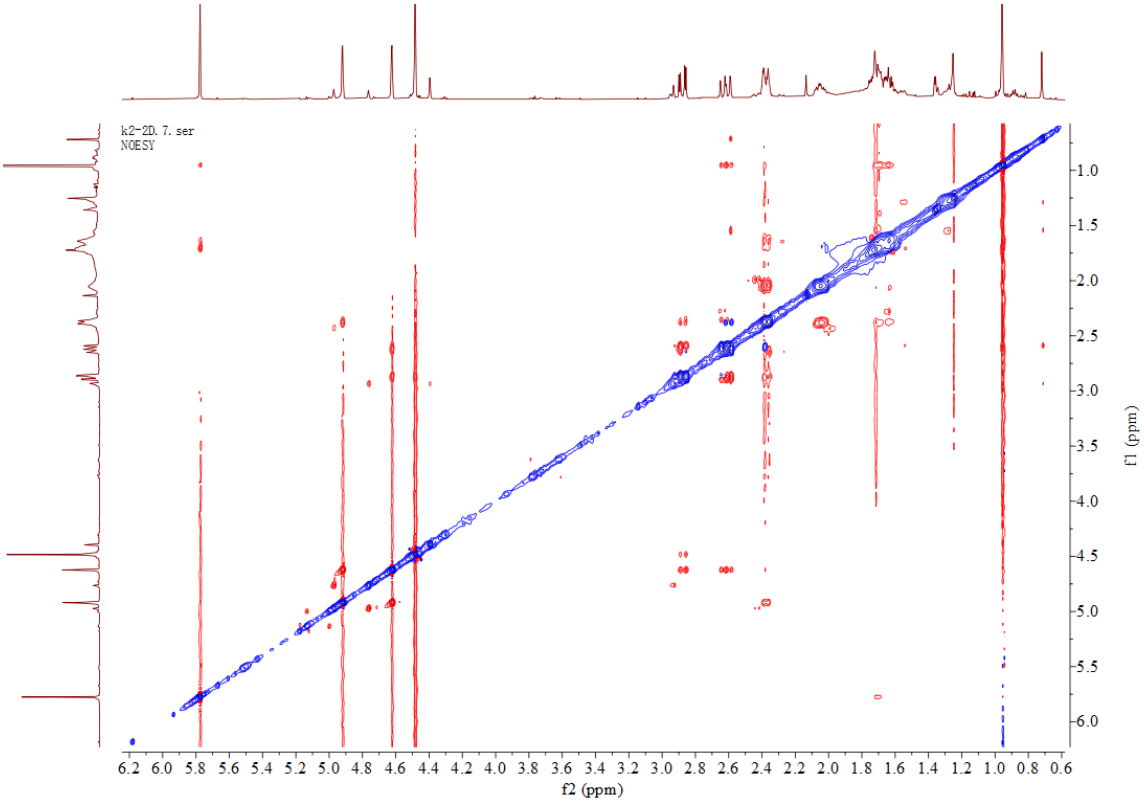

S17. HRESIMS of Eutypellaolide A (**1**)

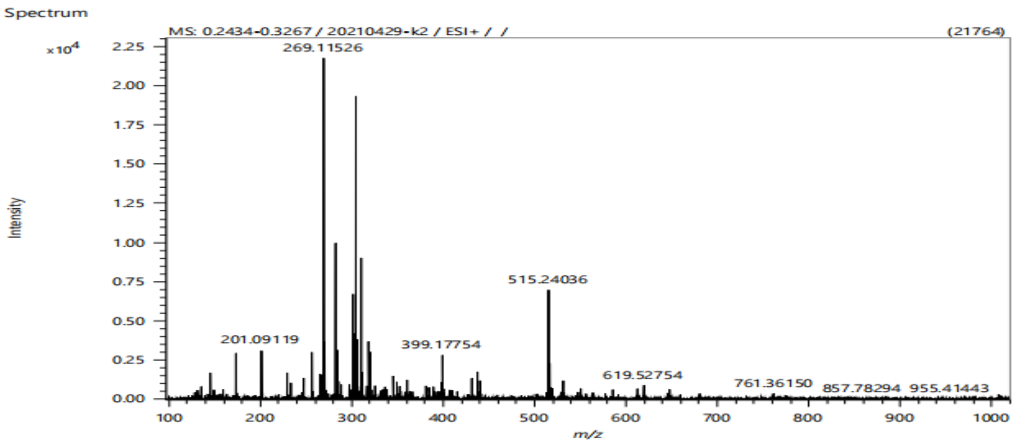

Elemental Composition

Parameters

Tolerance:	±5.00 ppm
Electron:	Odd/Even
Charge:	+1
DBE:	-1.5 - 200.0

Elements Set 1:

Symbol	C	H	N	O	Na	S	Cl	Br
Min	0	0	0	0	1	0	0	0
Max	200	200	0	8	1	0	0	0

Symbol	F	P	Si
Min	0	0	0
Max	0	0	0

Results

Mass	Intensity	Intensity [%]	Formula	Calculated Mass	Mass Difference [mDa]	Mass Difference [ppm]	DBE
269.11526	21764.11	100.00	C15 H18 O3 Na	269.11482	0.44	1.65	6.5

S18. UV spectrum of Eutypellaolide A (**1**) in MeOH

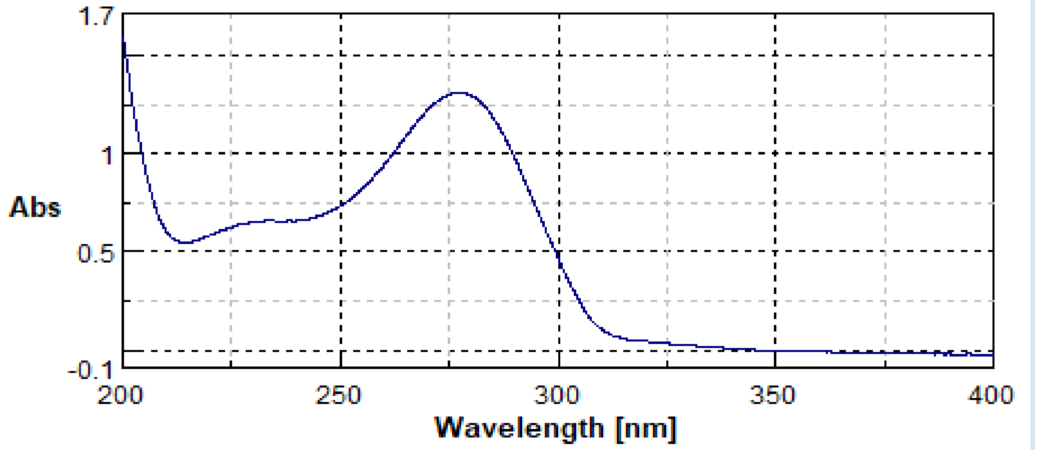

S19. IR spectrum of Eutypellaolide A (**1**)

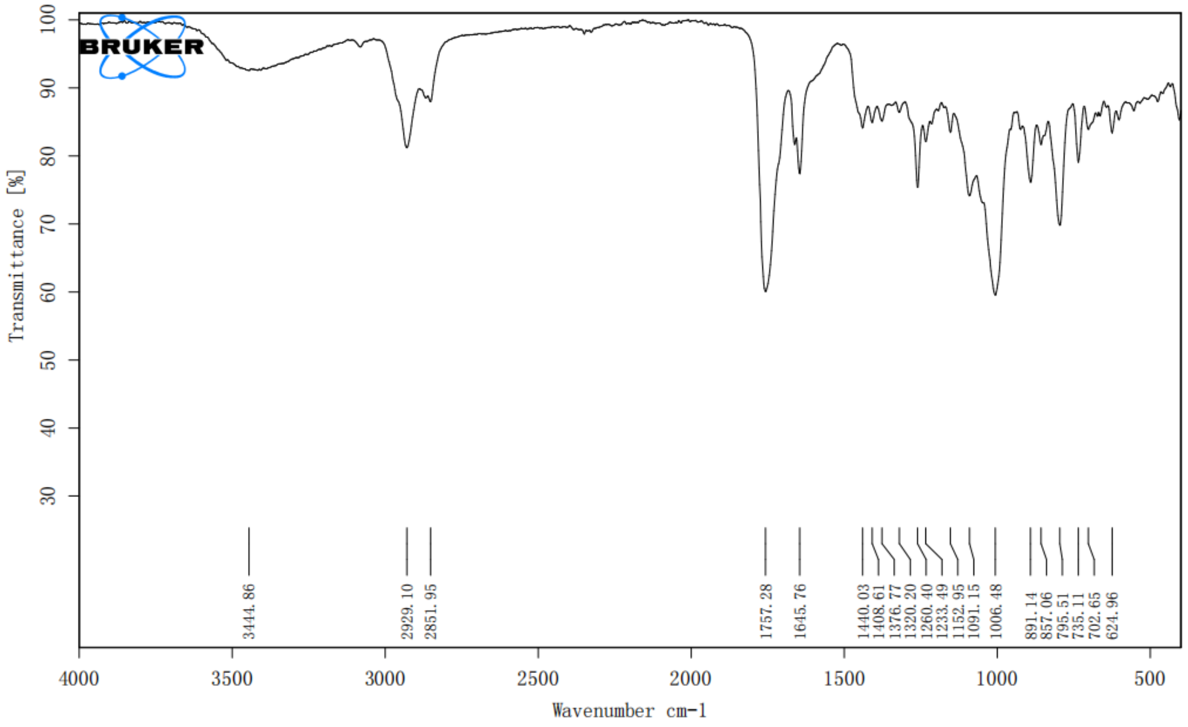

S20. ^{1}H NMR spectrum of Eutypellaolide B (**2**) in DMSO-*d6*

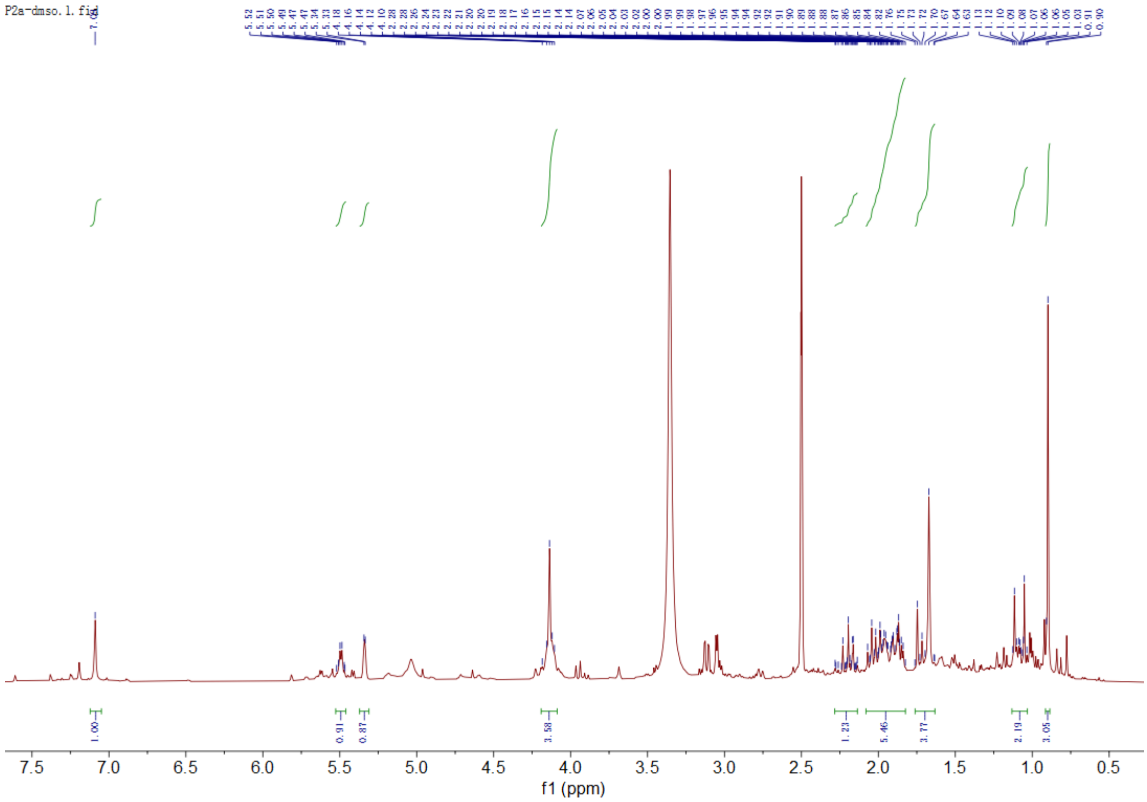

S21. ^{13}C NMR spectrum of Eutypellaolide B (**2**) in DMSO-*d6*

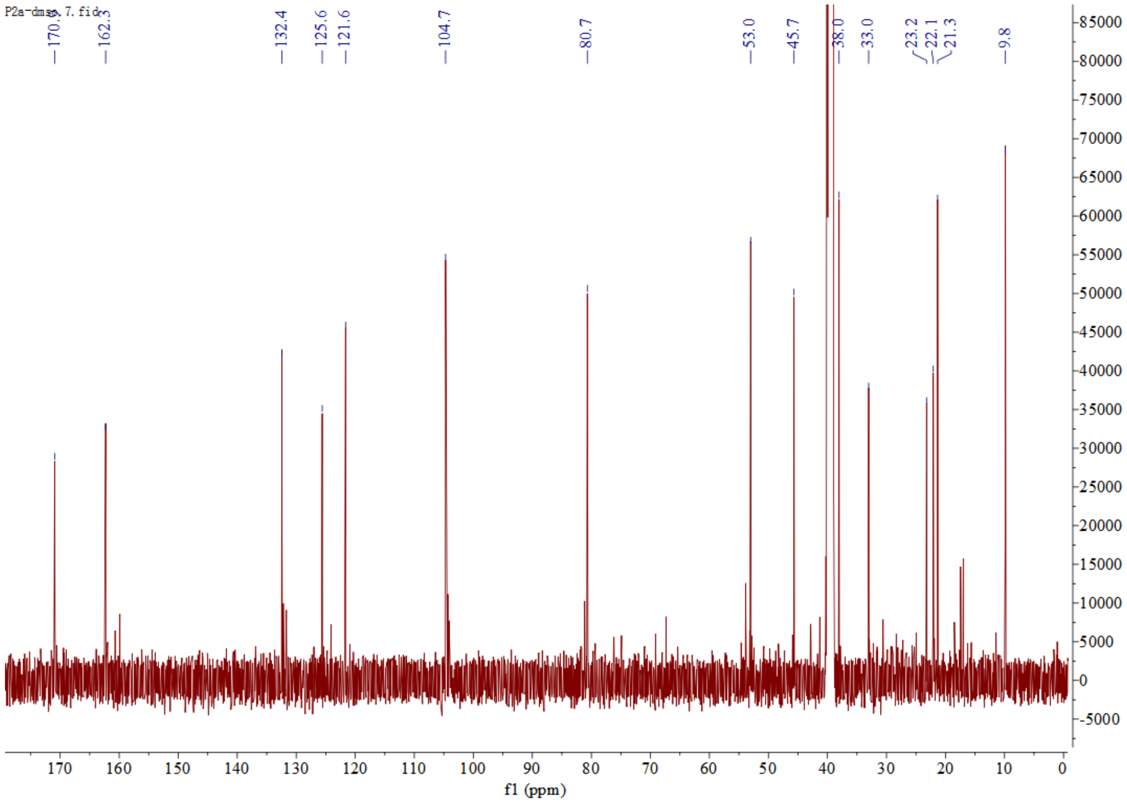

S22. DEPT135 spectrum of Eutypellaolide B (**2**) in DMSO-*d6*

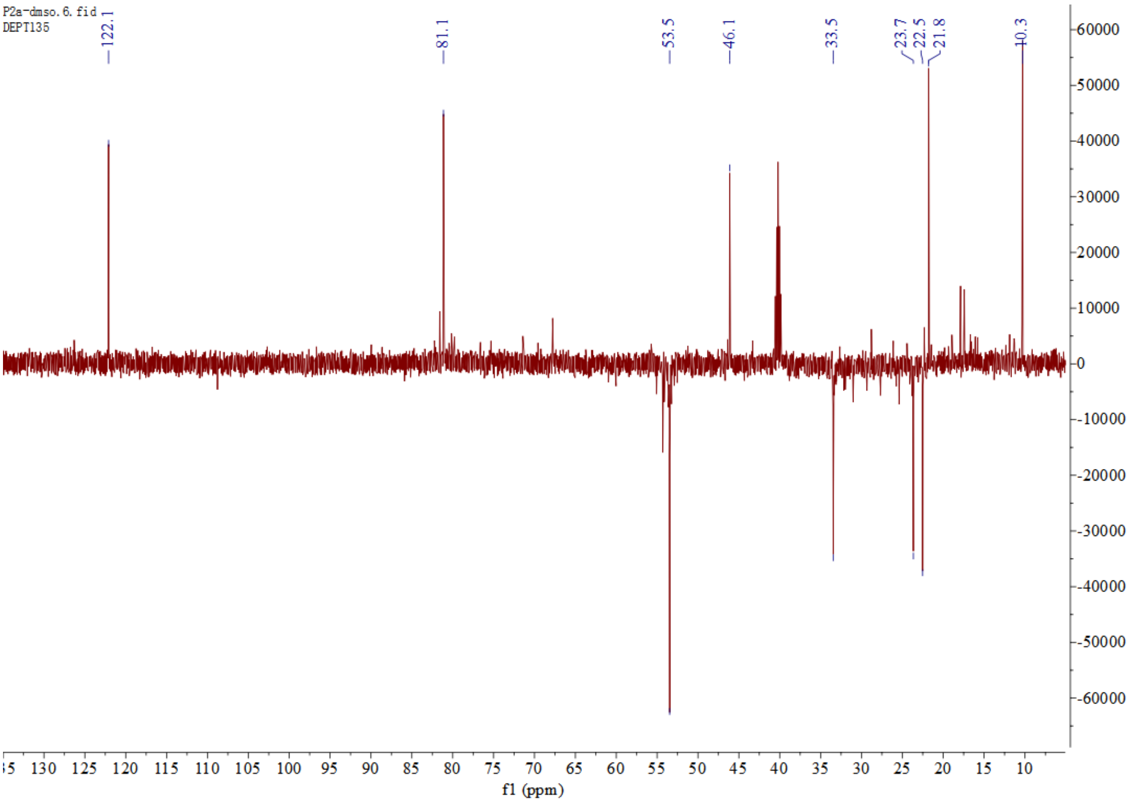

S23. HSQC spectrum of Eutypellaolide B (**2**) in DMSO-*d6*

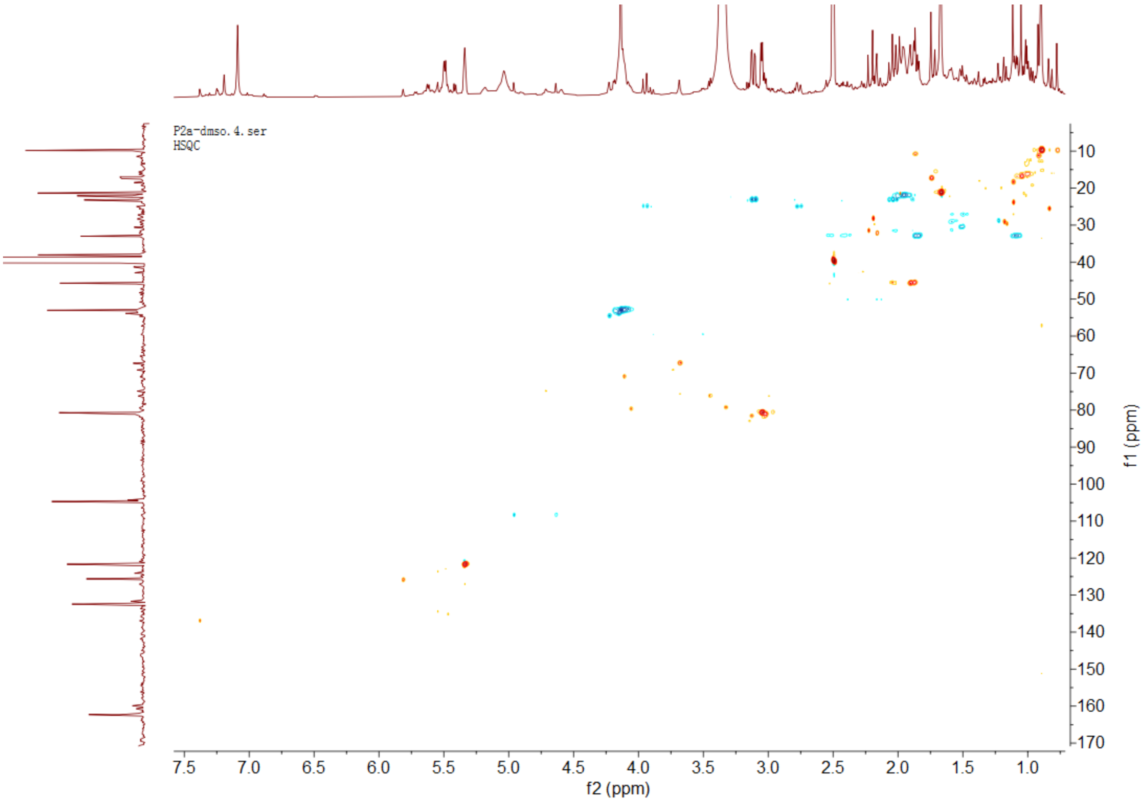

S24. COSY spectrum of Eutypellaolide B (**2**) in DMSO-*d6*

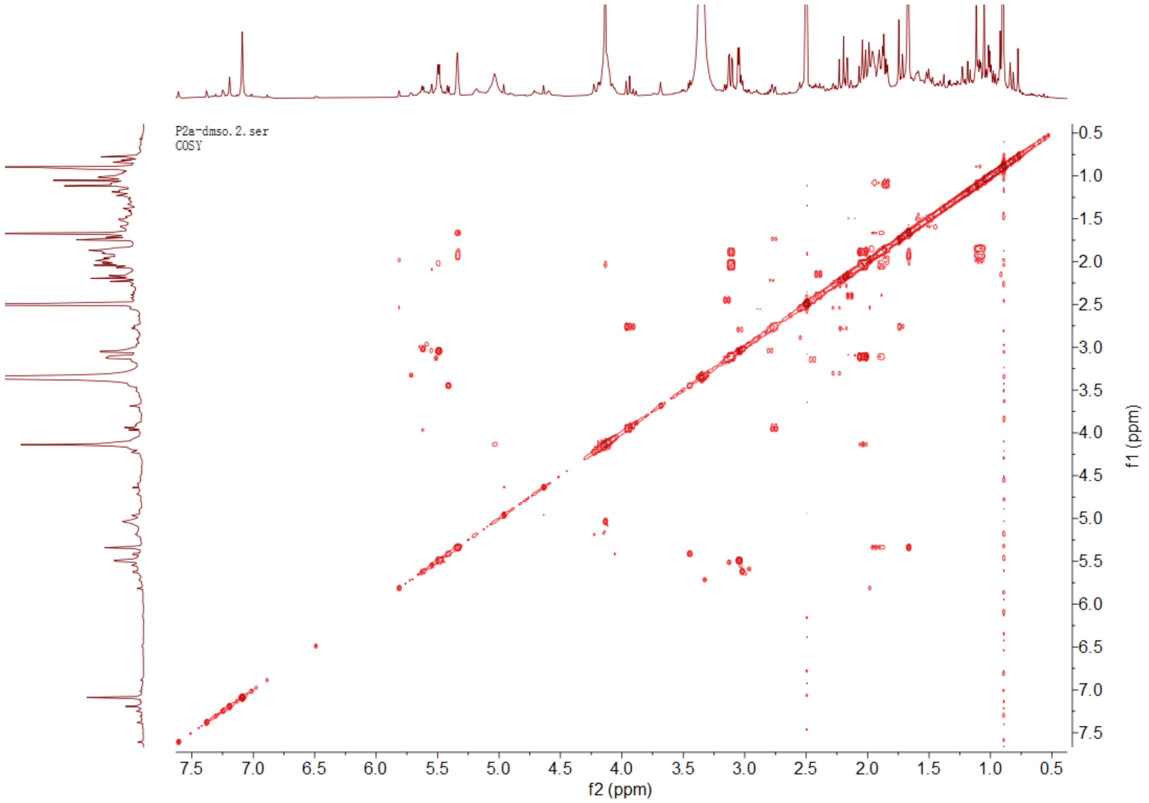

S25. HMBC spectrum of Eutypellaolide B (**2**) in DMSO-*d6*

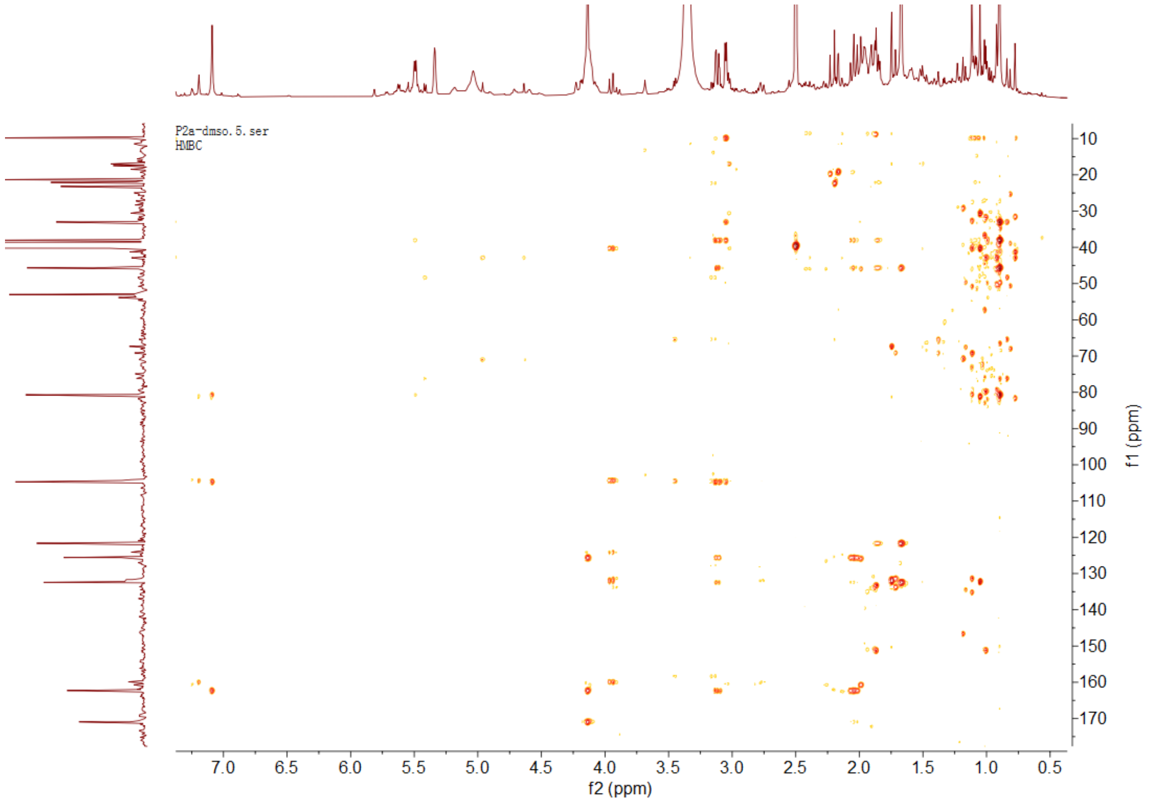

S26. NOESY spectrum of Eutypellaolide B (**2**) in DMSO-*d6*

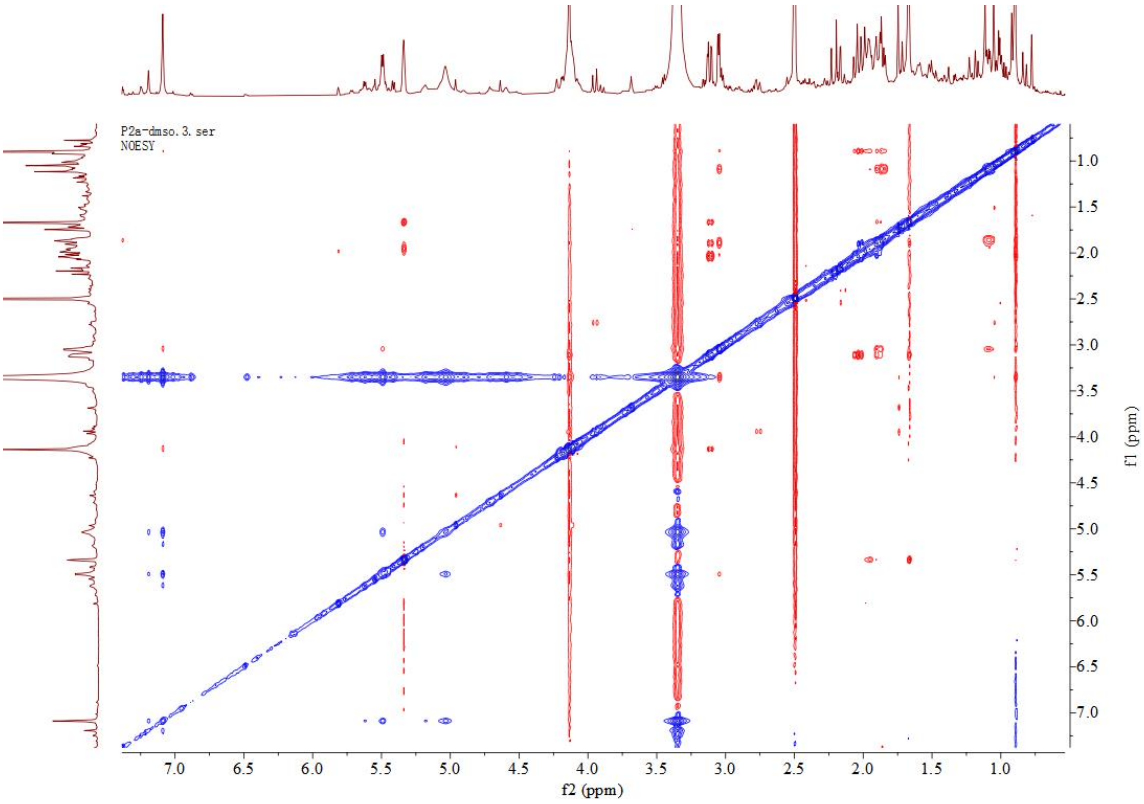

S27. HRESIMS of Eutypellaolide B (**2**)

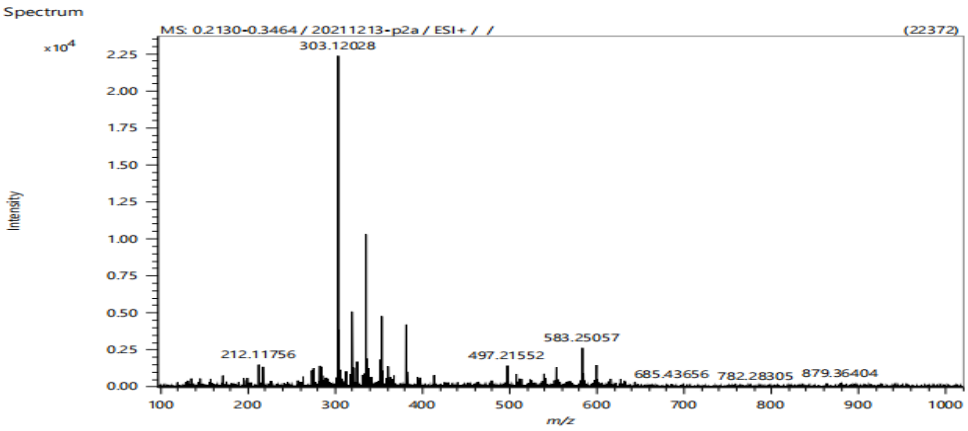

Elemental Composition

Parameters

Tolerance:	±5.00 ppm
Electron:	Odd/Even
Charge:	+1
DBE:	-1.5 - 200.0

Elements Set 1:

Symbol	C	H	N	O	Na	S	Cl	Br
Min	0	0	0	0	1	0	0	0
Max	200	200	3	8	1	0	0	0

Symbol	P	F
Min	0	0
Max	0	0

Results

Mass	Intensity	Intensity [%]	Formula	Calculated Mass	Mass Difference [mDa]	Mass Difference [ppm]	DBE
303.12028	22372.15	100.00	C15 H20 O5 Na	303.12029	-0.02	-0.06	5.5
			C13 H18 N3 O4 Na	303.11895	1.33	4.37	6.0

S28. UV spectrum of Eutypellaolide B (**2**) in MeOH

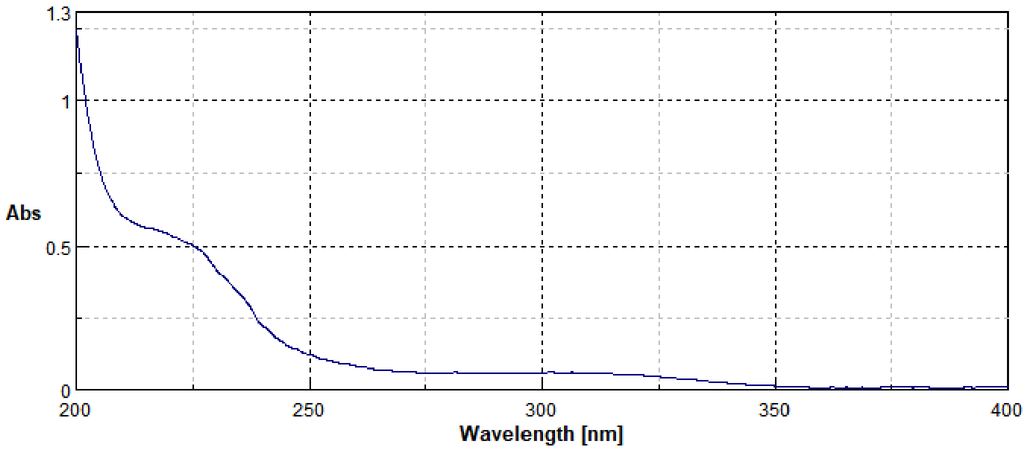

S29. IR spectrum of Eutypellaolide B (**2**)

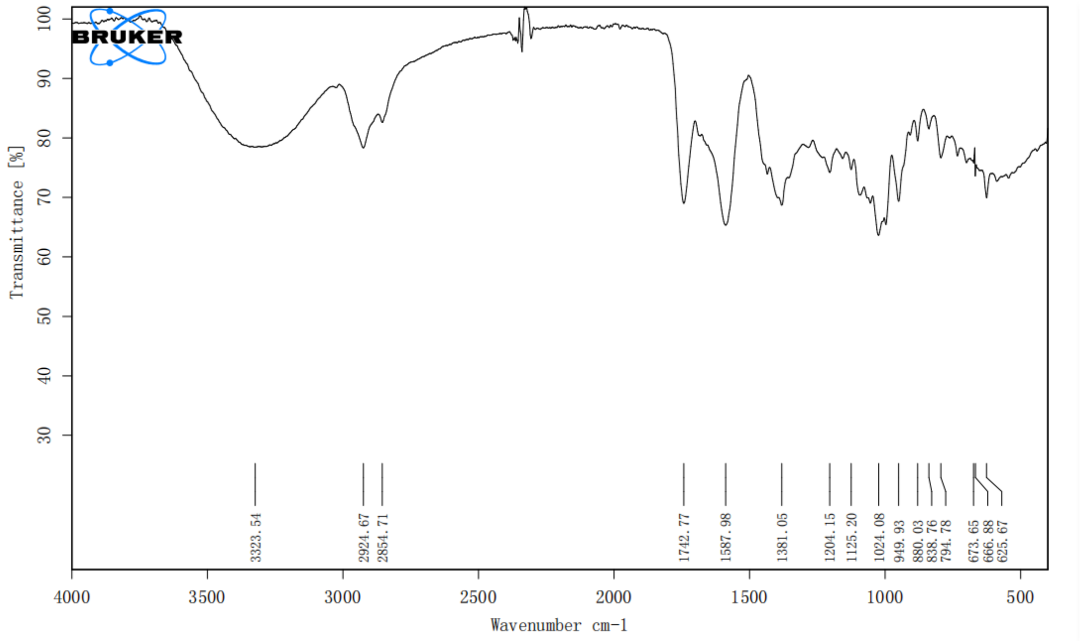

S30. ^{1}H NMR spectrum of Eutypellaolide C (**3**) in $CDCl_3$

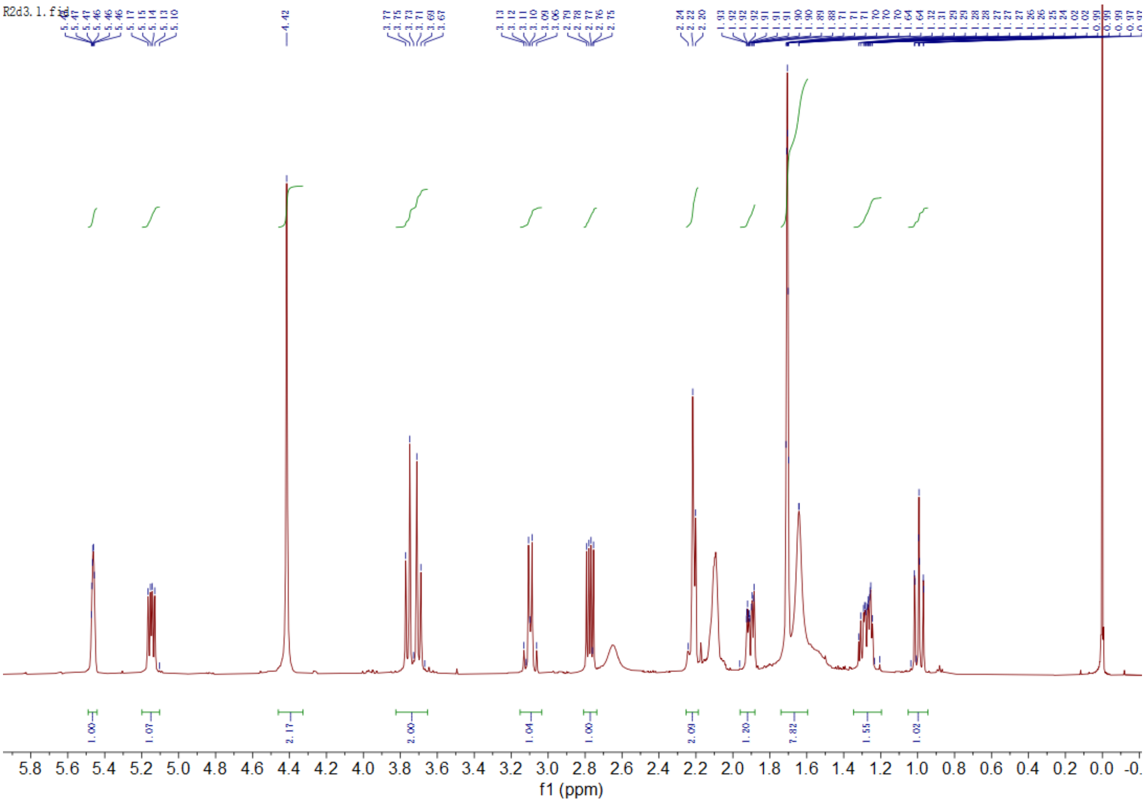

S31. ^{13}C NMR spectrum of Eutypellaolide C (**3**) in $CDCl_3$

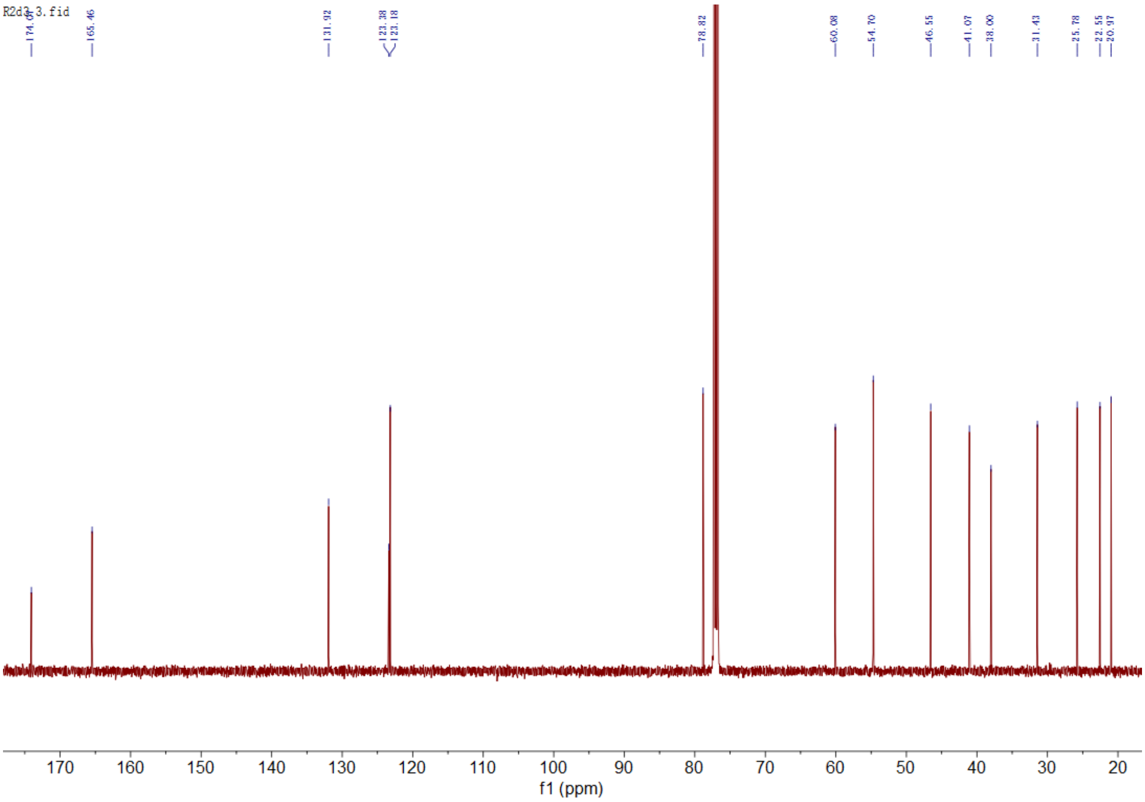

S32. DEPT135 spectrum of Eutypellaolide C (**3**) in $CDCl_3$

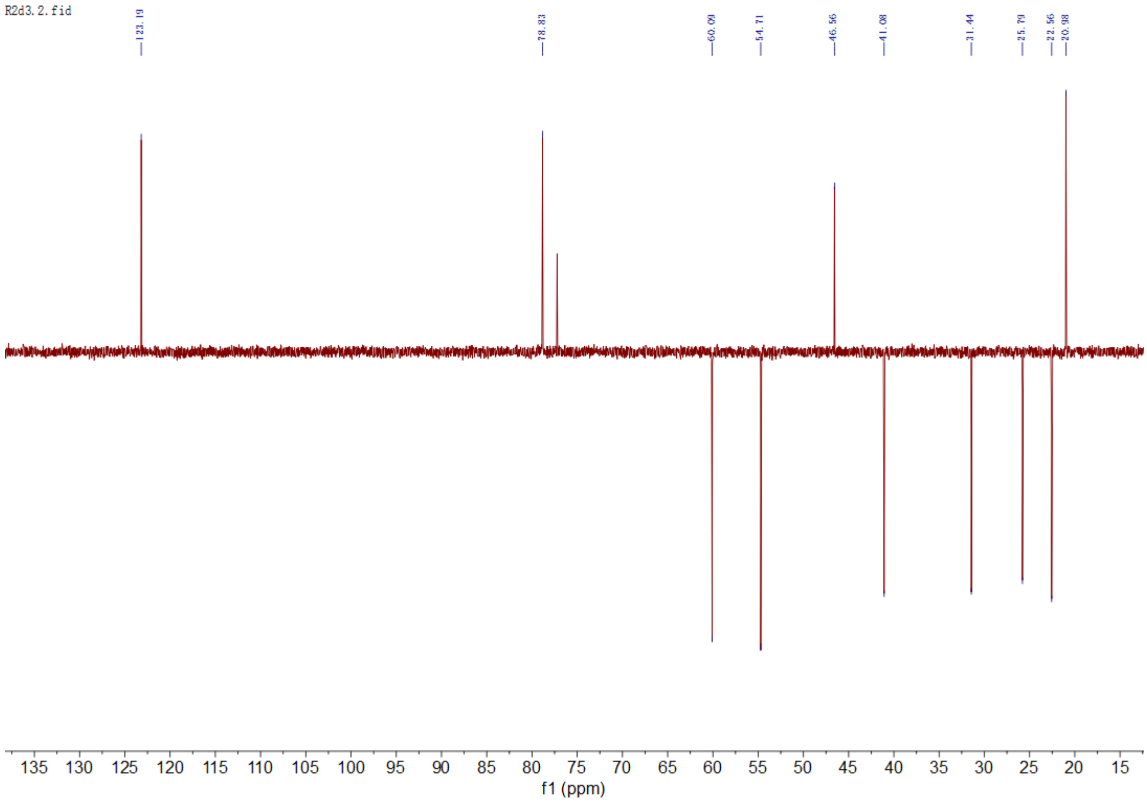

S33. HSQC spectrum of Eutypellaolide C (**3**) in $CDCl_3$

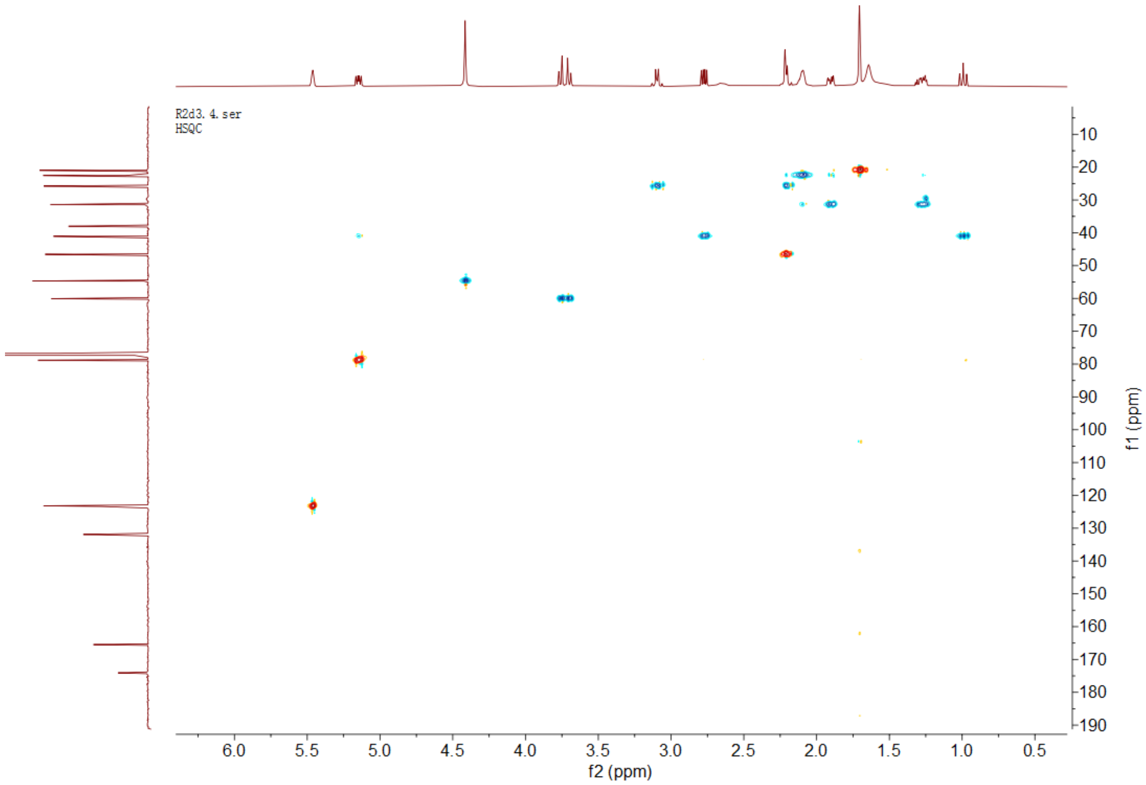

S34. COSY spectrum of Eutypellaolide C (**3**) in $CDCl_3$

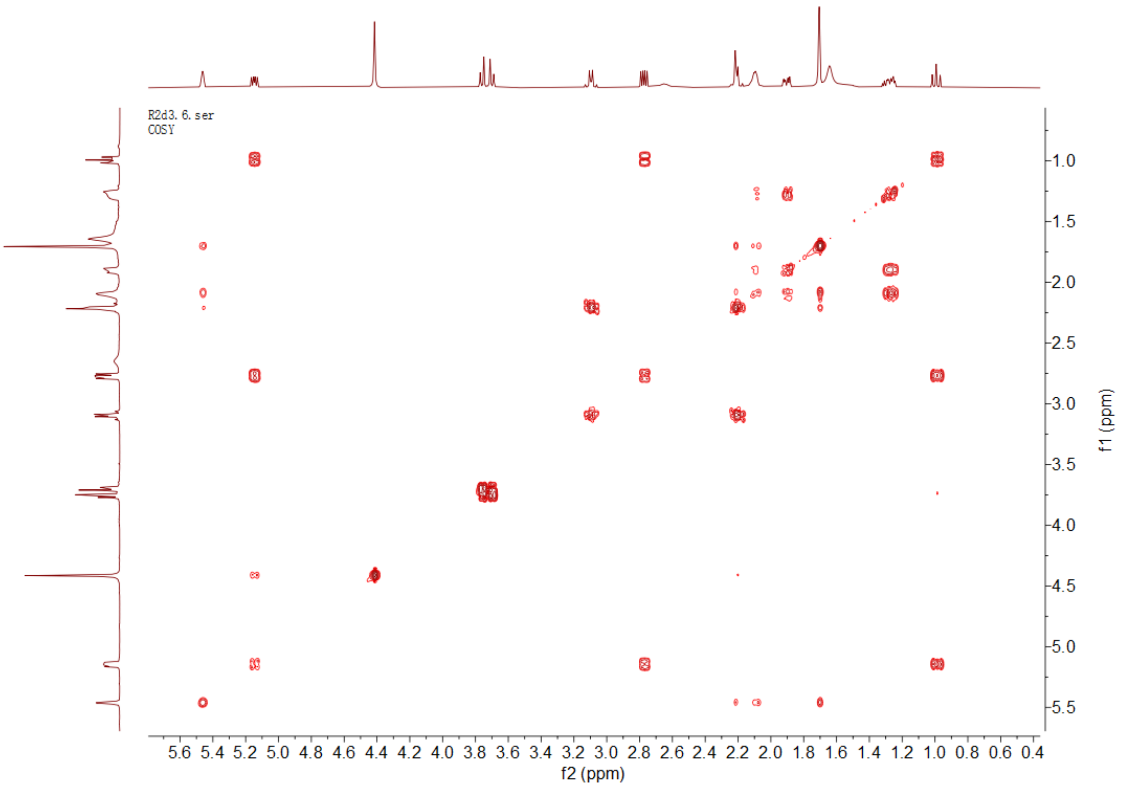

S35. HMBC spectrum of Eutypellaolide C (**3**) in $CDCl_3$

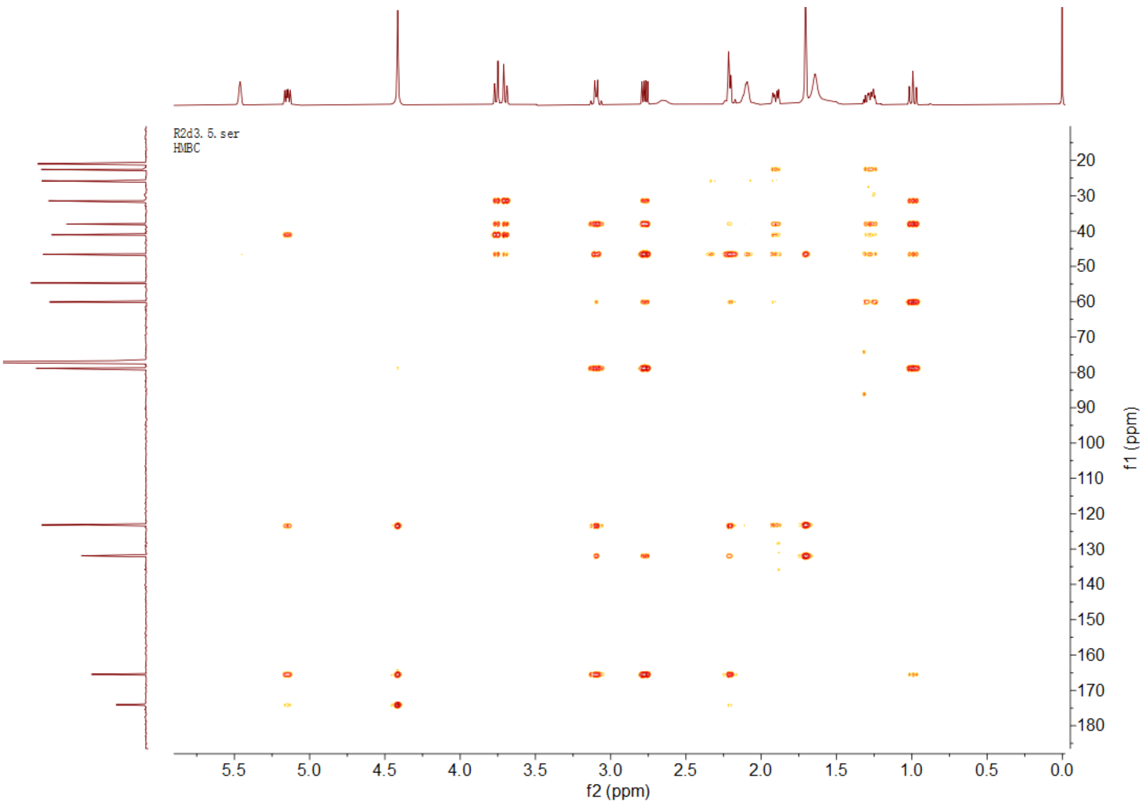

S36. NOESY spectrum of Eutypellaolide C (**3**) in $CDCl_3$

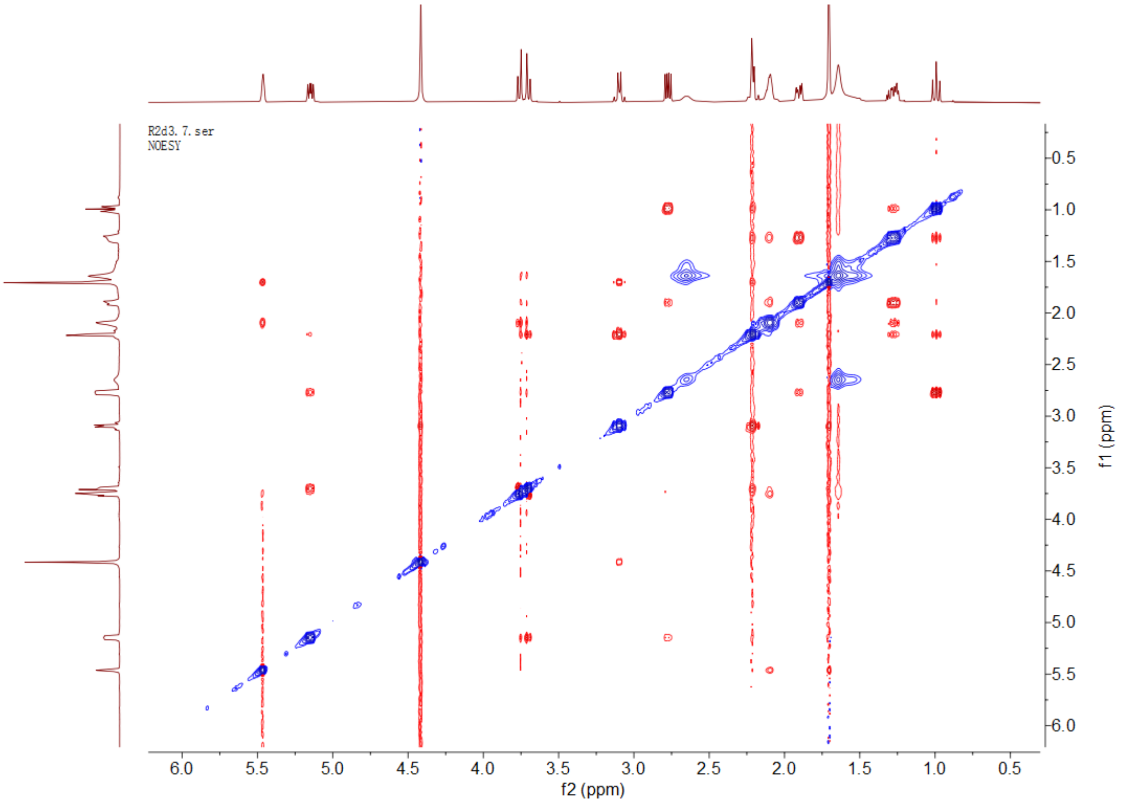

S37. HRESIMS of Eutypellaolide C (**3**)

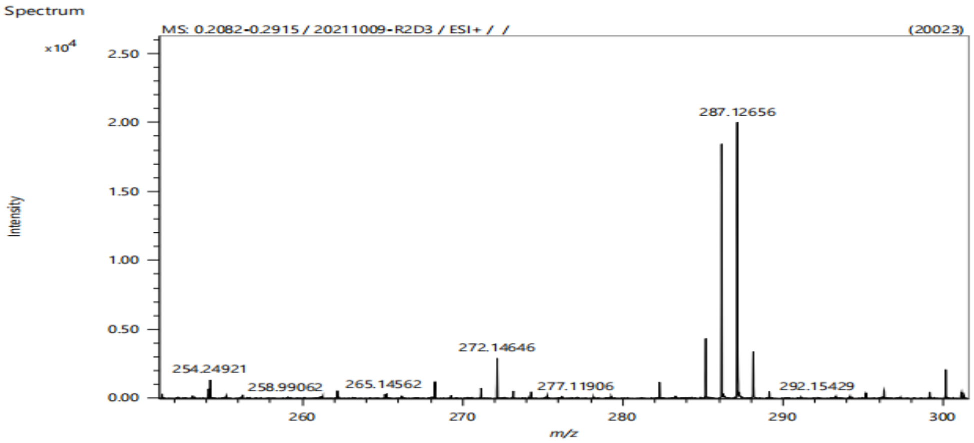

Elemental Composition

Parameters

Tolerance:	±20.00 ppm
Electron:	Odd/Even
Charge:	+1
DBE:	-1.5 - 200.0

Elements Set 1:

Symbol	C	H	N	O	Na	S	Cl	Br
Min	0	0	0	0	1	0	0	0
Max	200	200	0	8	1	0	0	0

Symbol	P	Si	F
Min	0	0	0
Max	0	0	0

Results

Mass	Intensity	Intensity [%]	Formula	Calculated Mass	Mass Difference [mDa]	Mass Difference [ppm]	DBE
287.12656	20022.76	18.37	C15 H20 O4 Na	287.12538	1.18	4.12	5.5

S38. UV spectrum of Eutypellaolide C (**3**) in MeOH

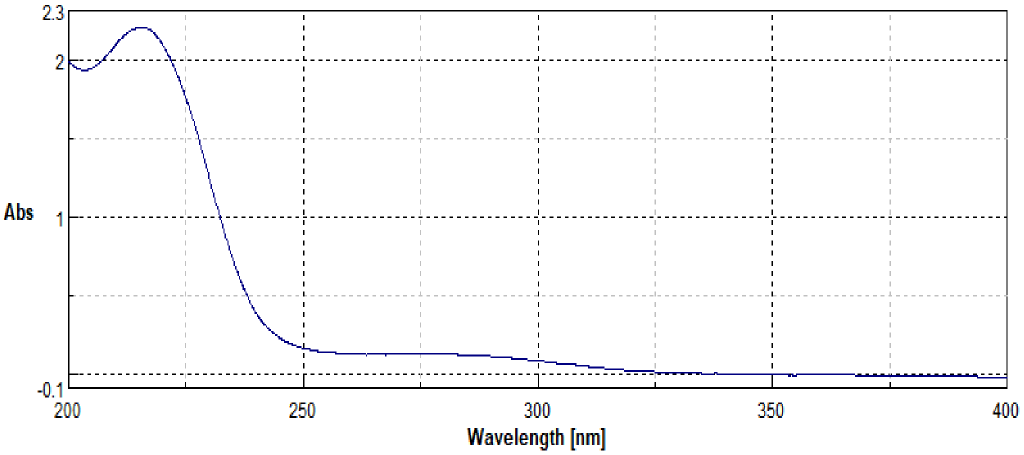

S39. IR spectrum of Eutypellaolide C (**3**)

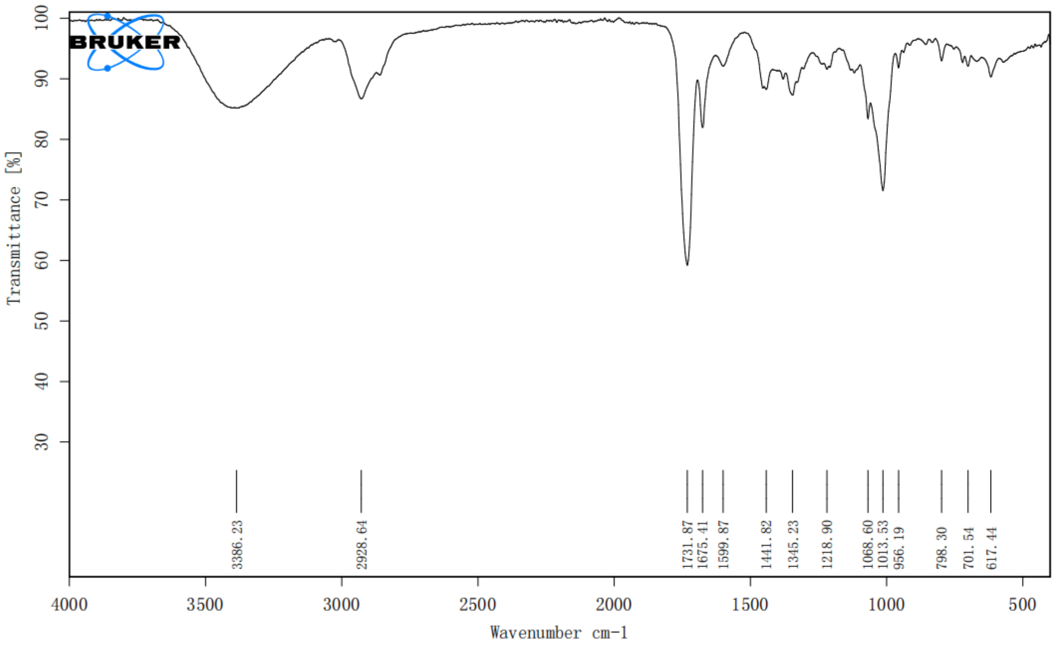

S40. ^{1}H NMR spectrum of Eutypellaolide D (**4**) in $CDCl_3$

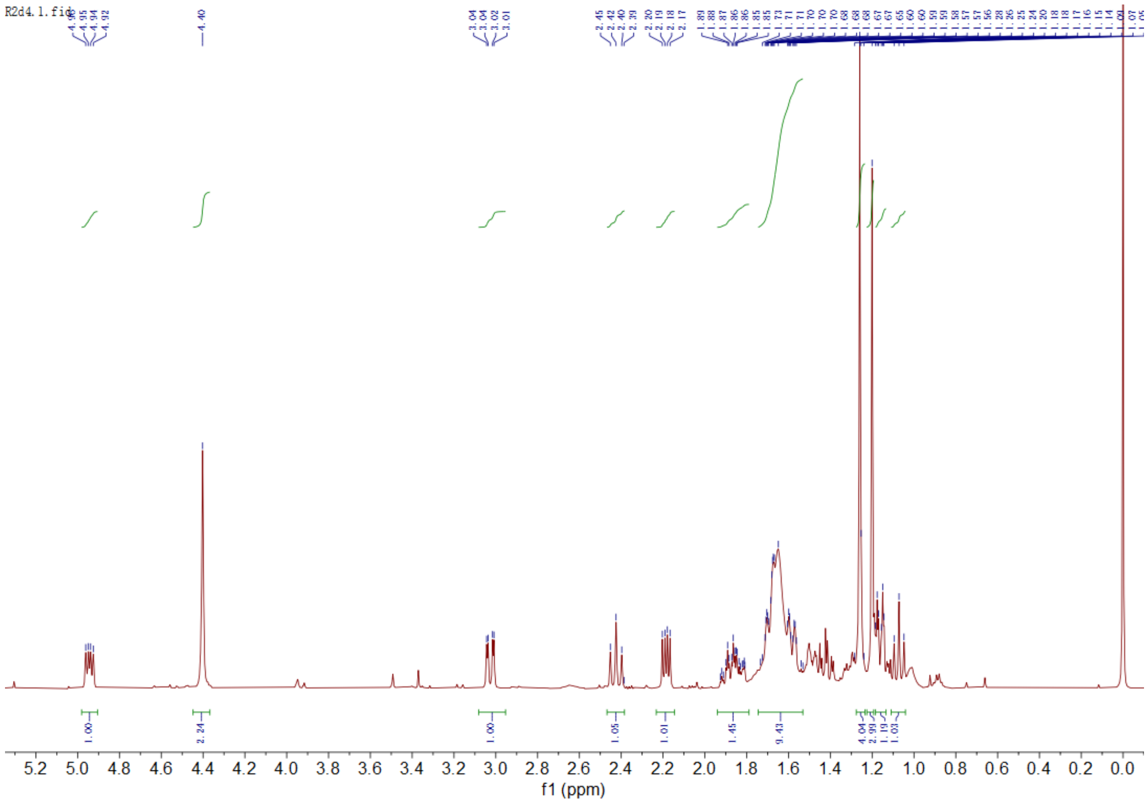

S41. ^{13}C NMR spectrum of Eutypellaolide D (**4**) in $CDCl_3$

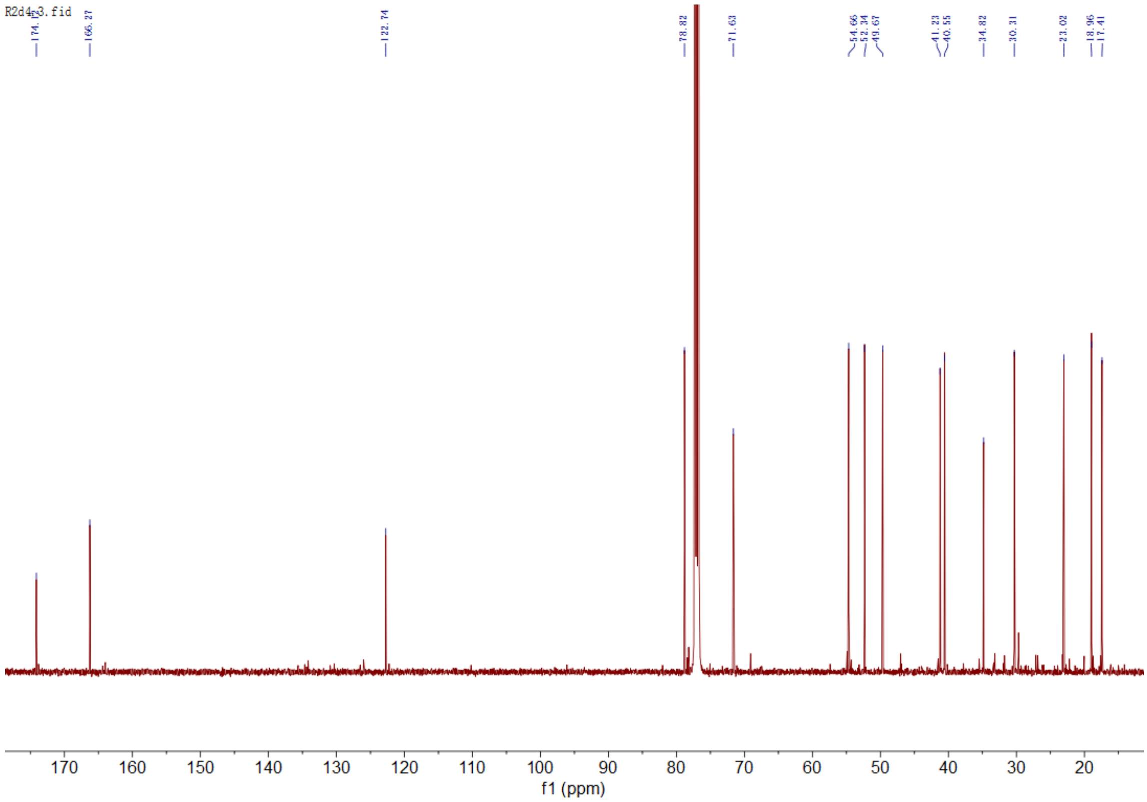

S42. DEPT135 spectrum of Eutypellaolide D (**4**) in $CDCl_3$

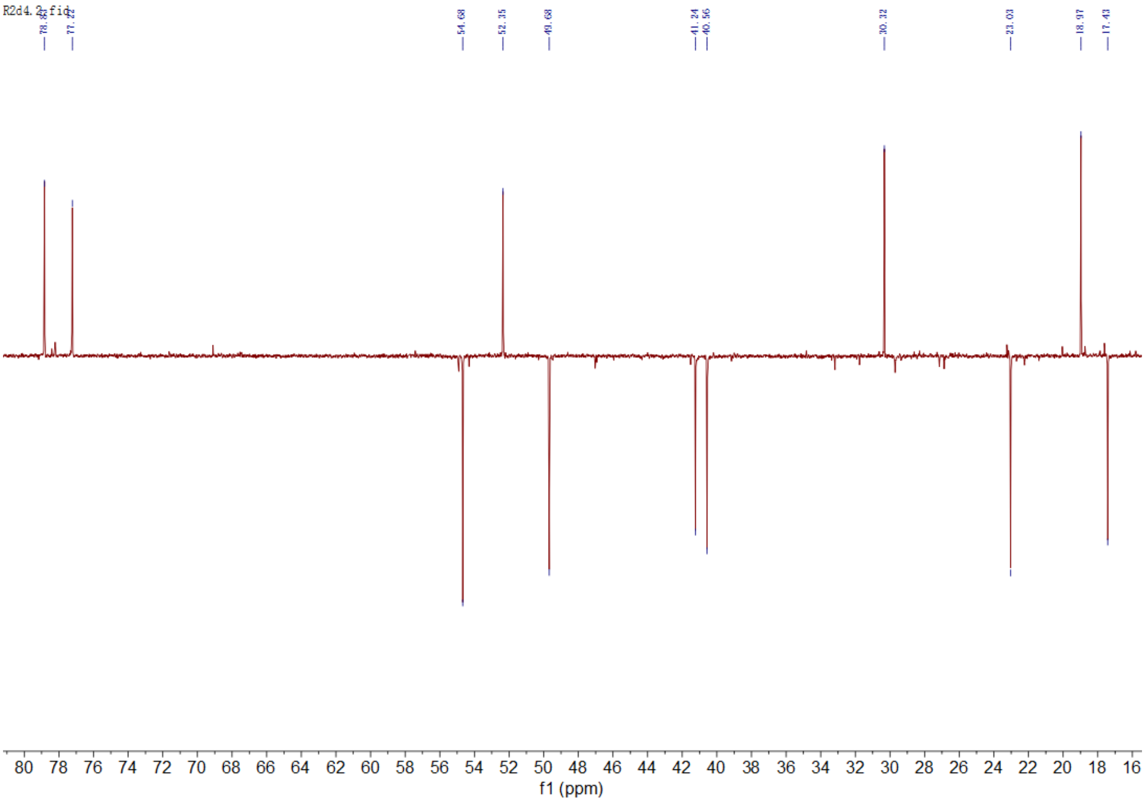

S43. HSQC spectrum of Eutypellaolide D (**4**) in $CDCl_3$

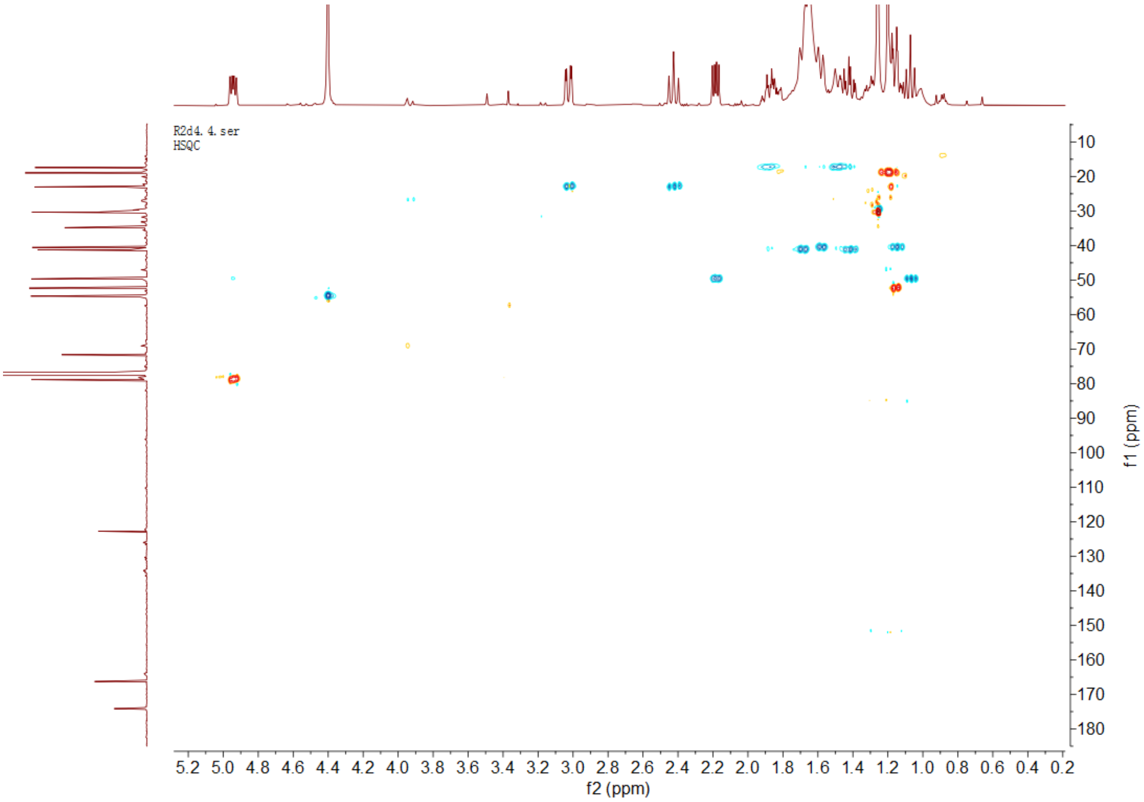

S44. COSY spectrum of Eutypellaolide D (**4**) in $CDCl_3$

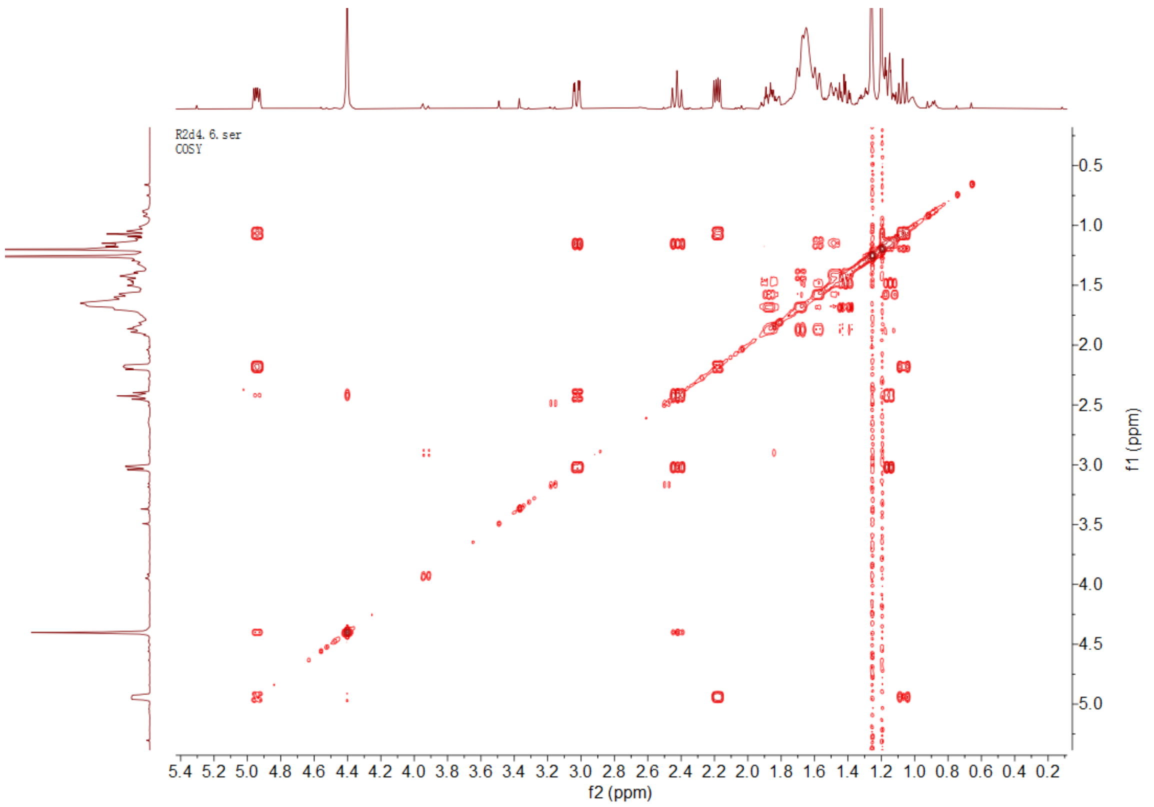

S45. HMBC spectrum of Eutypellaolide D (**4**) in $CDCl_3$

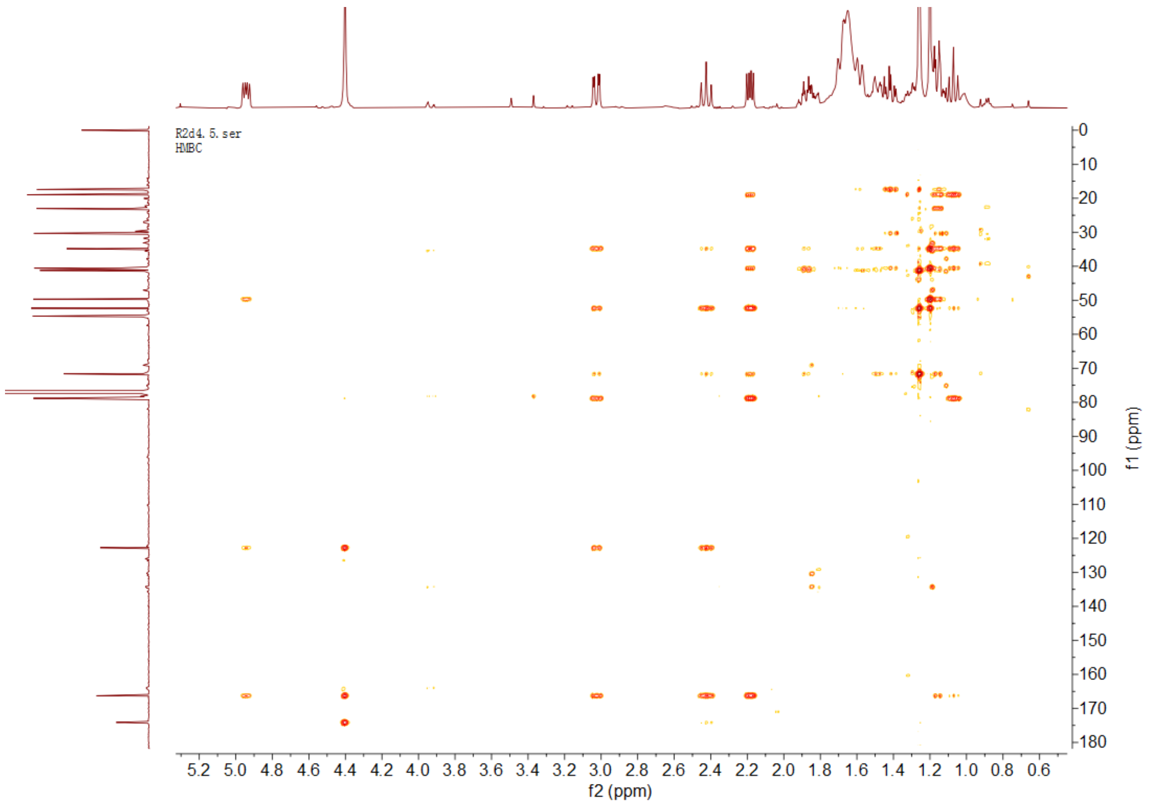

S46. NOESY spectrum of Eutypellaolide D (**4**) in $CDCl_3$

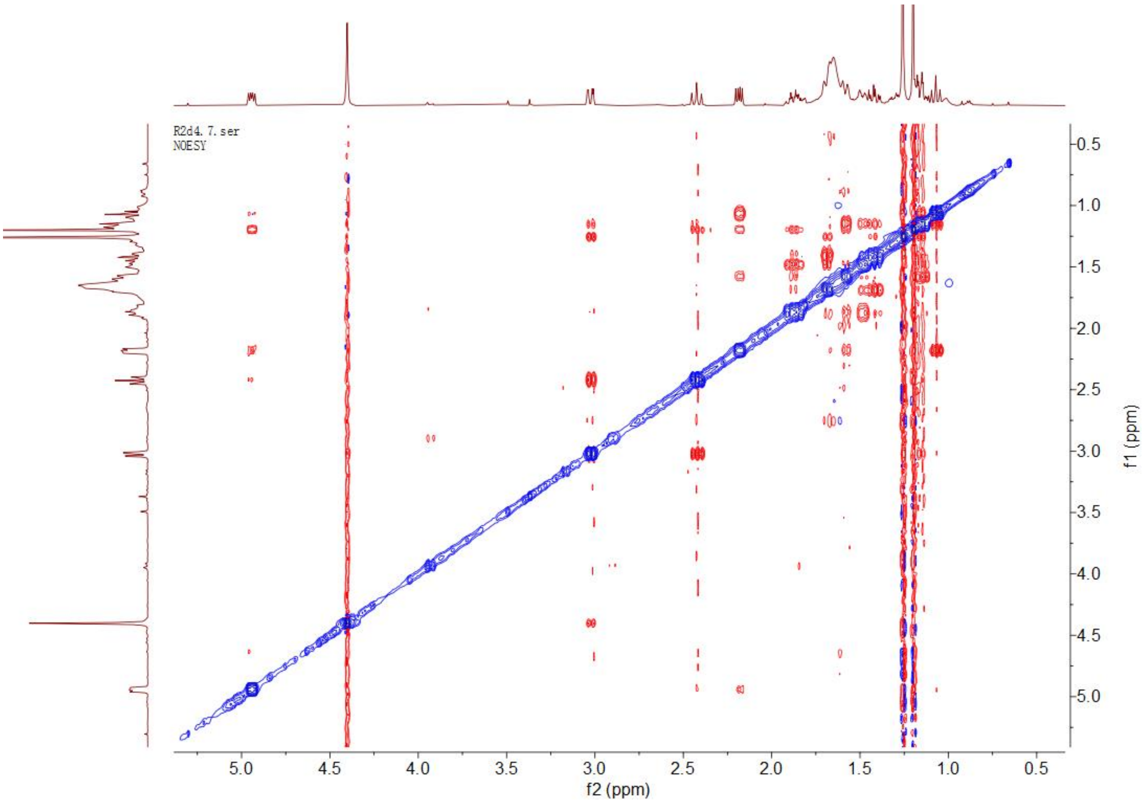

S47. HRESIMS of Eutypellaolide D (**4**)

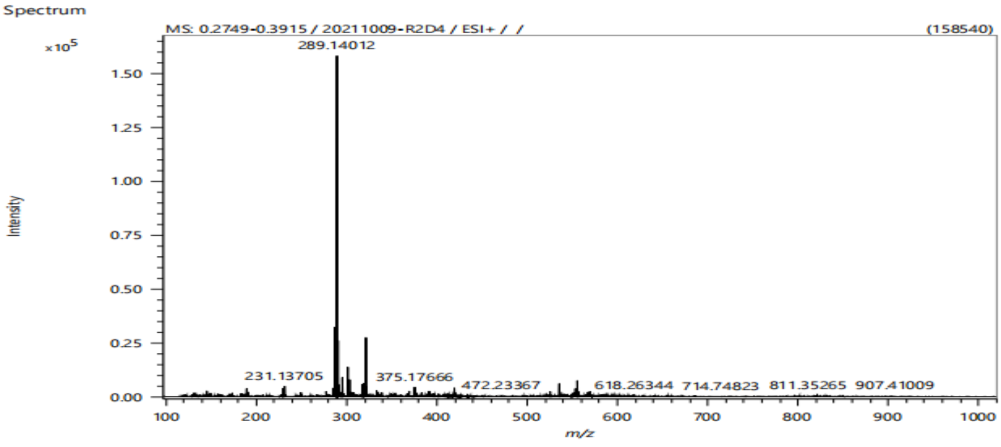

Elemental Composition

Parameters	
Tolerance:	±5.00 ppm
Electron:	Odd/Even
Charge:	+1
DBE:	-1.5 - 200.0

Elements Set 1:

Symbol	C	H	N	O	Na	S	Cl	Br
Min	0	0	0	0	1	0	0	0
Max	200	200	0	8	1	0	0	0

Symbol	P	Si	F
Min	0	0	0
Max	0	0	0

Results

Mass	Intensity	Intensity [%]	Formula	Calculated Mass	Mass Difference [mDa]	Mass Difference [ppm]	DBE
289.14012	158540.12	100.00	C15 H22 O4 Na	289.14103	-0.91	-3.16	4.5

S48. UV spectrum of Eutypellaolide D (**4**) in MeOH

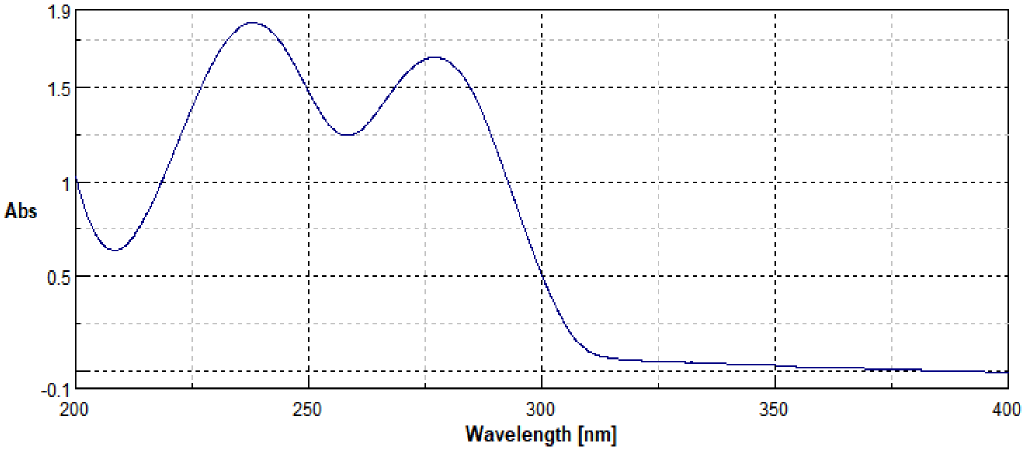

S49. IR spectrum of Eutypellaolide D (**4**)

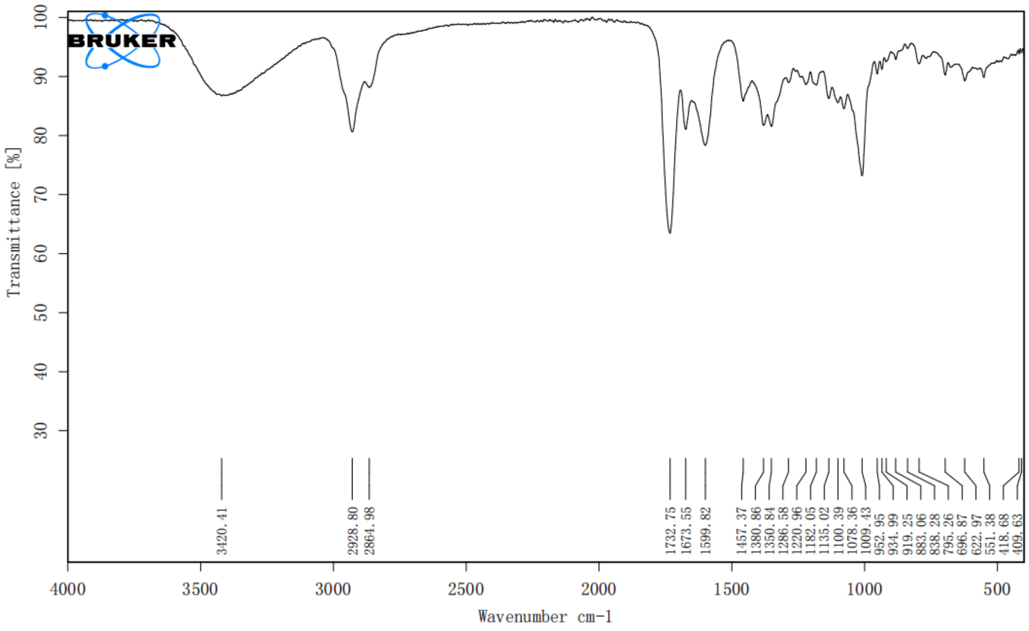

S50. ^{1}H NMR spectrum of Eutypellaolide E (**5**) in MeOH

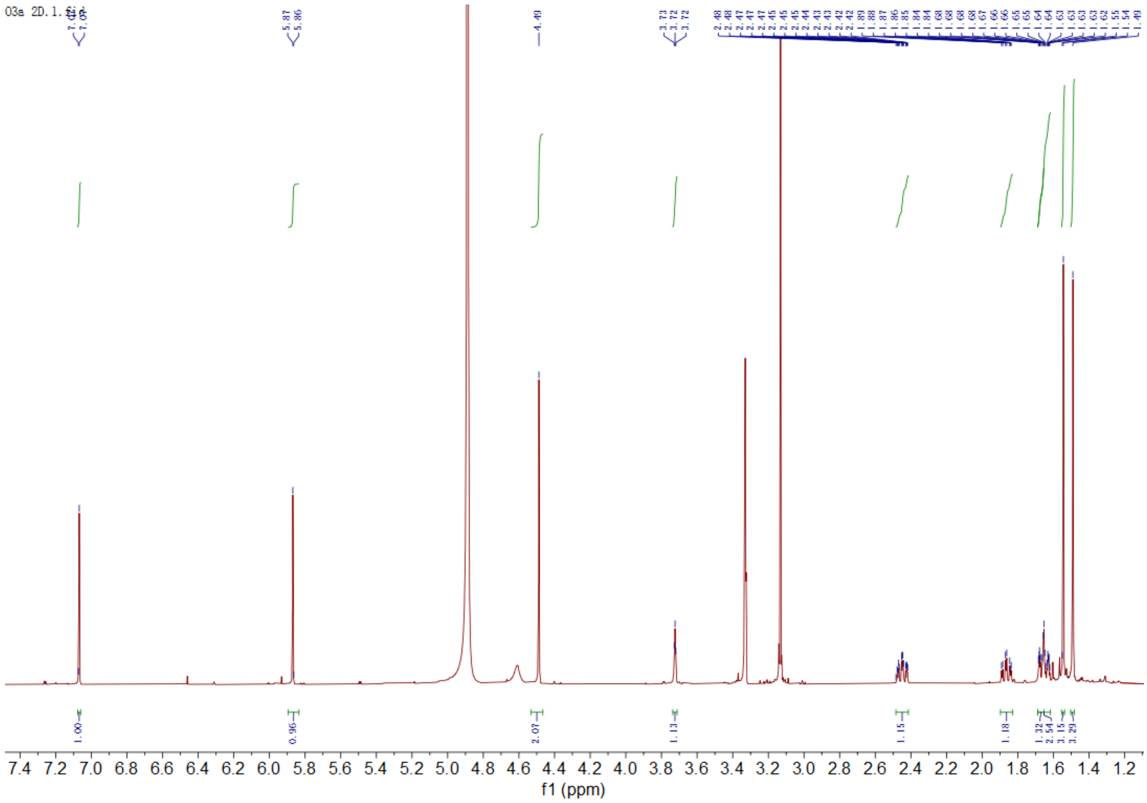

S51. ^{13}C NMR spectrum of Eutypellaolide E (**5**) in MeOH

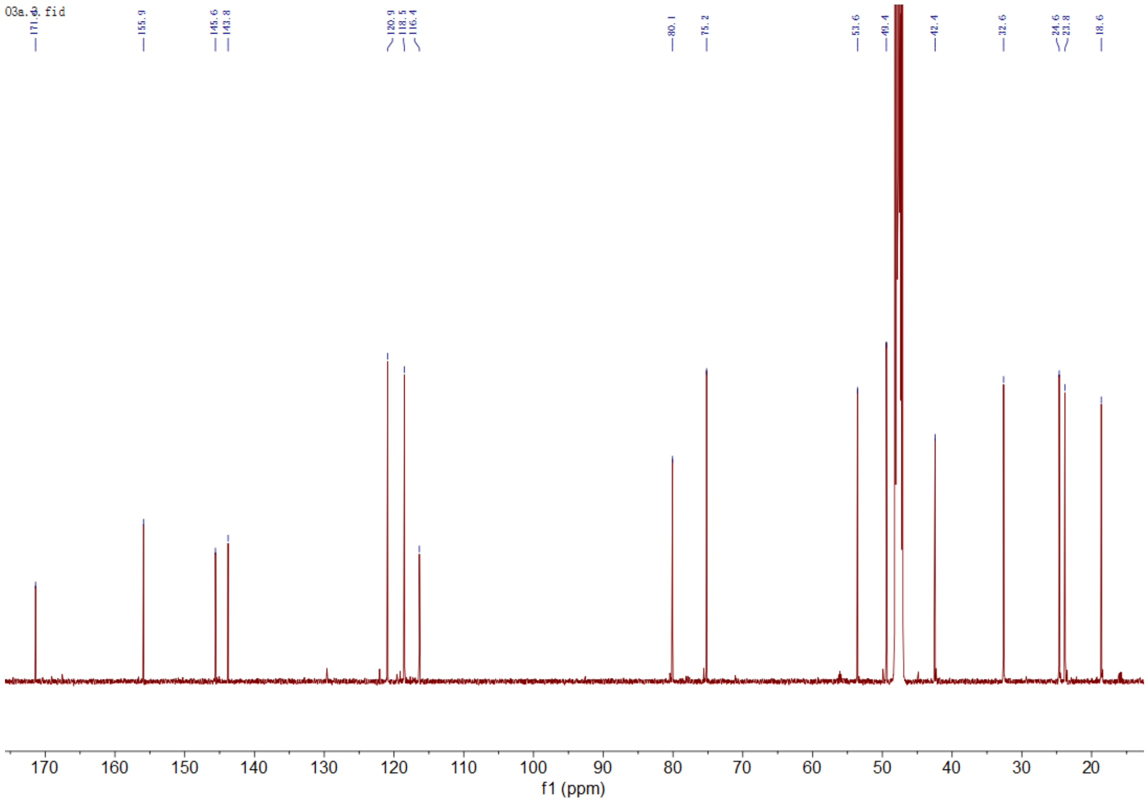

S52. DEPT135 spectrum of Eutypellaolide E (**5**) in MeOH

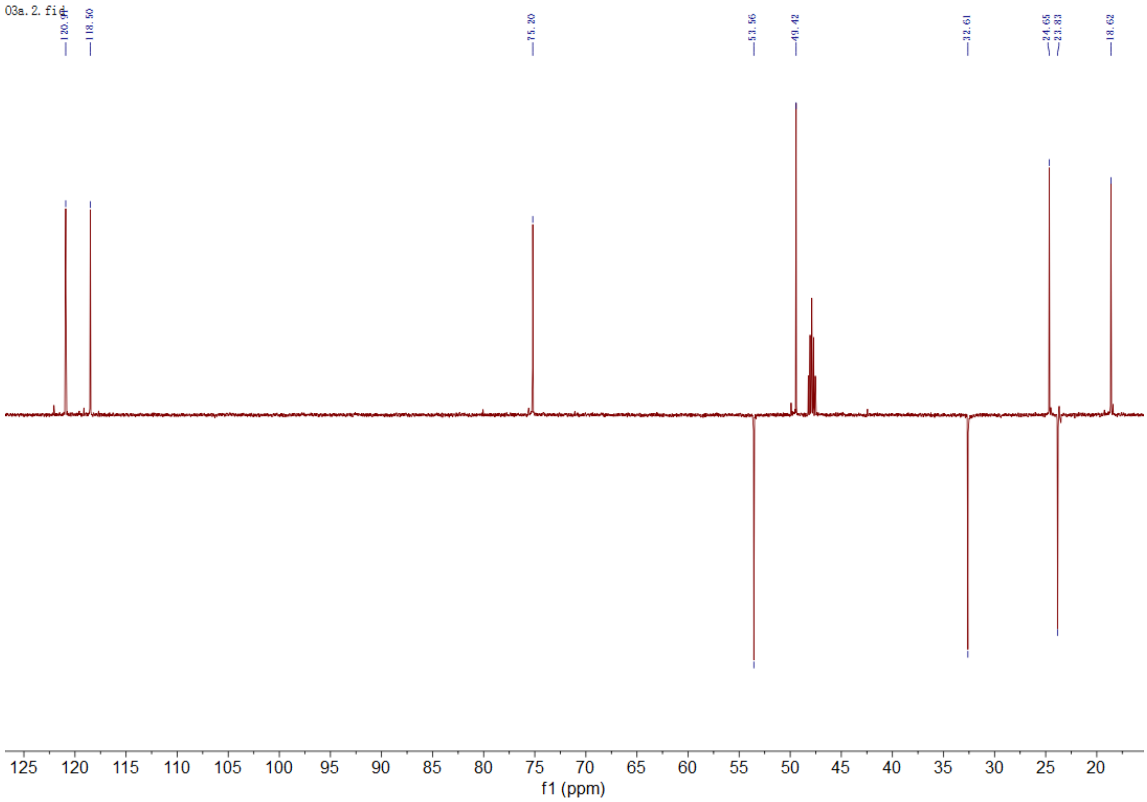

S53. HSQC spectrum of Eutypellaolide E (**5**) in MeOH

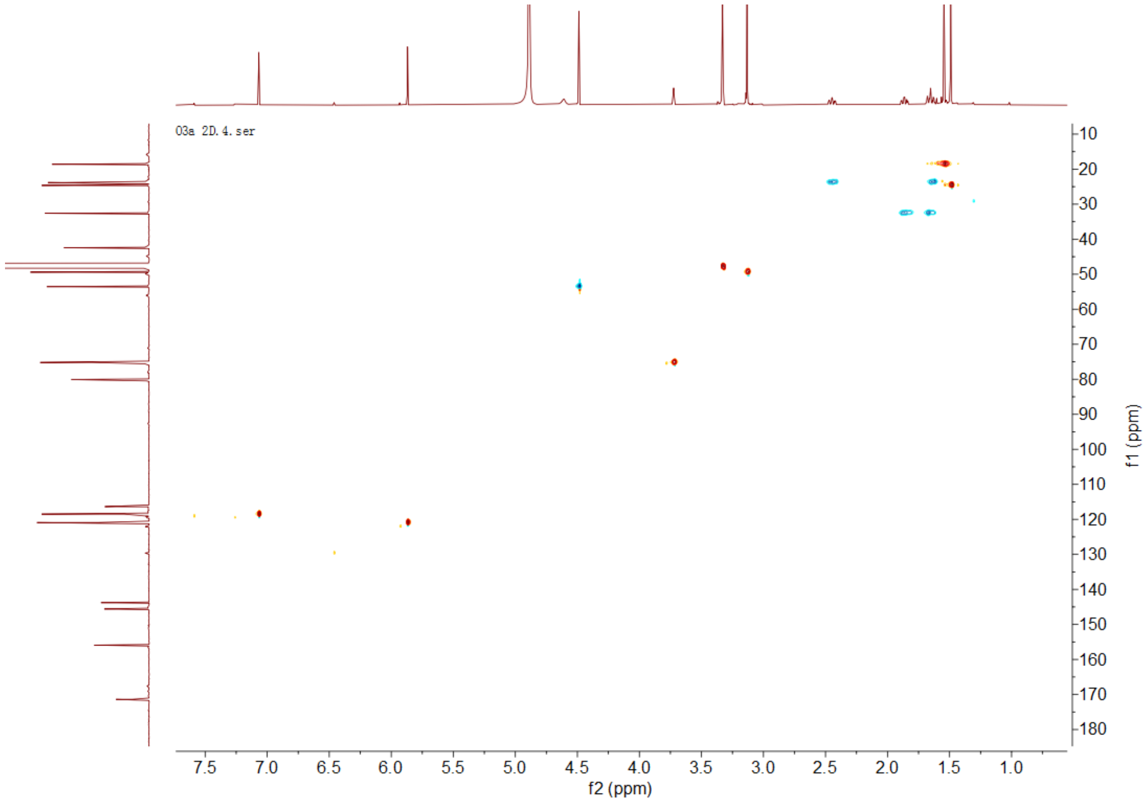

S54. COSY spectrum of Eutypellaolide E (**5**) in MeOH

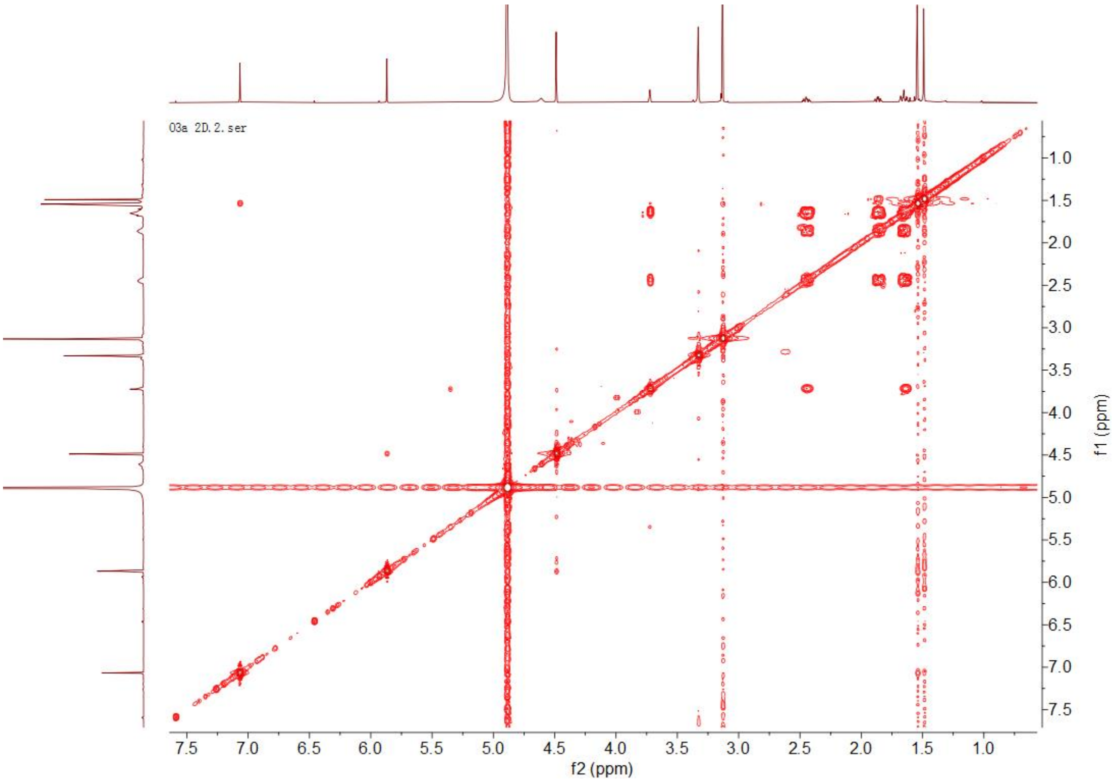

S55. HMBC spectrum of Eutypellaolide E (**5**) in MeOH

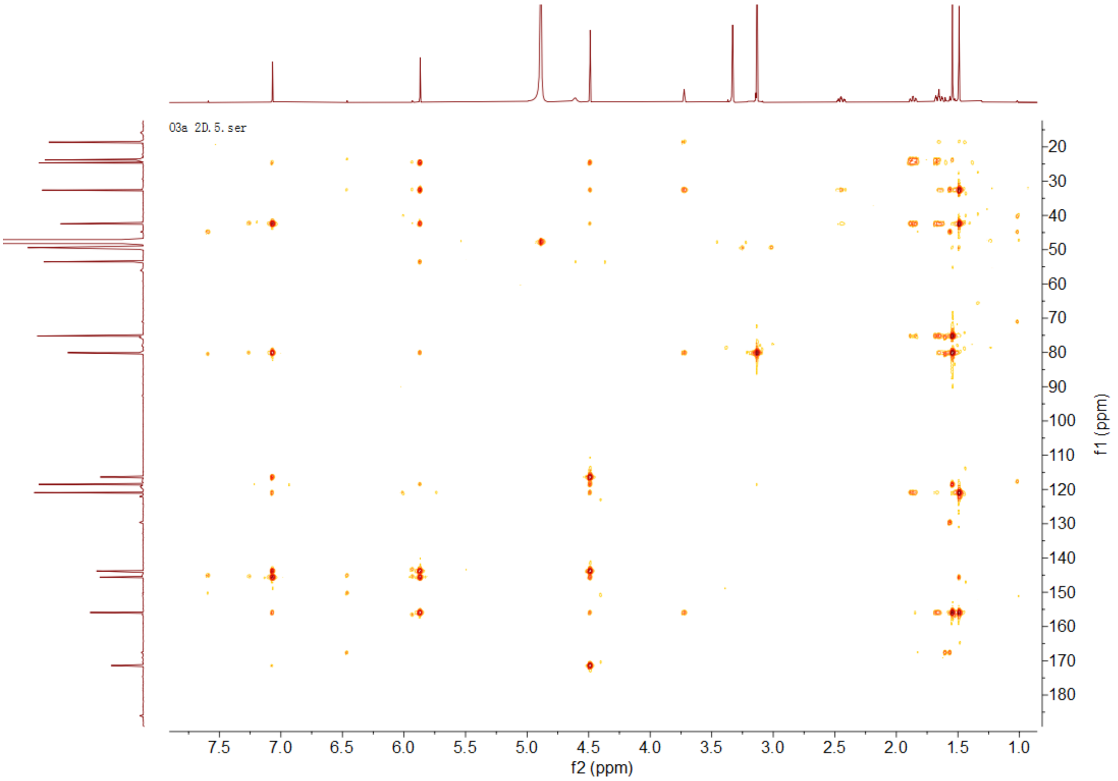

S56. NOESY spectrum of Eutypellaolide E (**5**) in MeOH

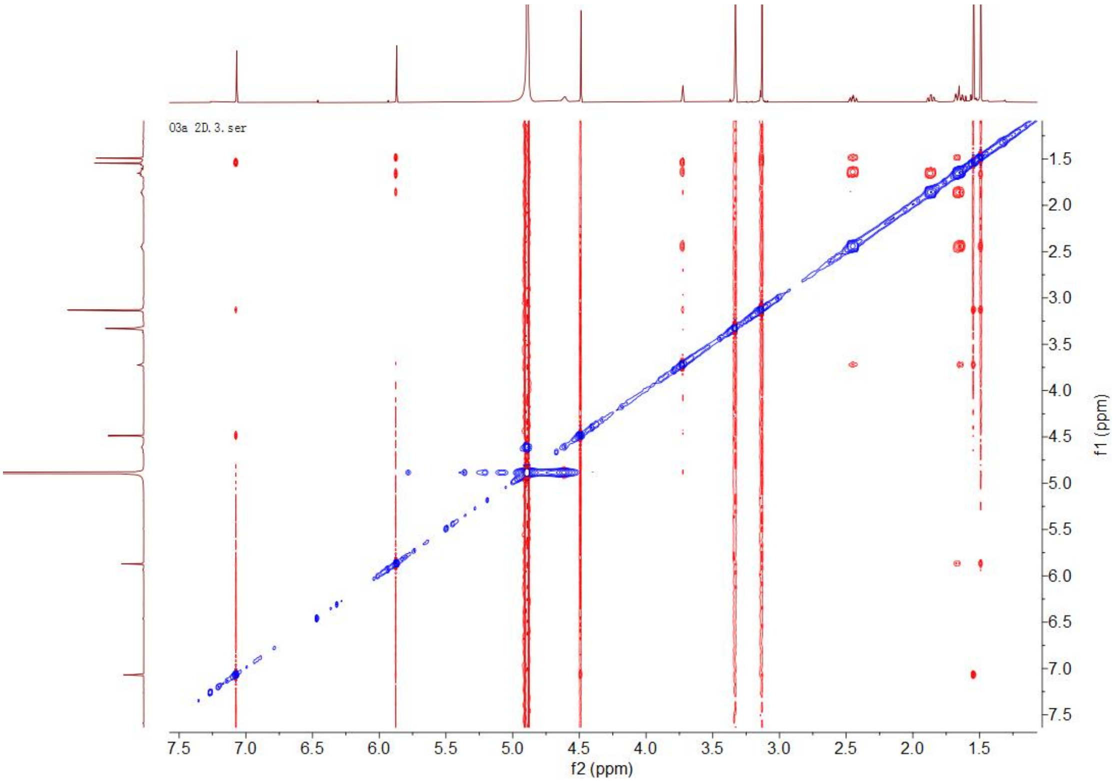

S57. HRESIMS of Eutypellaolide E (**5**)

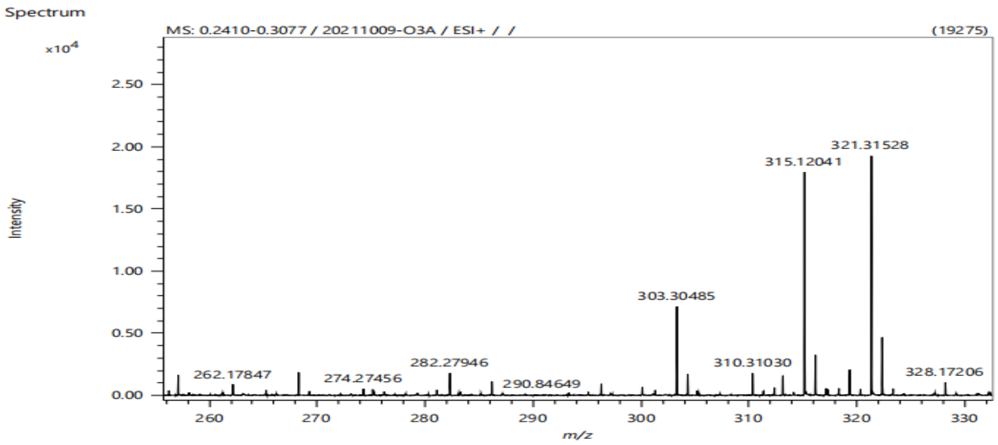

Elemental Composition

Parameters	
Tolerance:	±5.00 ppm
Electron:	Odd/Even
Charge:	+1
DBE:	-1.5 - 200.0

Elements Set 1:

Symbol	C	H	N	O	Na	S	Cl	Br
Min	0	0	0	0	1	0	0	0
Max	200	200	0	8	1	0	0	0

Symbol	P	Si	F
Min	0	0	0
Max	0	0	0

Results

Mass	Intensity	Intensity [%]	Formula	Calculated Mass	Mass Difference [mDa]	Mass Difference [ppm]	DBE
315.12041	17972.31	10.48	C16 H20 O5 Na	315.12029	0.12	0.37	6.5

S58. UV spectrum of Eutypellaolide E (**5**) in MeOH

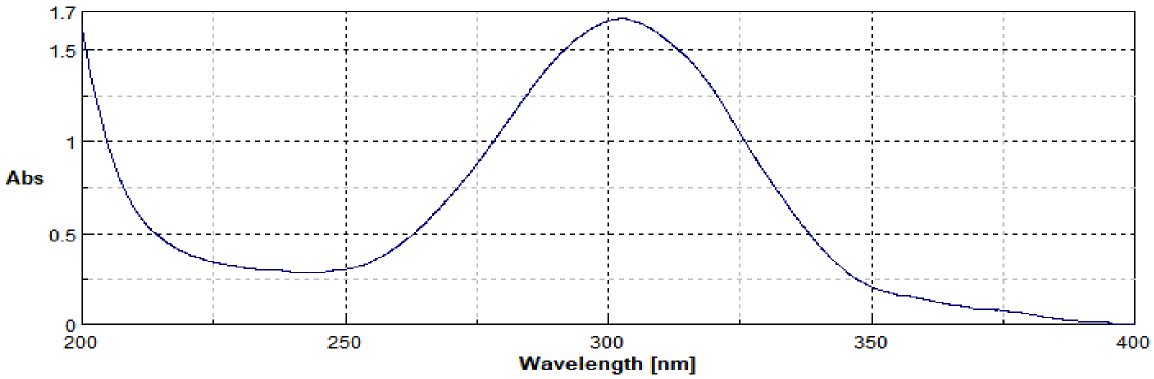

S59. IR spectrum of Eutypellaolide E (**5**)

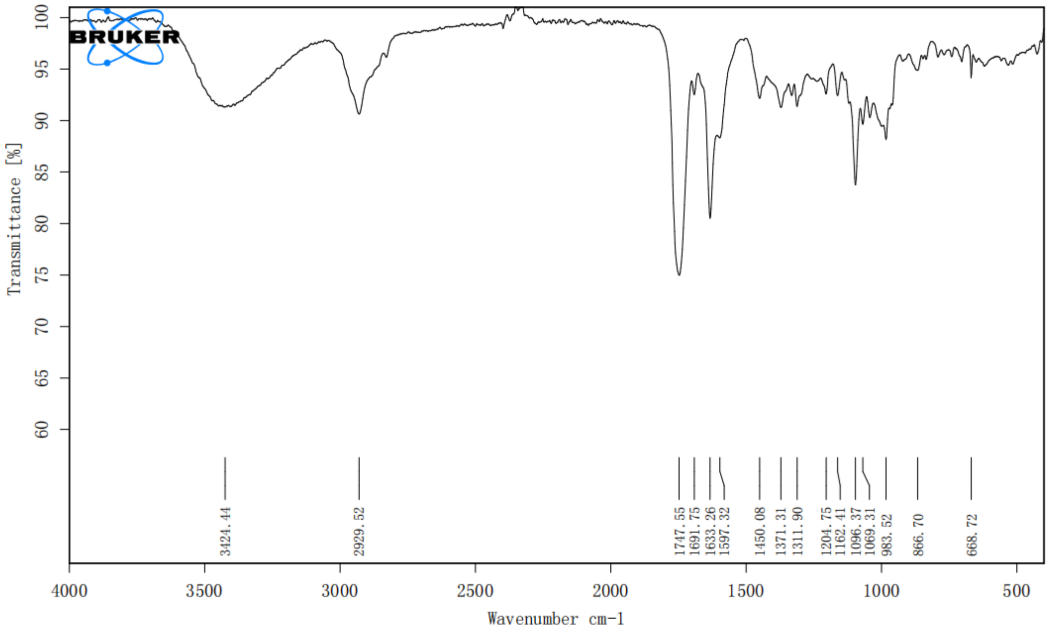

S60. ^{1}H NMR spectrum of Eutypellaolide F (**6**) in MeOH

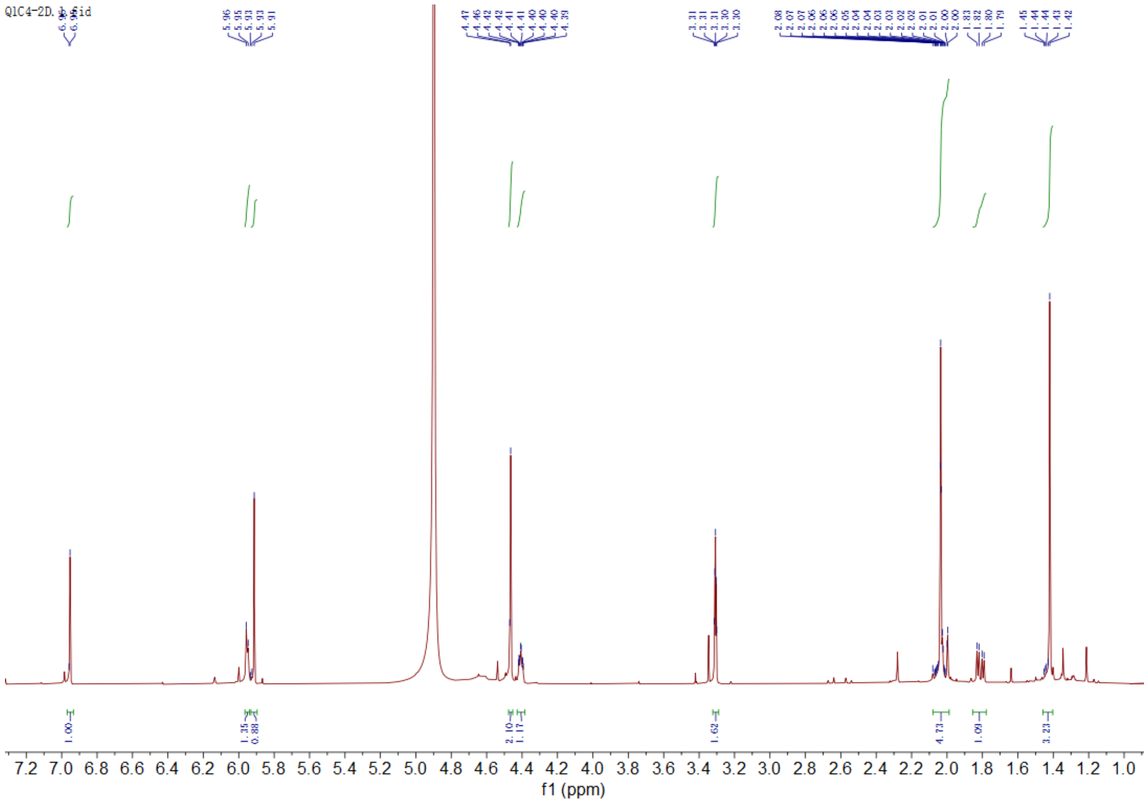

S61. ^{13}C NMR spectrum of Eutypellaolide F (**6**) in MeOH

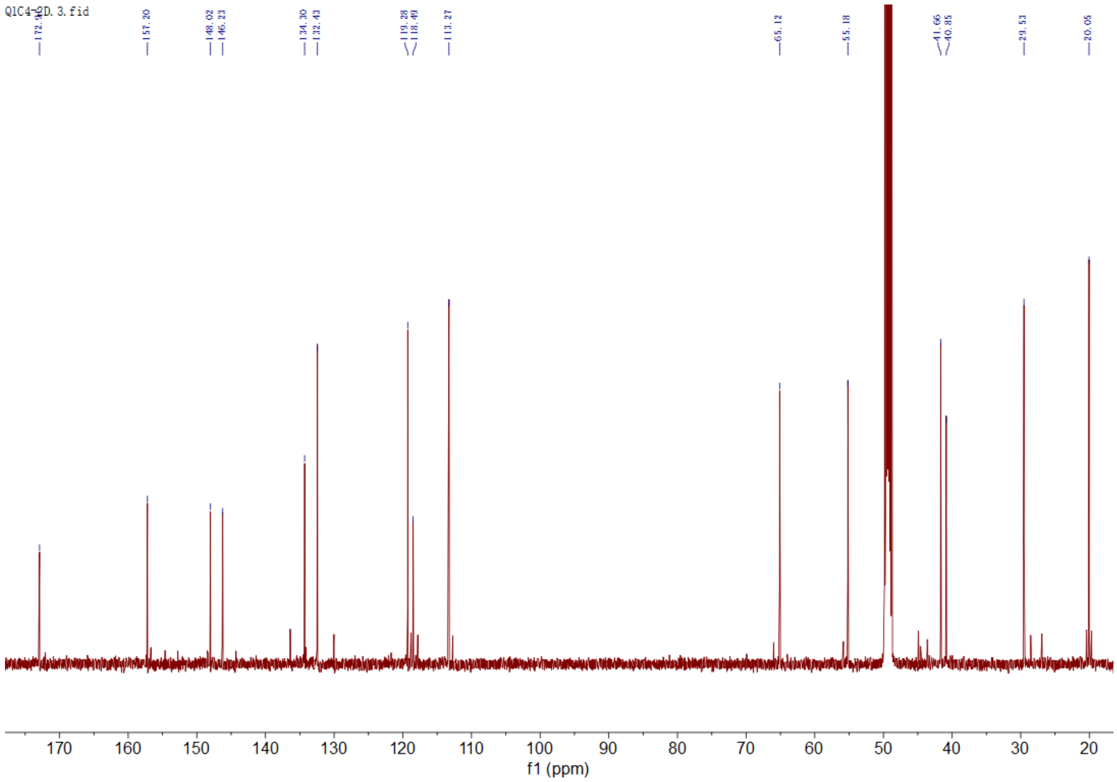

S62. DEPT135 spectrum of Eutypellaolide F (**6**) in MeOH

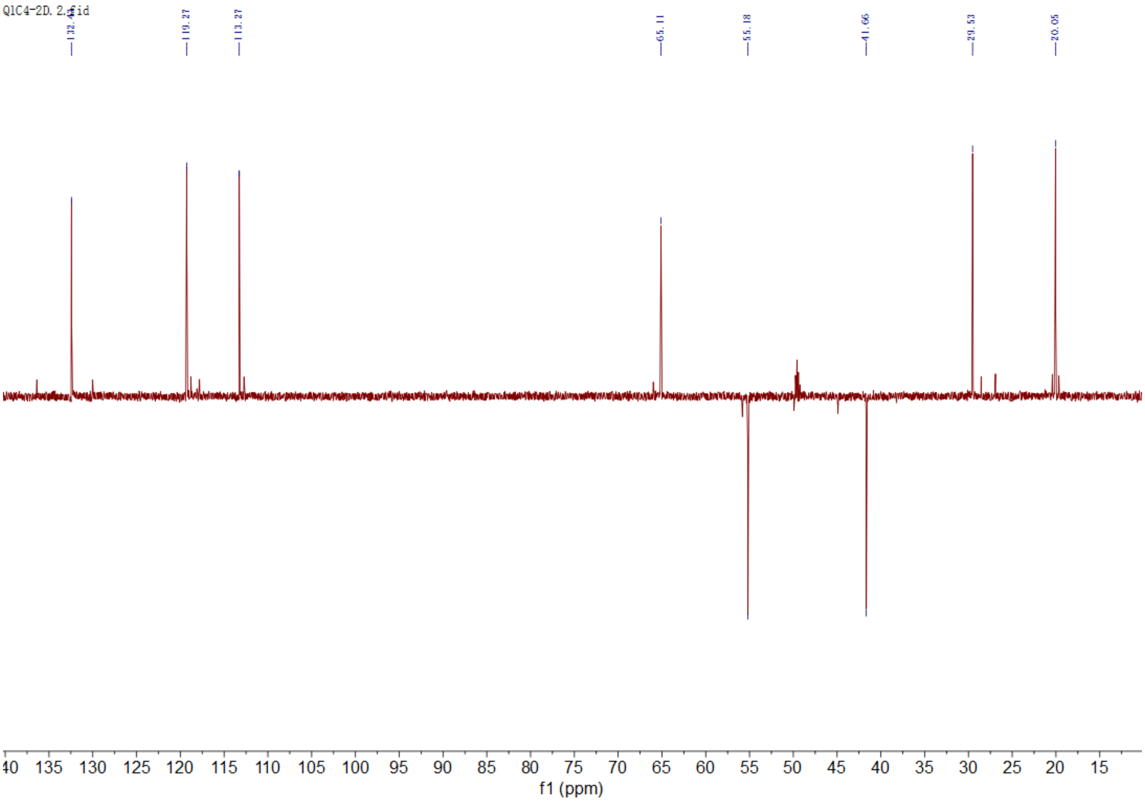

S63. HSQC spectrum of Eutypellaolide F (**6**) in MeOH

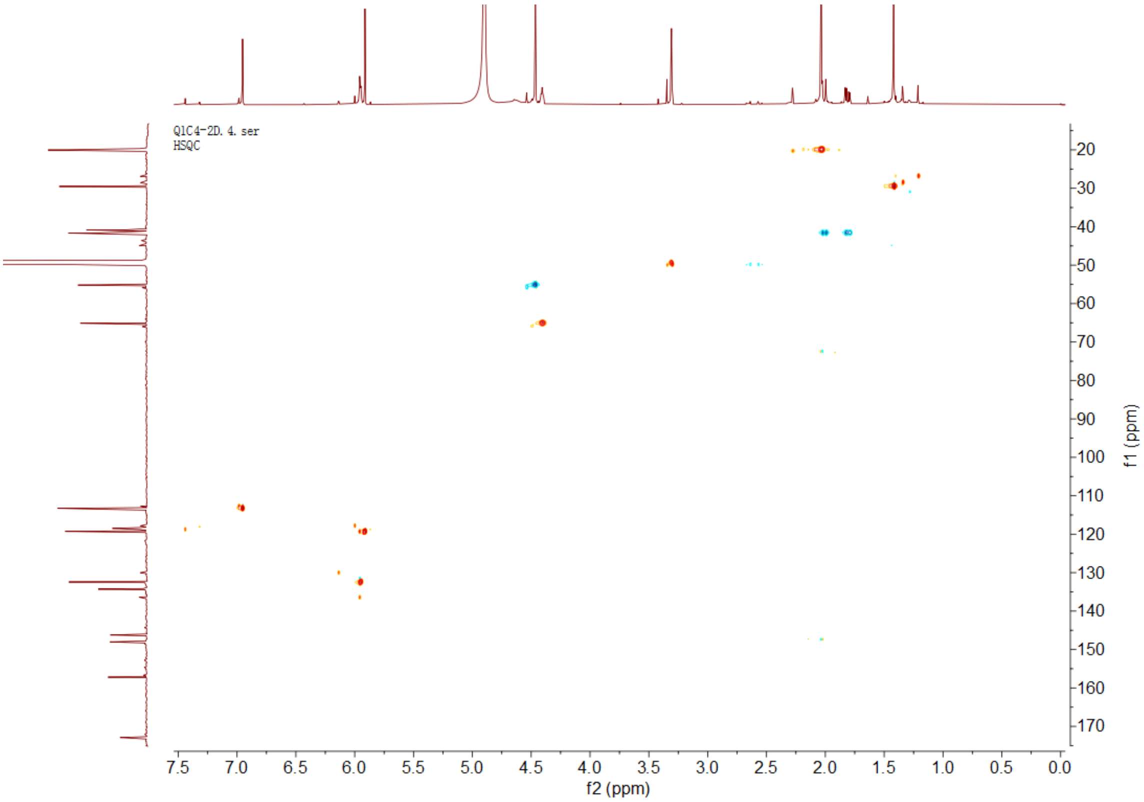

S64. COSY spectrum of Eutypellaolide F (**6**) in MeOH

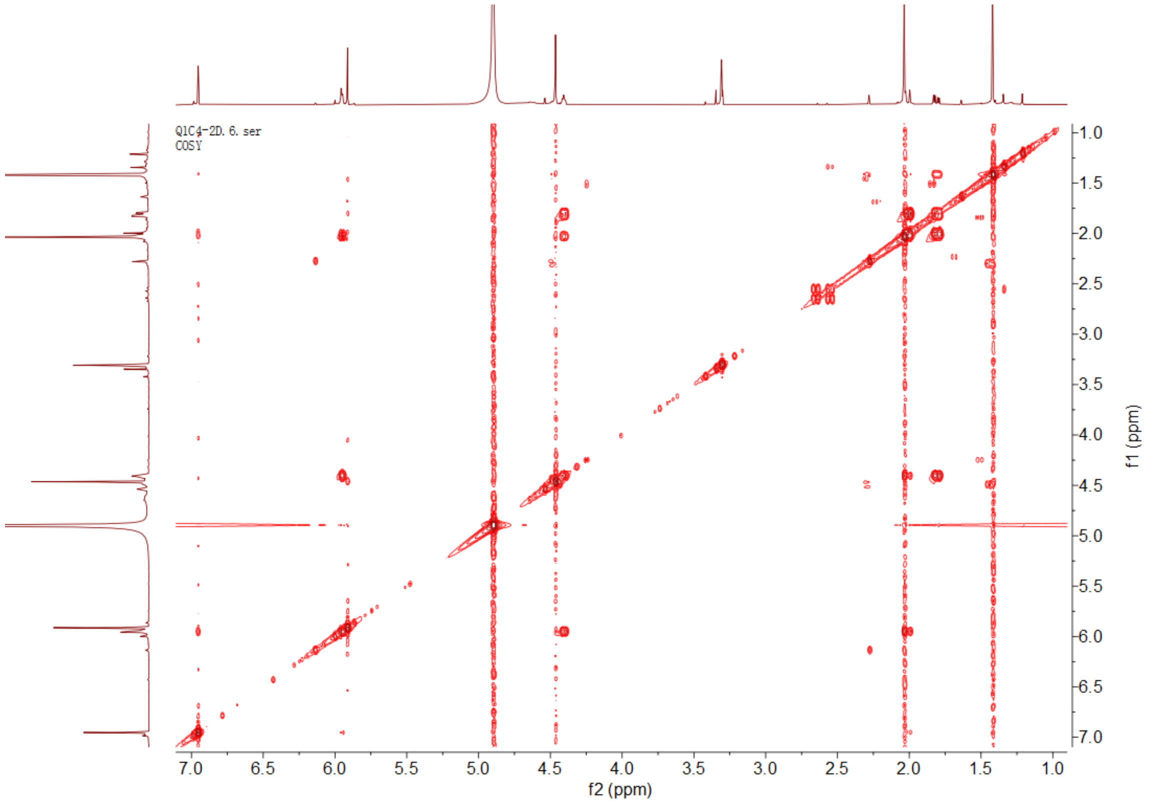

S65. HMBC spectrum of Eutypellaolide F (**6**) in MeOH

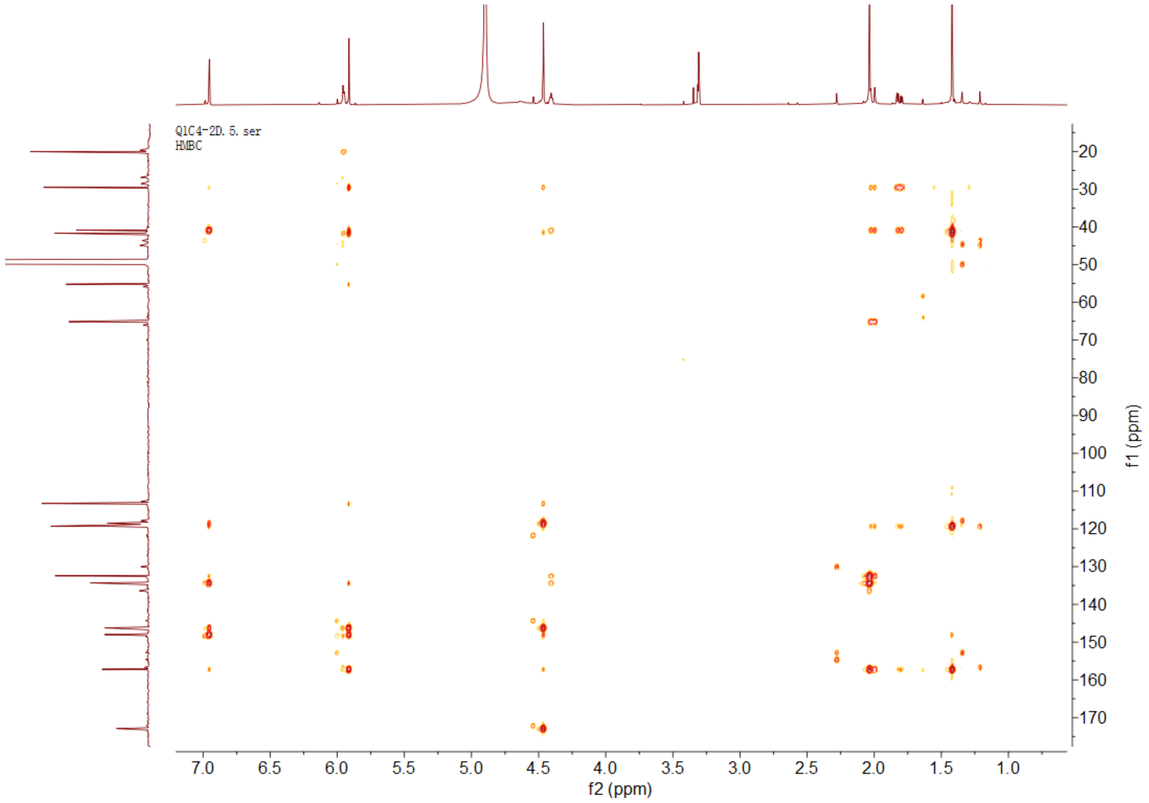

S66. NOESY spectrum of Eutypellaolide F (**6**) in MeOH

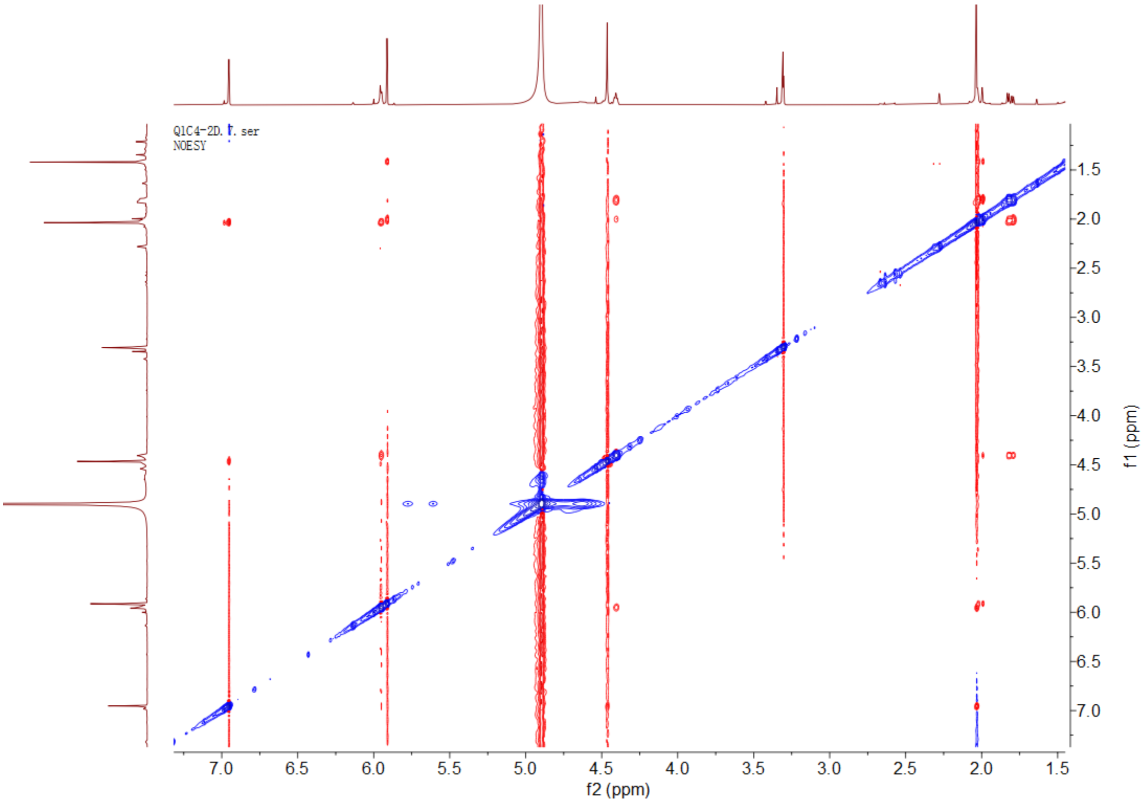

S67. HRESIMS of Eutypellaolide F (**6**)

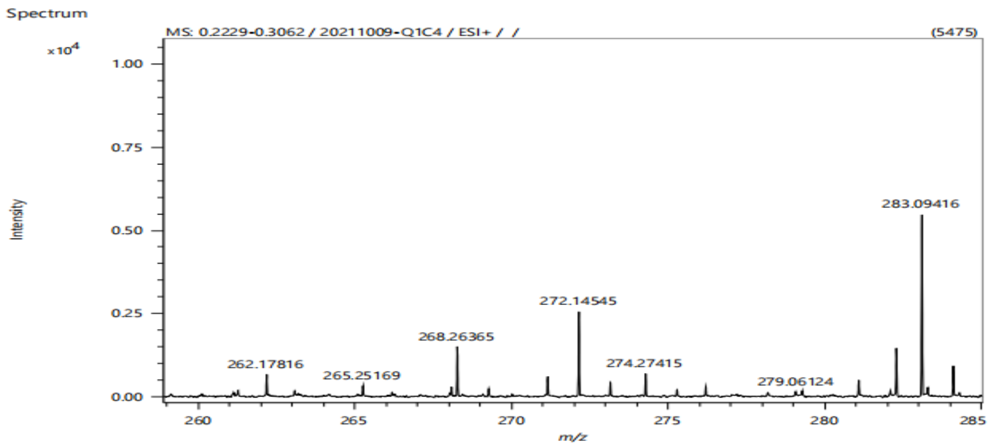

Elemental Composition

Parameters

Tolerance:	±20.00 ppm
Electron:	Odd/Even
Charge:	+1
DBE:	-1.5 - 200.0

Elements Set 1:

Symbol	C	H	N	O	Na	S	Cl	Br
Min	0	0	0	0	1	0	0	0
Max	200	200	0	8	1	0	0	0

Symbol	P	Si	F
Min	0	0	0
Max	0	0	0

Results

Mass	Intensity	Intensity [%]	Formula	Calculated Mass	Mass Difference [mDa]	Mass Difference [ppm]	DBE
283.09416	5475.38	3.92	C15 H16 O4 Na	283.09408	0.08	0.29	7.5

S68. UV spectrum of Eutypellaolide F (**6**) in MeOH

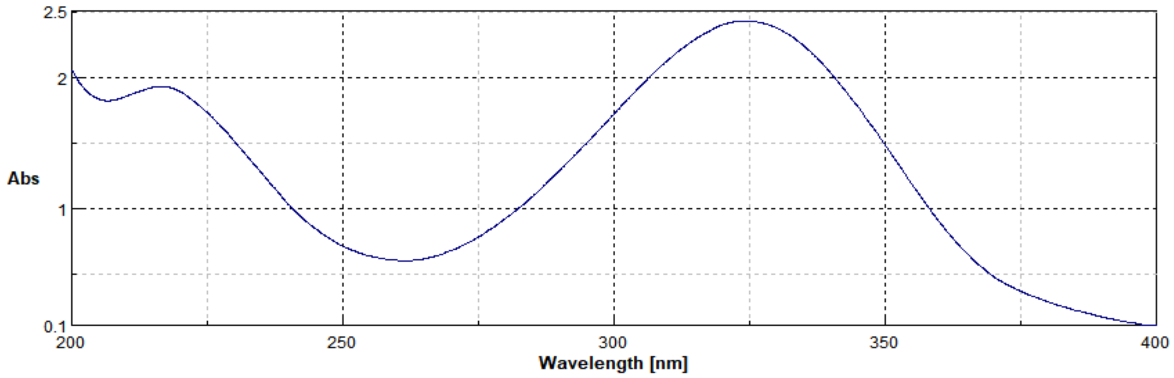

S69. IR spectrum of Eutypellaolide F (**6**)

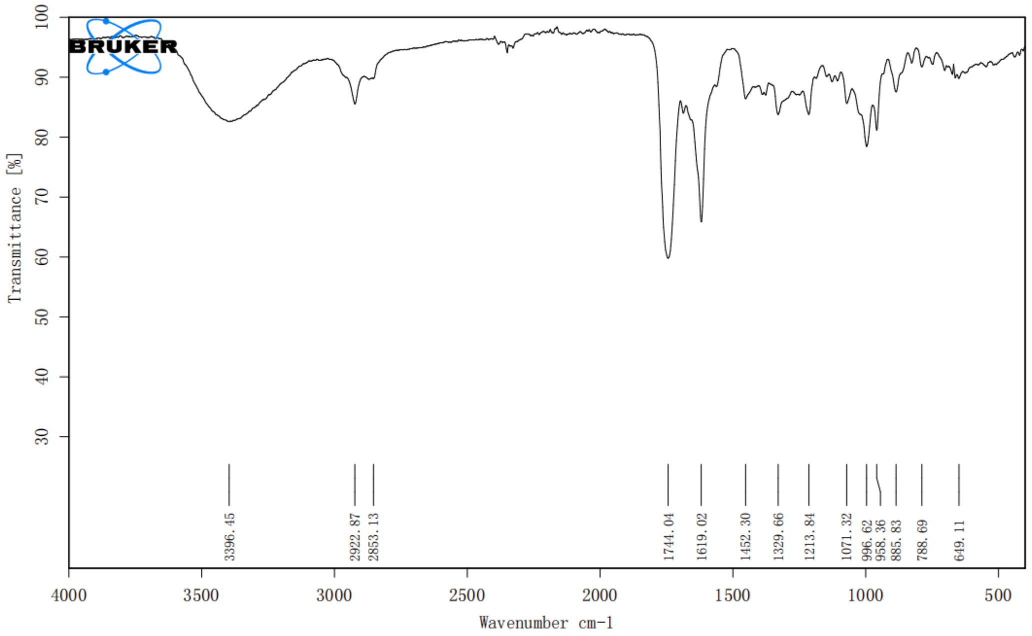

S70. ^{1}H NMR spectrum of Eutypellaolide G (**7**) in $CDCl_3$

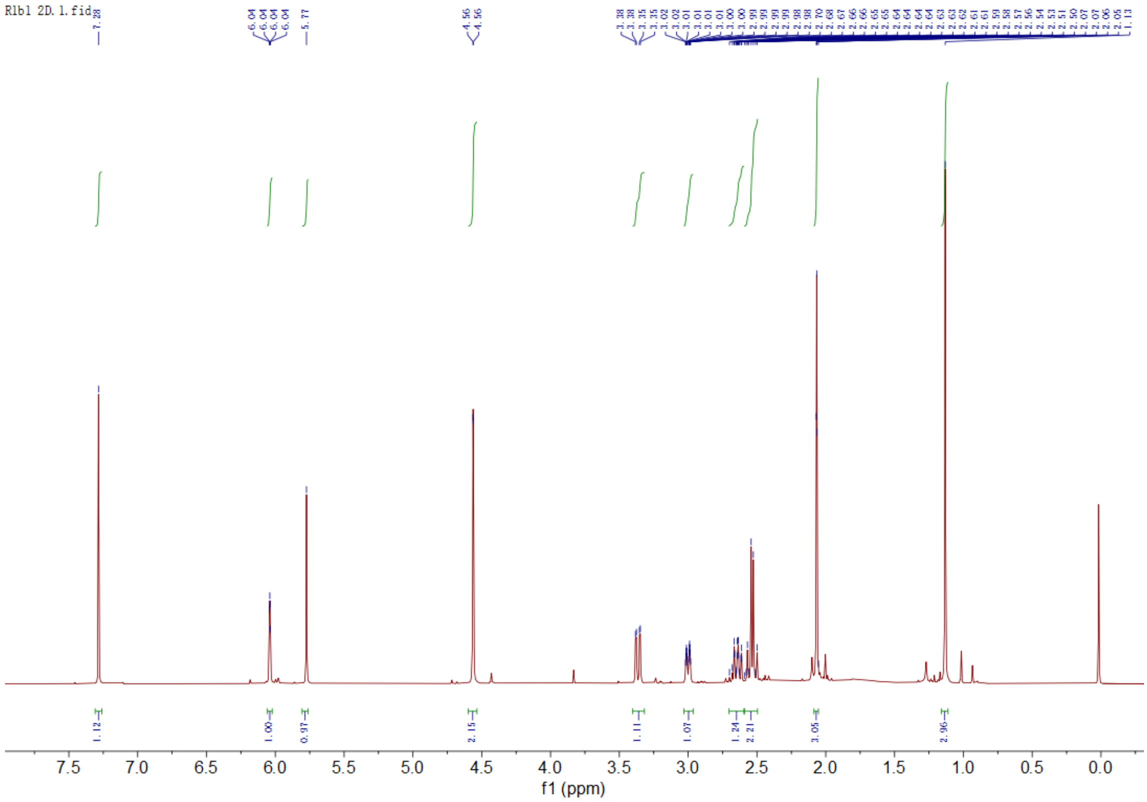

S71. ^{13}C NMR spectrum of Eutypellaolide G (**7**) in $CDCl_3$

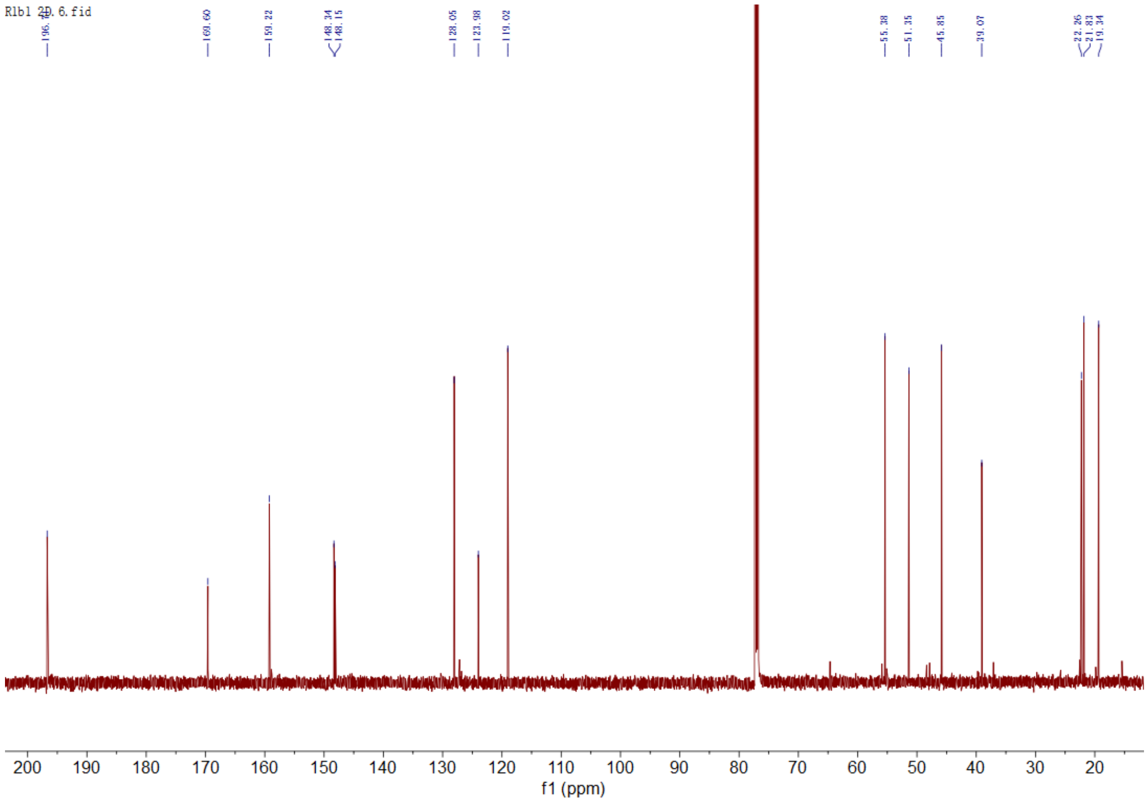

S72. DEPT135 spectrum of Eutypellaolide G (**7**) in $CDCl_3$

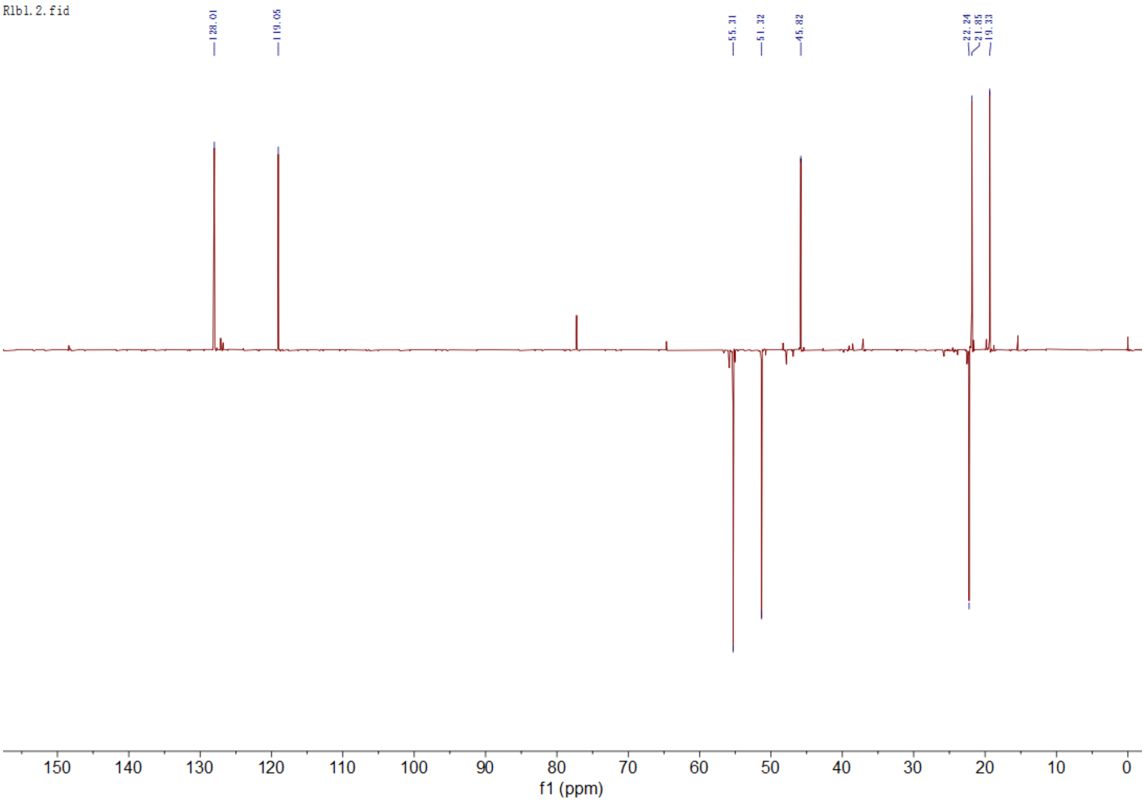

S73. HSQC spectrum of Eutypellaolide G (**7**) in $CDCl_3$

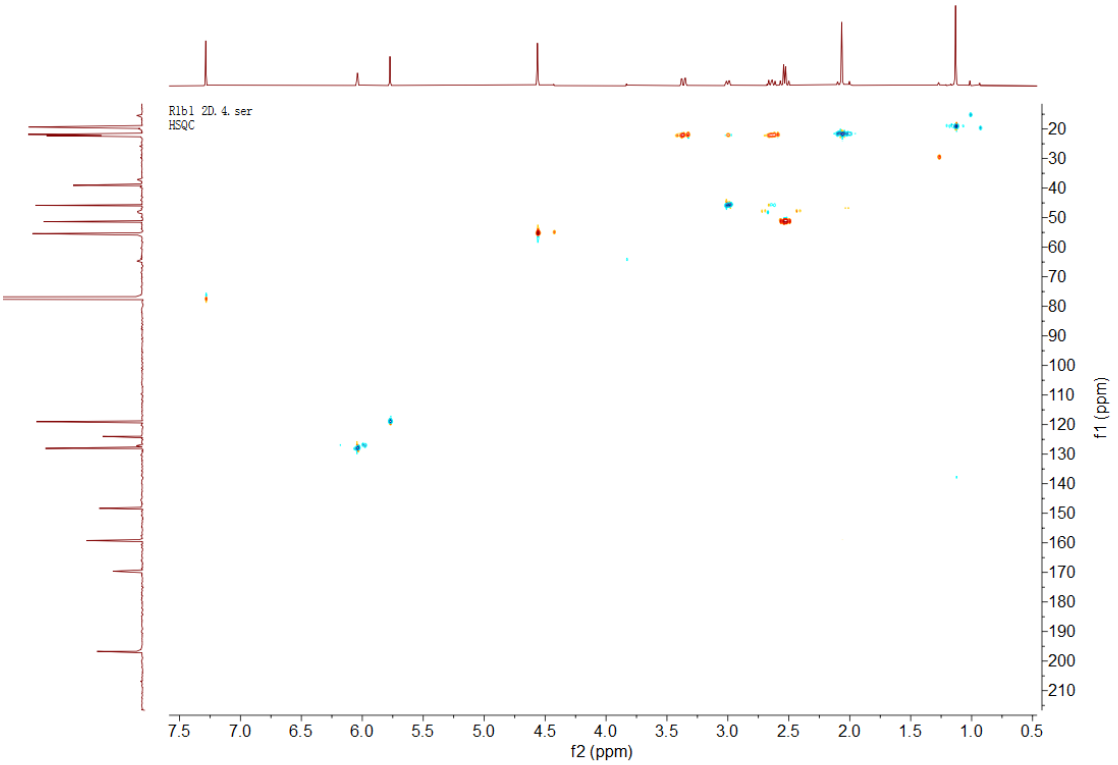

S74. COSY spectrum of Eutypellaolide G (**7**) in $CDCl_3$

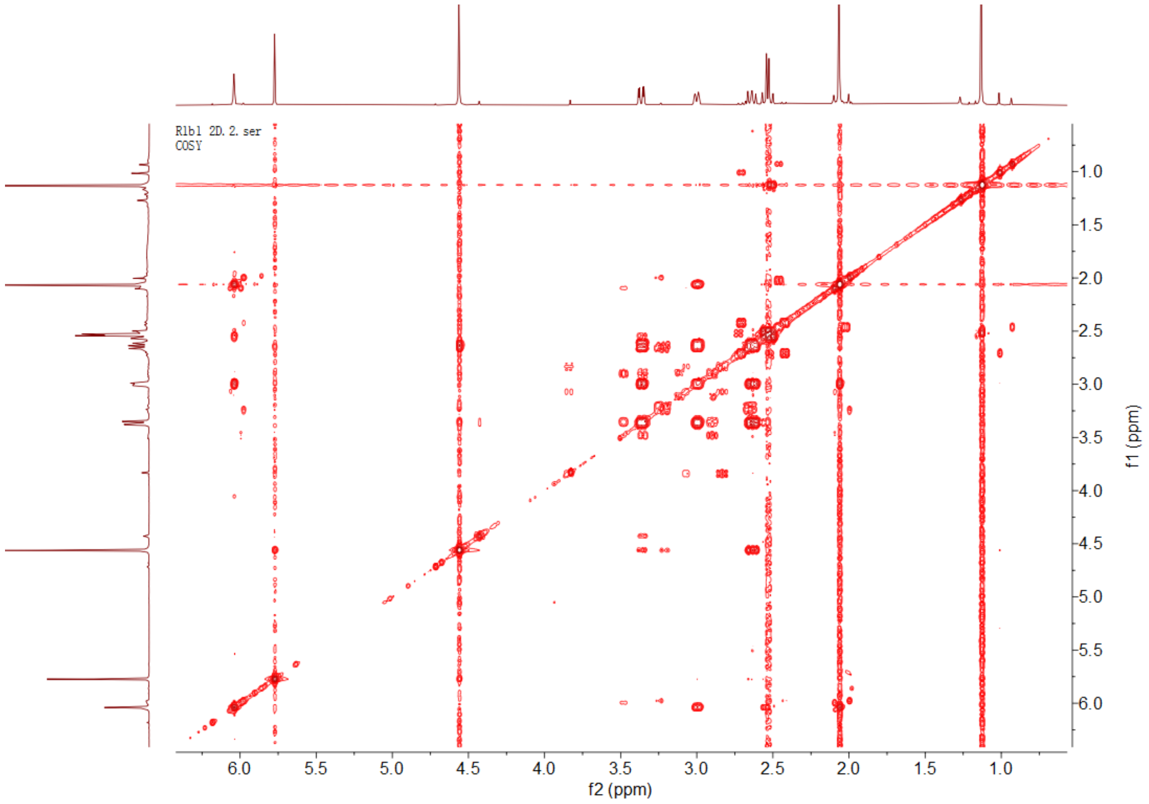

S75. HMBC spectrum of Eutypellaolide G (**7**) in $CDCl_3$

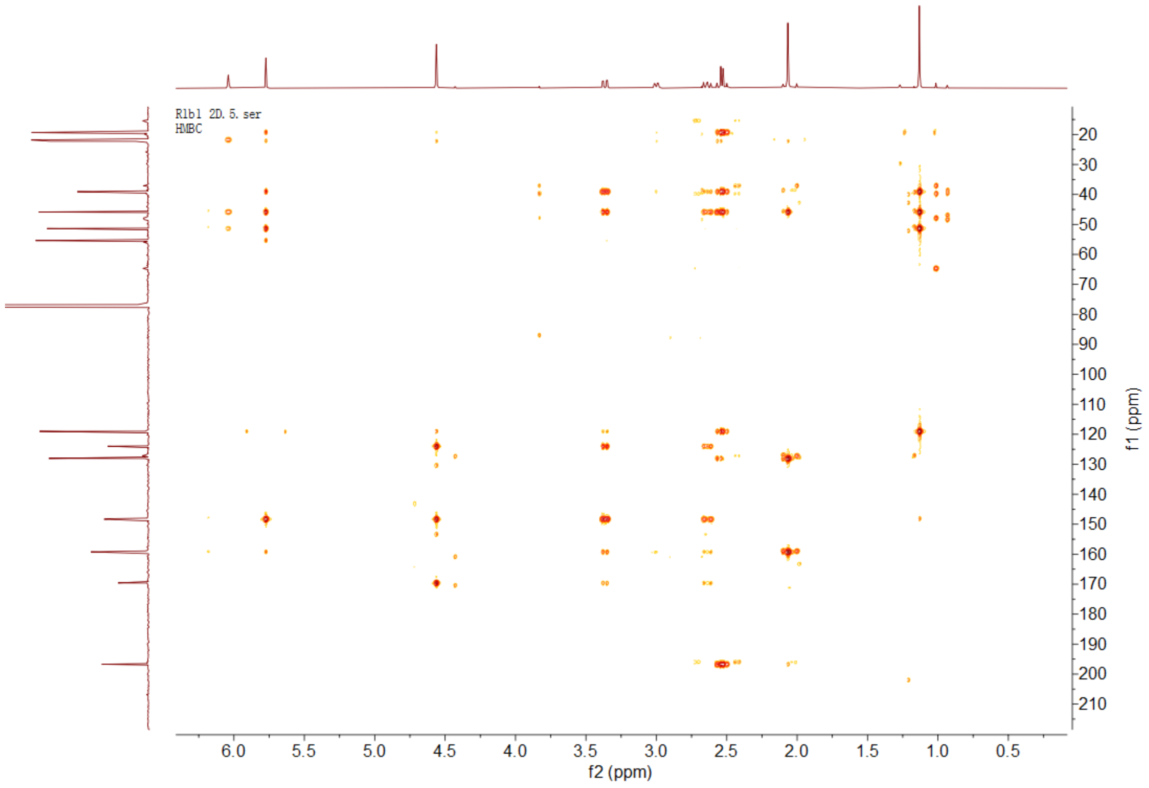

S76. NOESY spectrum of Eutypellaolide G (**7**) in $CDCl_3$

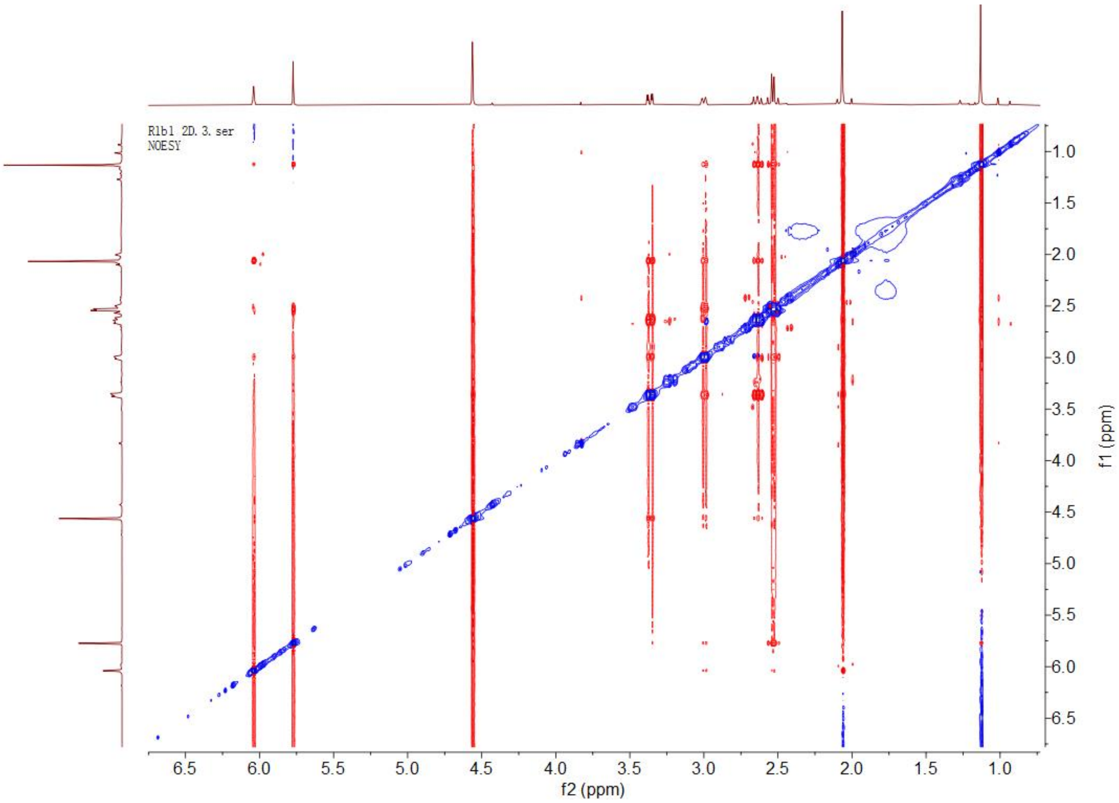

S77. HRESIMS of Eutypellaolide G (**7**)

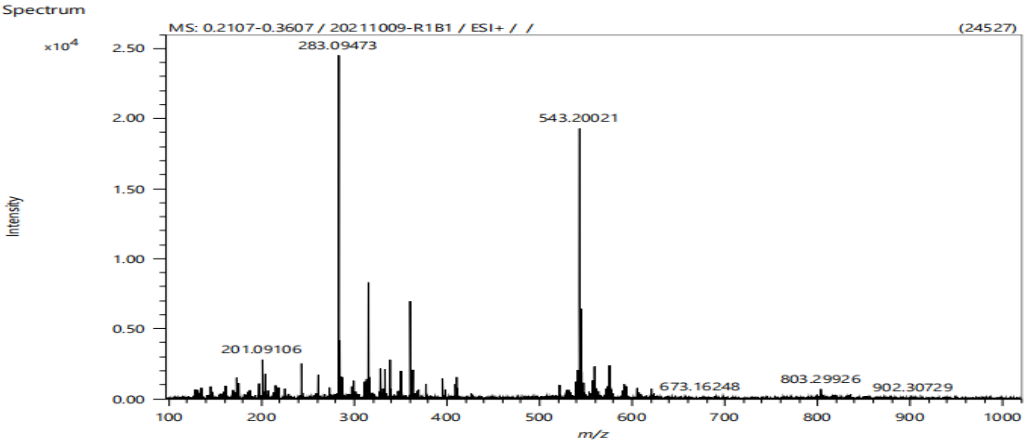

Elemental Composition

Parameters

Tolerance:	±5.00 ppm
Electron:	Odd/Even
Charge:	+1
DBE:	-1.5 - 200.0

Elements Set 1:

Symbol	C	H	N	O	Na	S	Cl	Br
Min	0	0	0	0	1	0	0	0
Max	200	200	0	8	1	0	0	0

Symbol	P	Si	F
Min	0	0	0
Max	0	0	0

Results

Mass	Intensity	Intensity [%]	Formula	Calculated Mass	Mass Difference [mDa]	Mass Difference [ppm]	DBE
283.09473	24526.59	100.00	C15 H16 O4 Na	283.09408	0.65	2.28	7.5

S78. UV spectrum of Eutypellaolide G (7) in MeOH

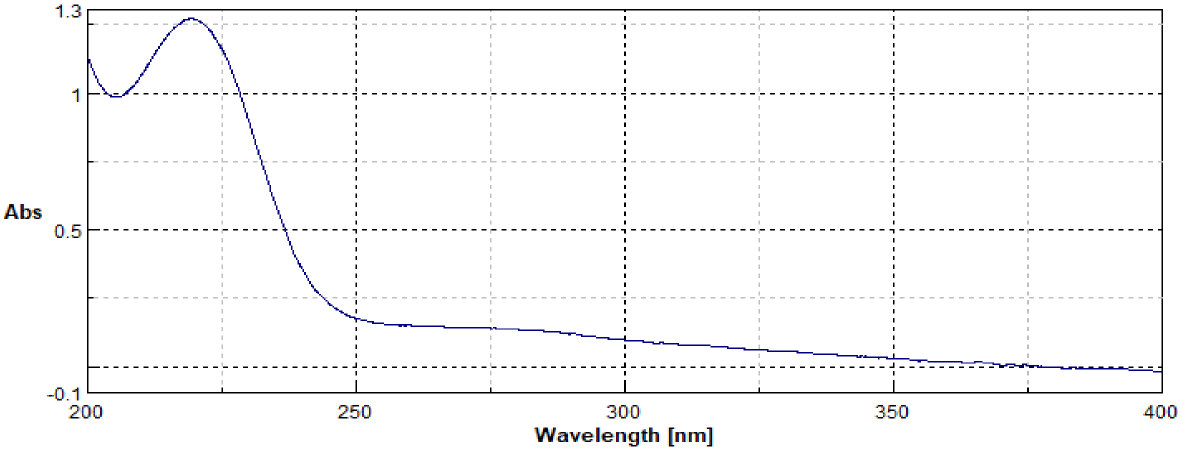

S79. IR spectrum of Eutypellaolide G (7)

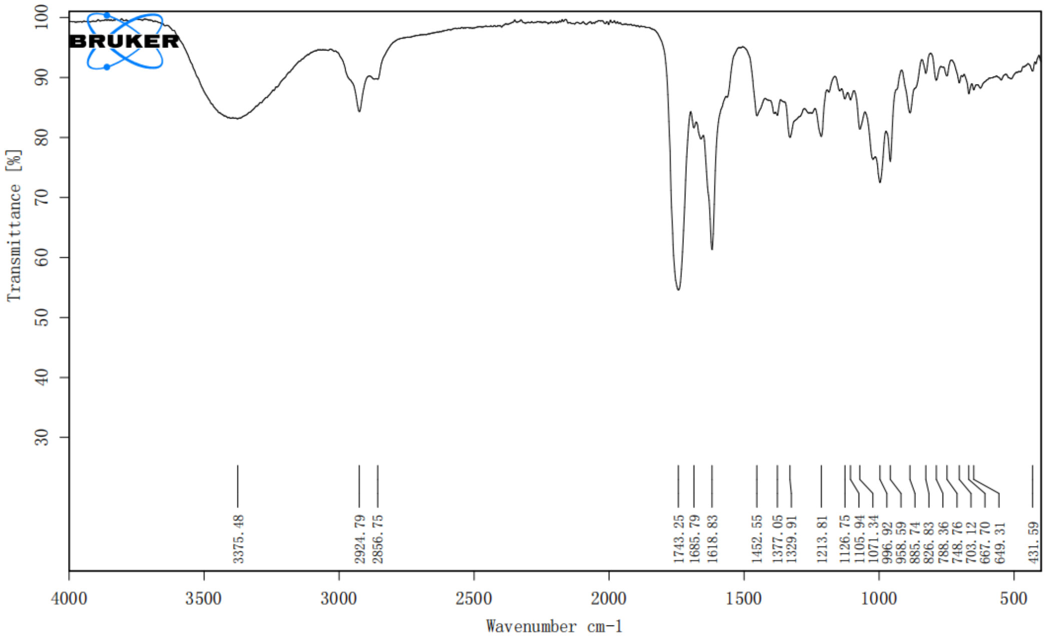

S80. ^{1}H NMR spectrum of Eutypellaolide H (**8**) in MeOH

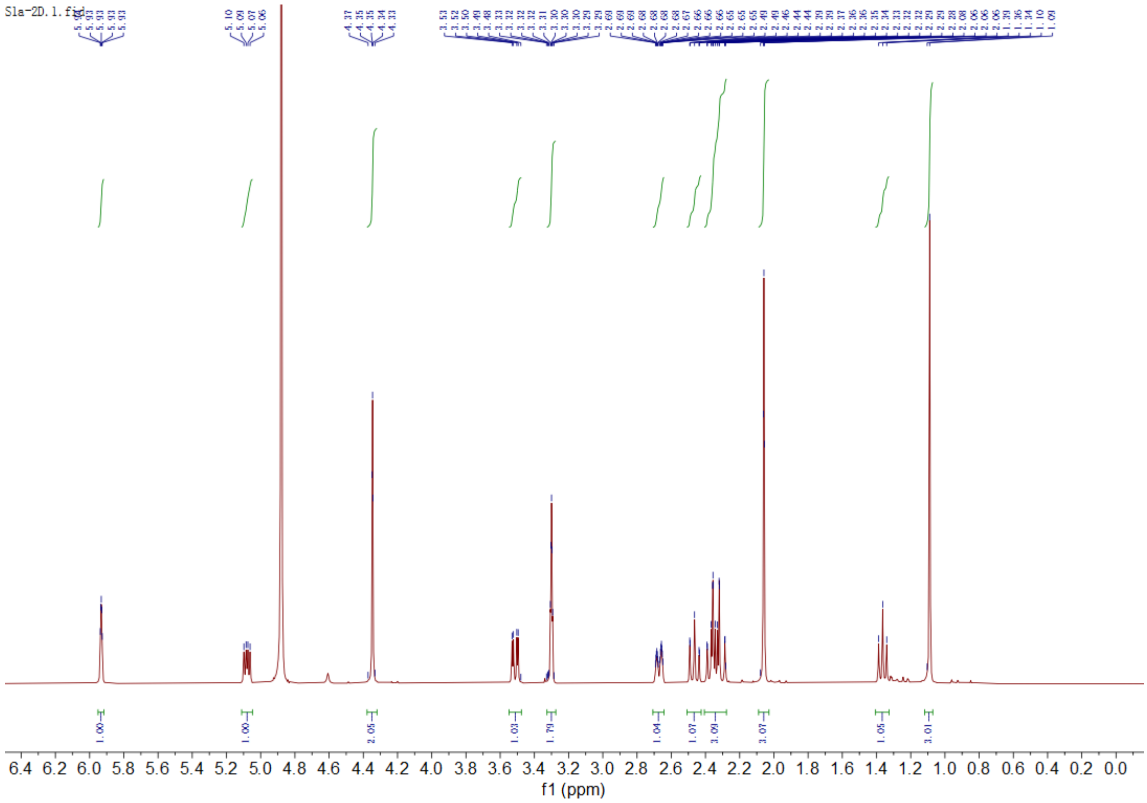

S81. ^{13}C NMR spectrum of Eutypellaolide H (**8**) in MeOH

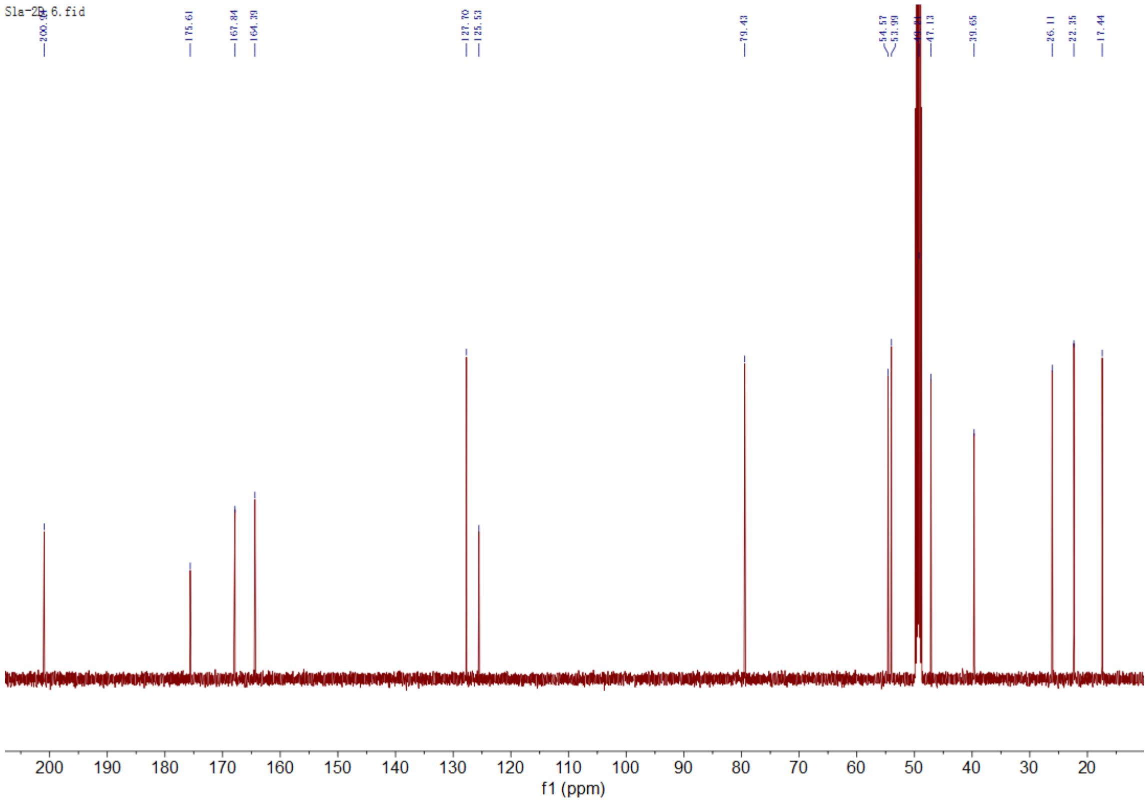

S82. DEPT135 spectrum of Eutypellaolide H (**8**) in MeOH

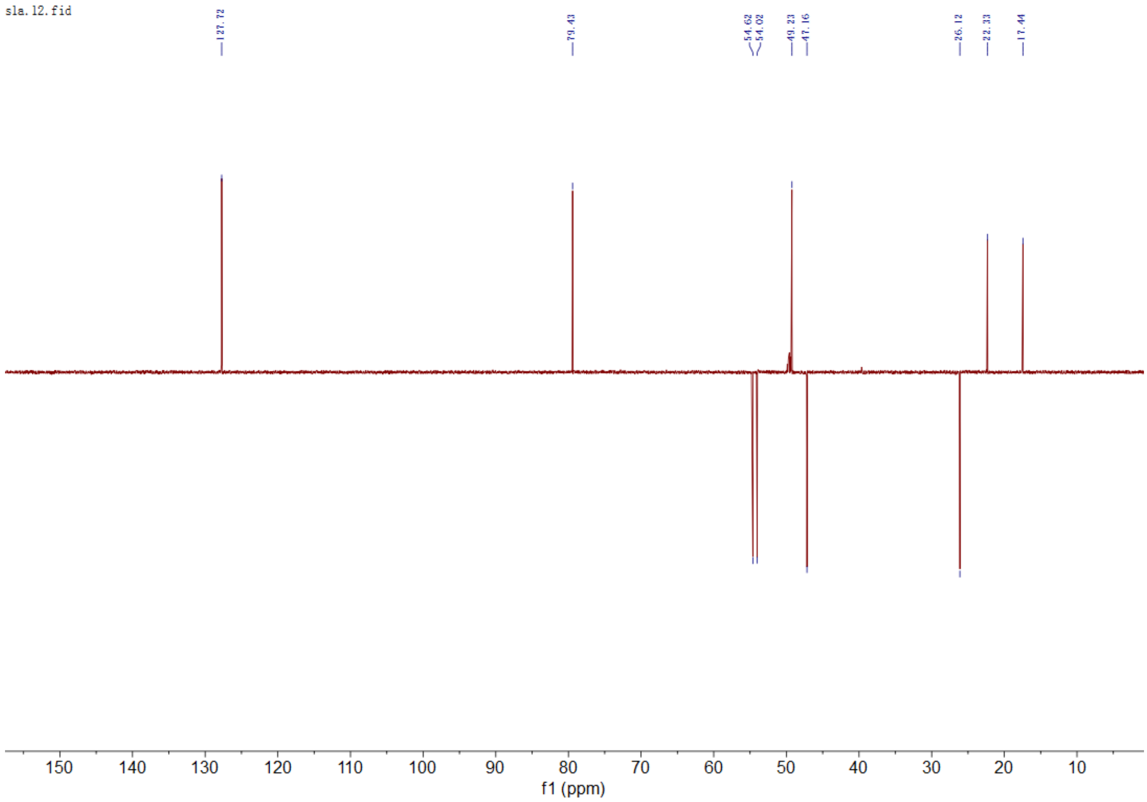

S83. HSQC spectrum of Eutypellaolide H (**8**) in MeOH

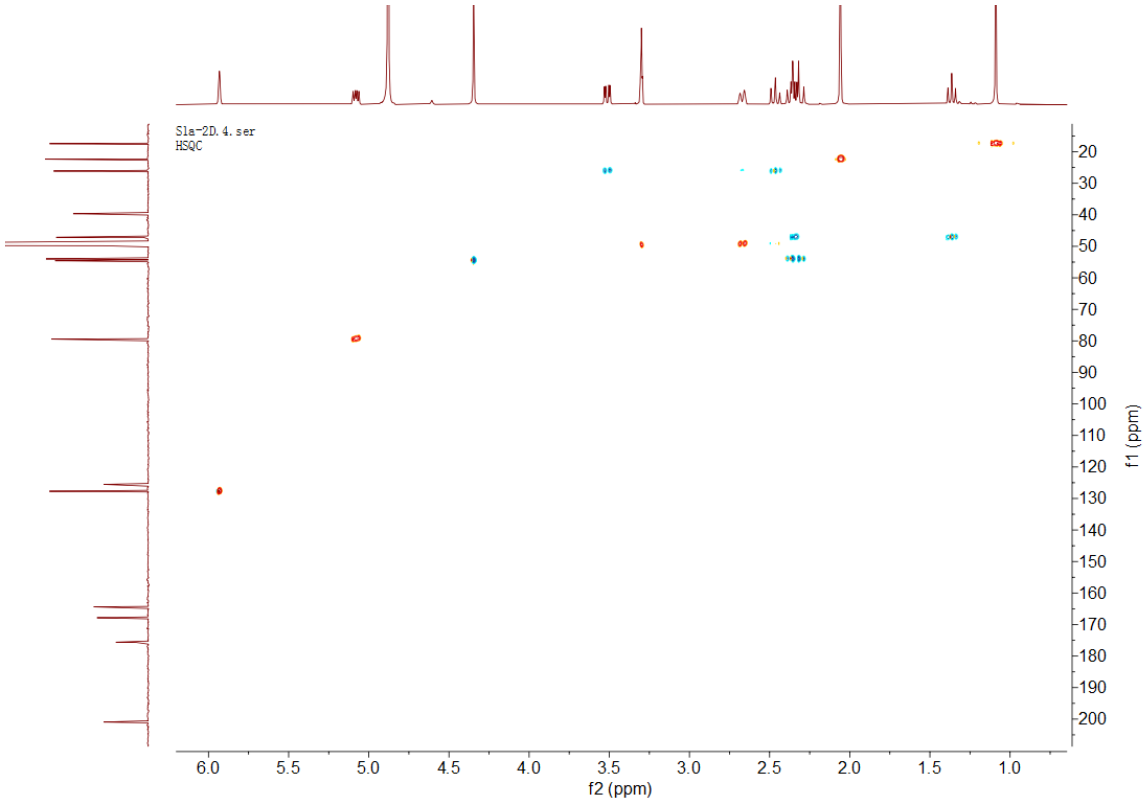

S84. COSY spectrum of Eutypellaolide H (**8**) in MeOH

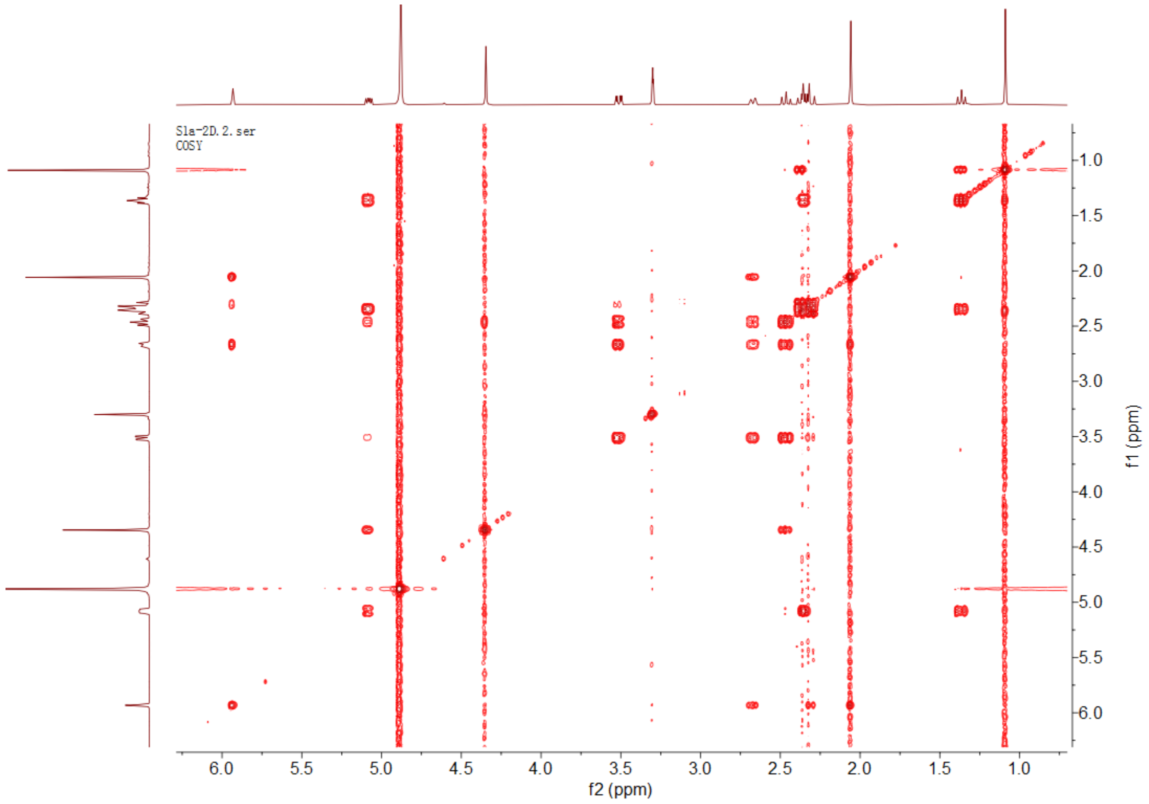

S85. HMBC spectrum of Eutypellaolide H (**8**) in MeOH

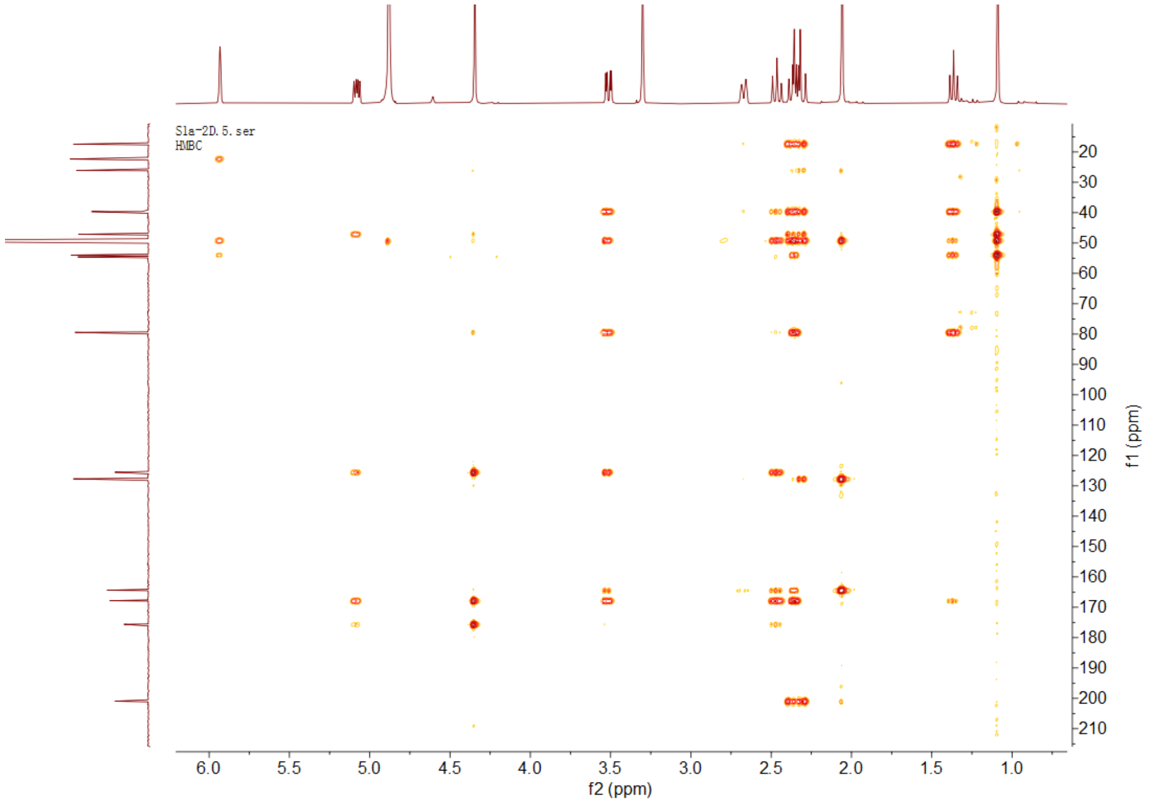

S86. NOESY spectrum of Eutypellaolide H (**8**) in MeOH

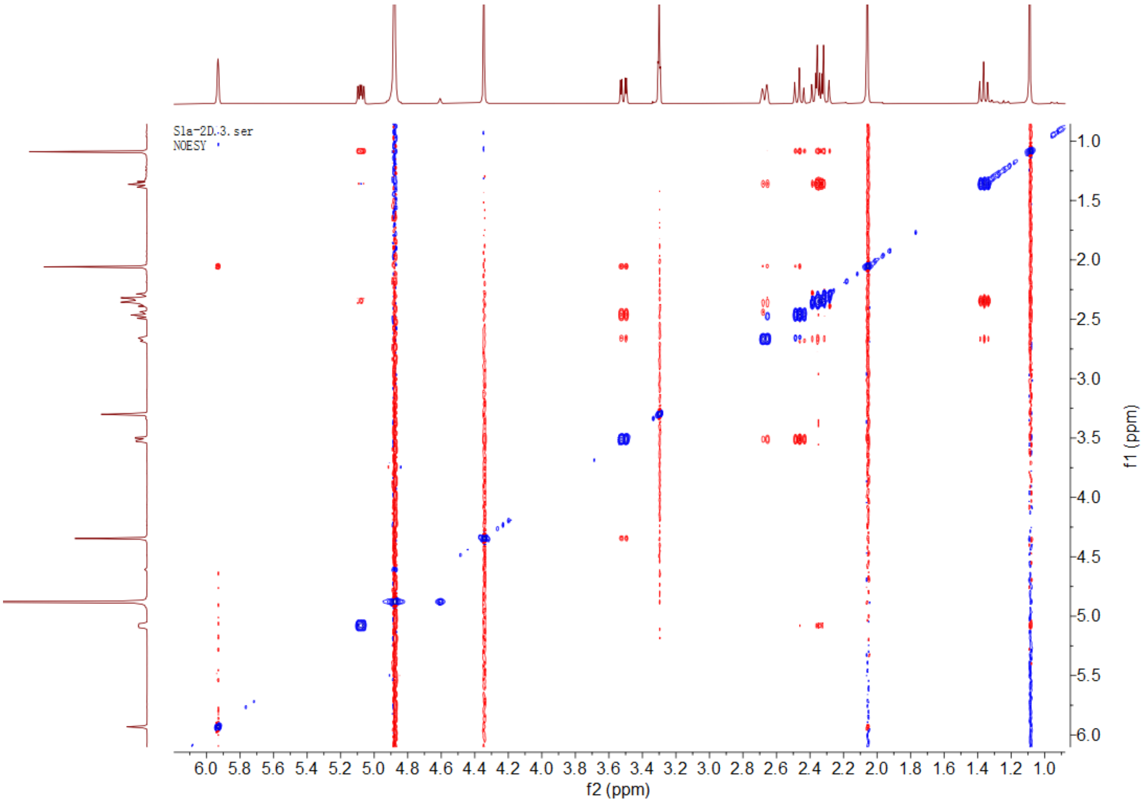

S87. HRESIMS of Eutypellaolide H (**8**)

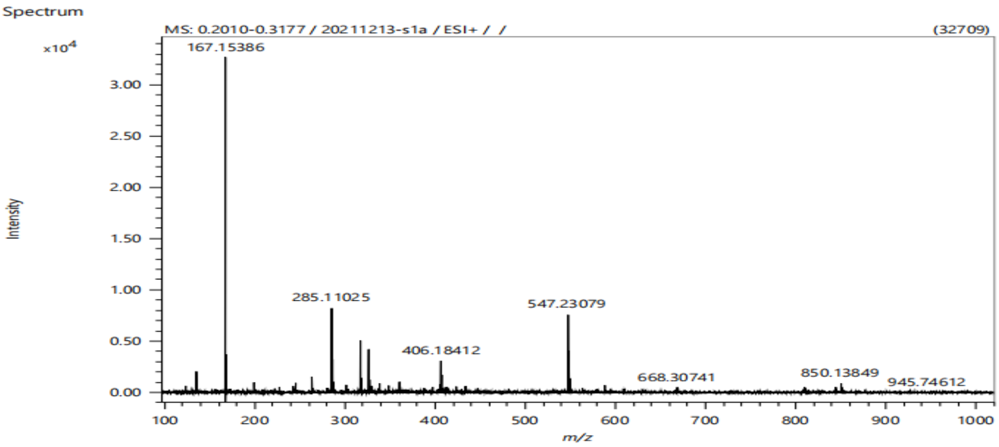

Elemental Composition

Parameters

Tolerance:	±5.00 ppm
Electron:	Odd/Even
Charge:	+1
DBE:	-1.5 - 200.0

Elements Set 1:

Symbol	C	H	N	O	Na	S	Cl	Br
Min	0	0	0	0	1	0	0	0
Max	200	200	3	8	1	0	0	0

Symbol	P	F
Min	0	0
Max	0	0

Results

Mass	Intensity	Intensity [%]	Formula	Calculated Mass	Mass Difference [mDa]	Mass Difference [ppm]	DBE
285.11025	821143	25.10	C15 H18 O4 Na	285.10973	0.52	1.84	6.5

S88. UV spectrum of Eutypellaolide H (**8**) in MeOH

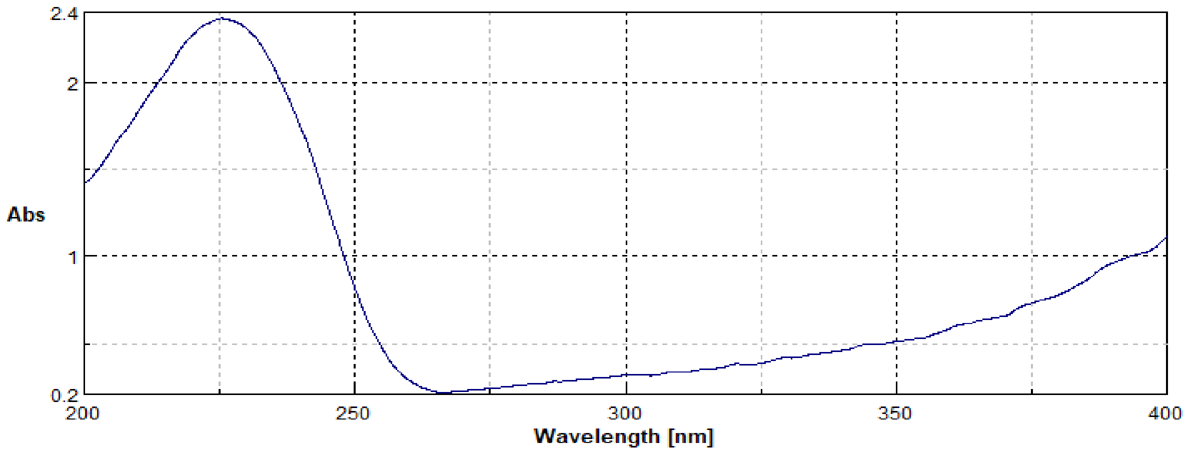

S89. IR spectrum of Eutypellaolide H (**8**)

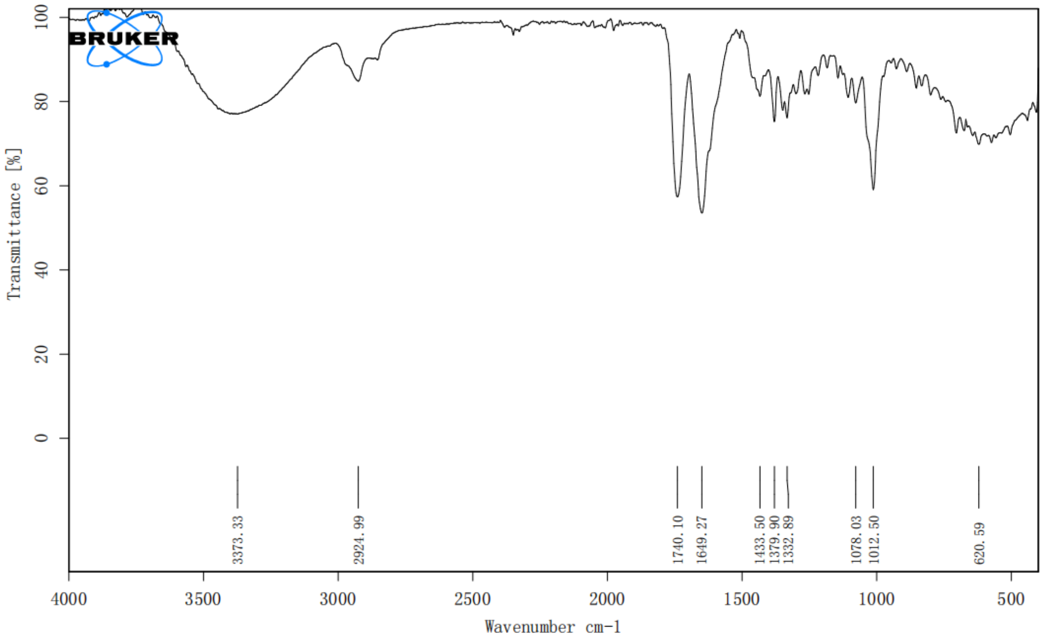

S90. ^{1}H NMR spectrum of Eutypellaolide I (**9**) in DMSO-*d6*

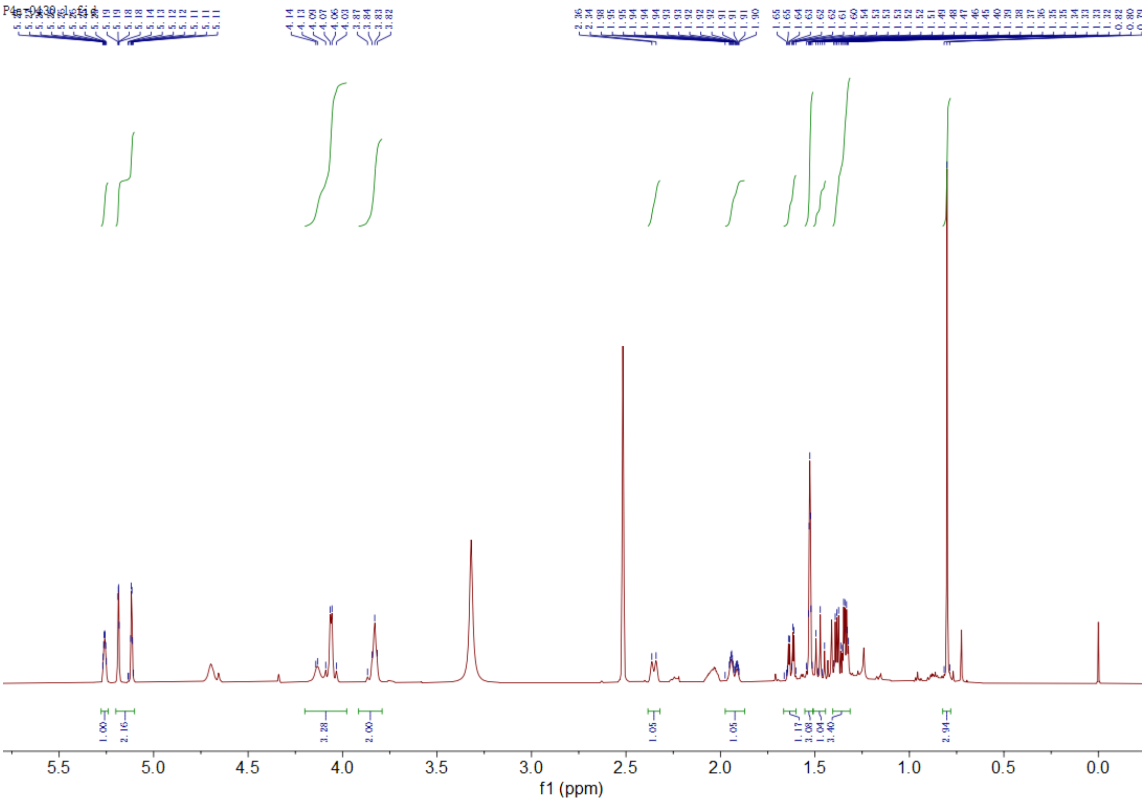

S91. ^{13}C NMR spectrum of Eutypellaolide I (**9**) in DMSO-*d6*

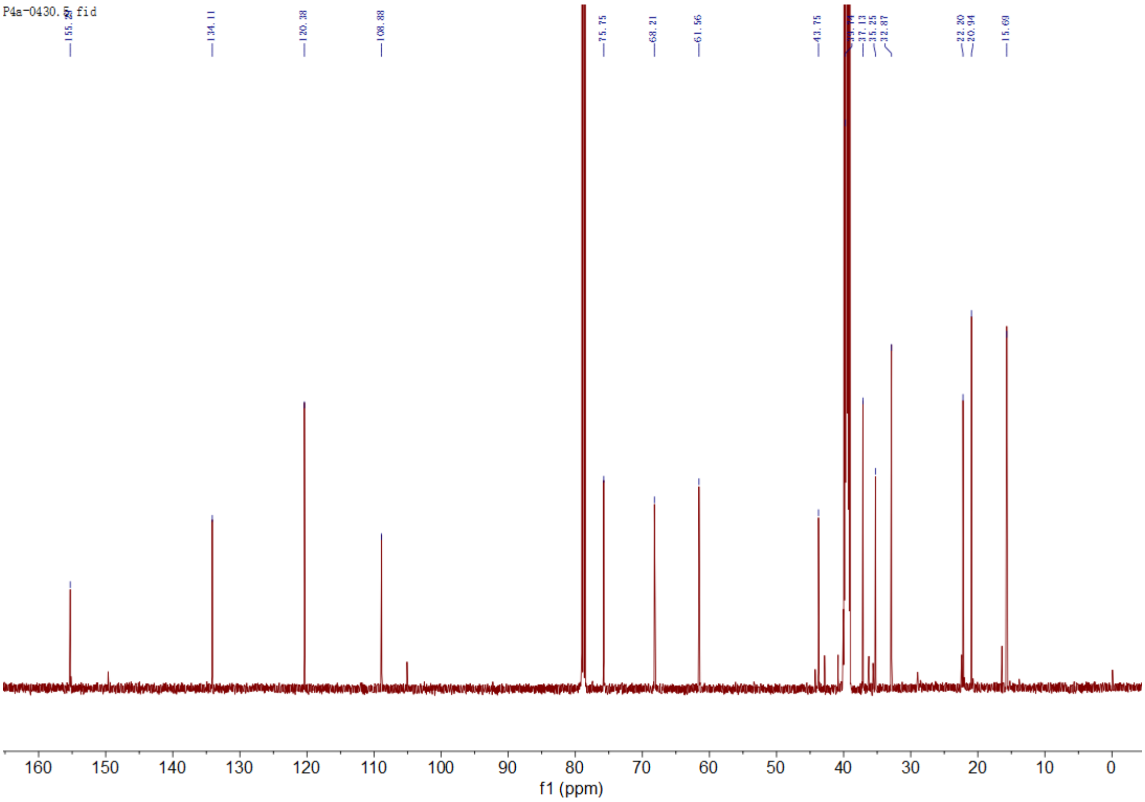

S92. DEPT135 spectrum of Eutypellaolide I (**9**) in DMSO-*d6*

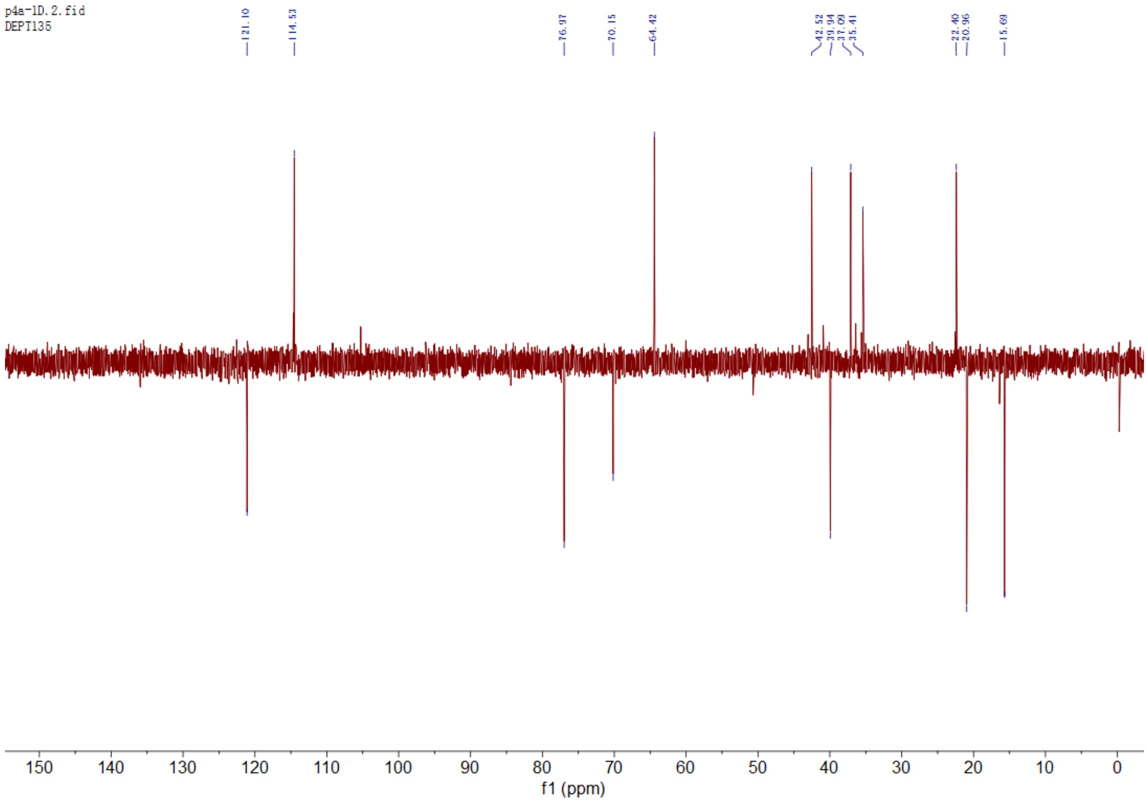

S93. HSQC spectrum of Eutypellaolide I (**9**) in DMSO-*d6*

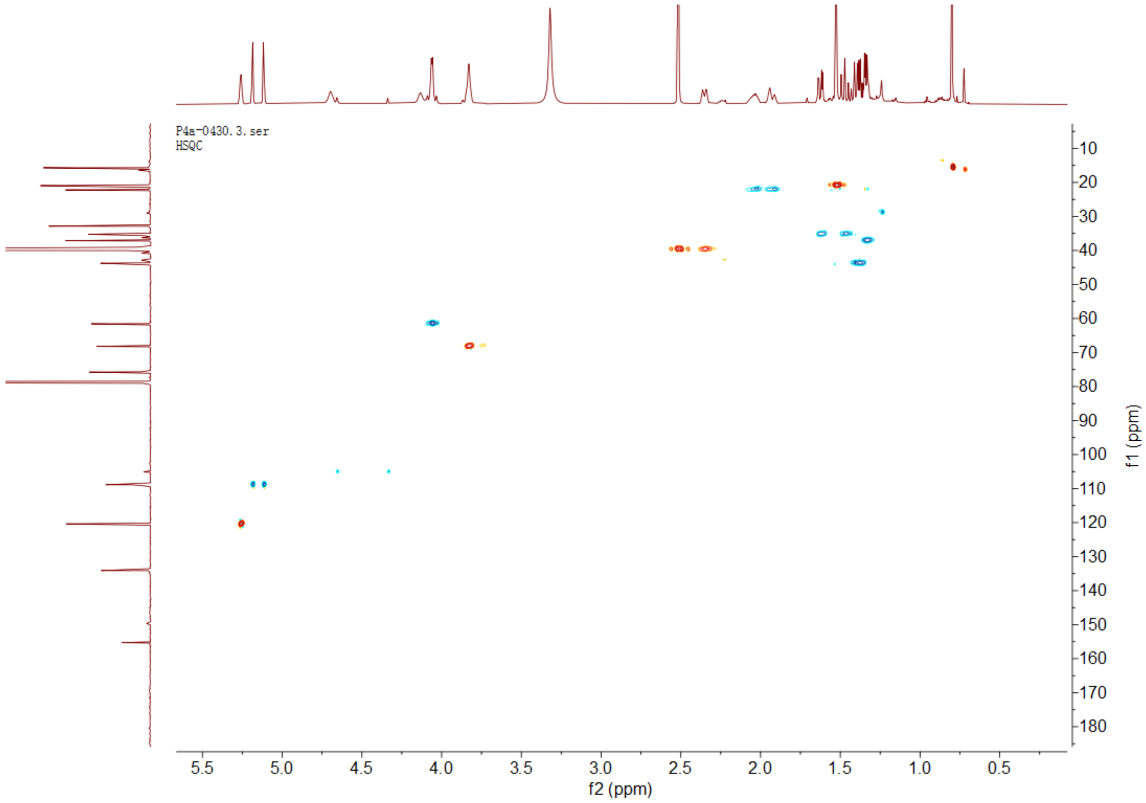

S94. COSY spectrum of Eutypellaolide I (**9**) in DMSO-*d6*

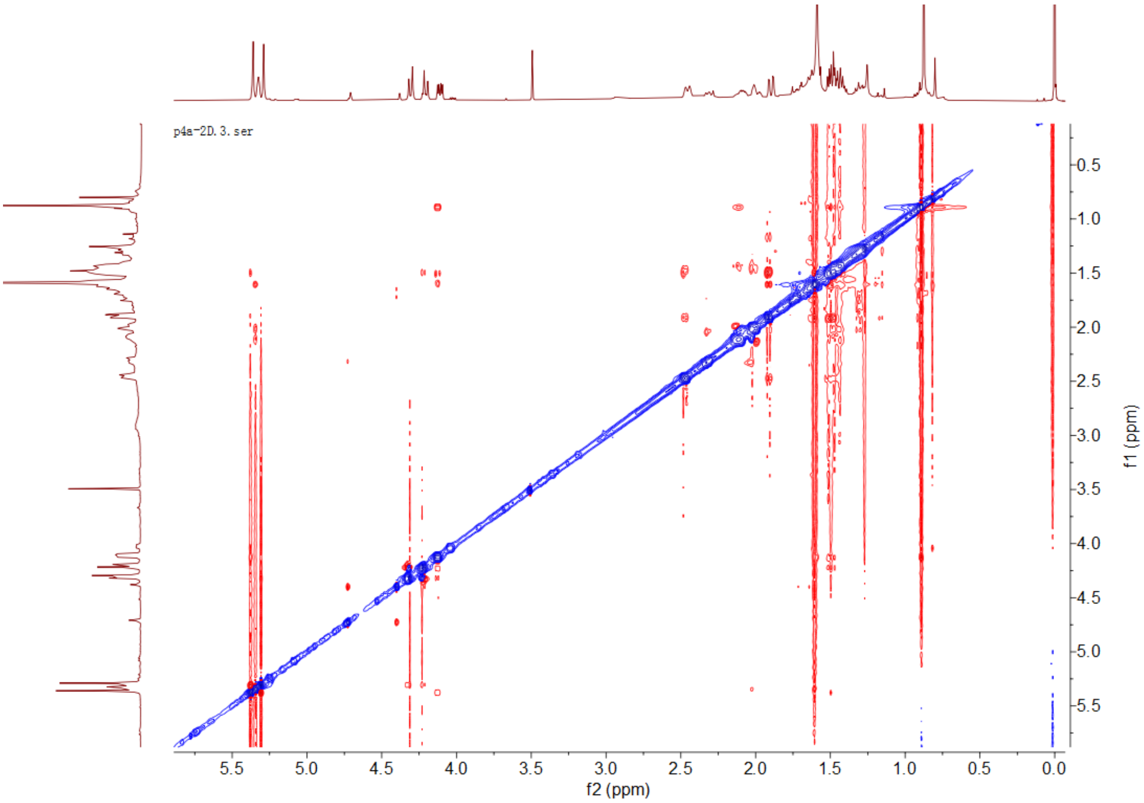

S95. HMBC spectrum of Eutypellaolide I (**9**) in DMSO-*d6*

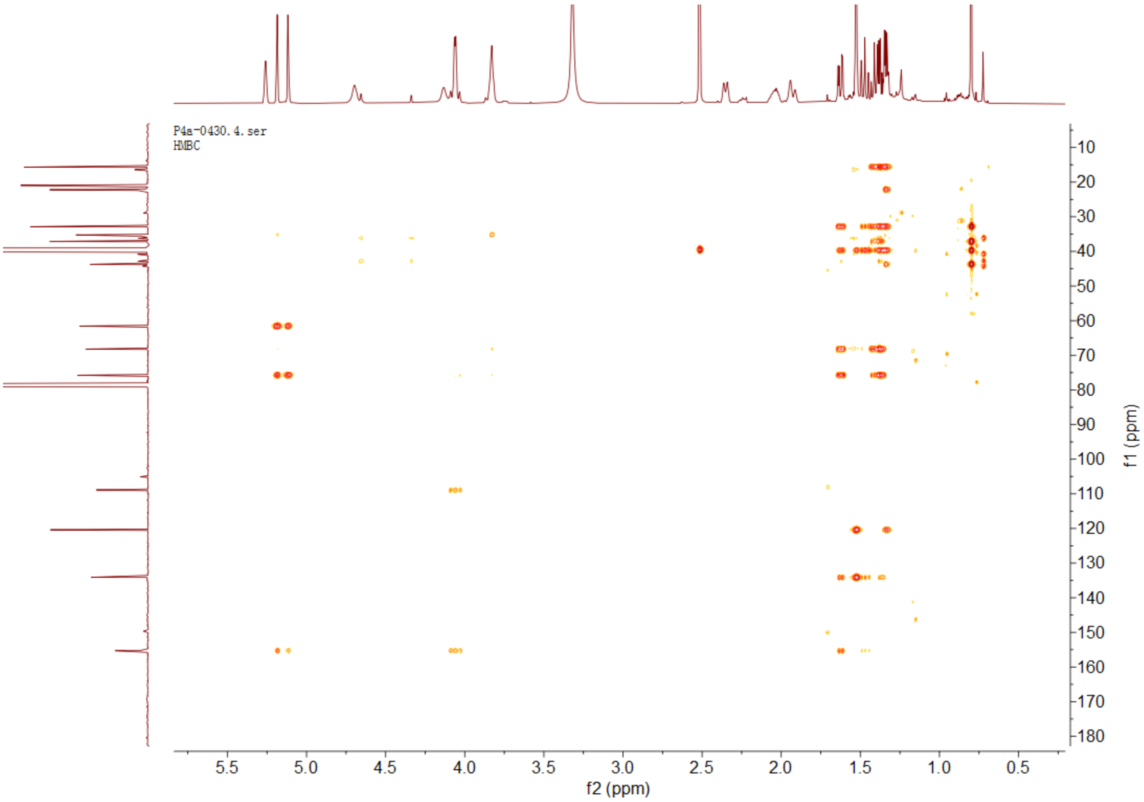

S96. NOESY spectrum of Eutypellaolide I (**9**) in DMSO-*d6*

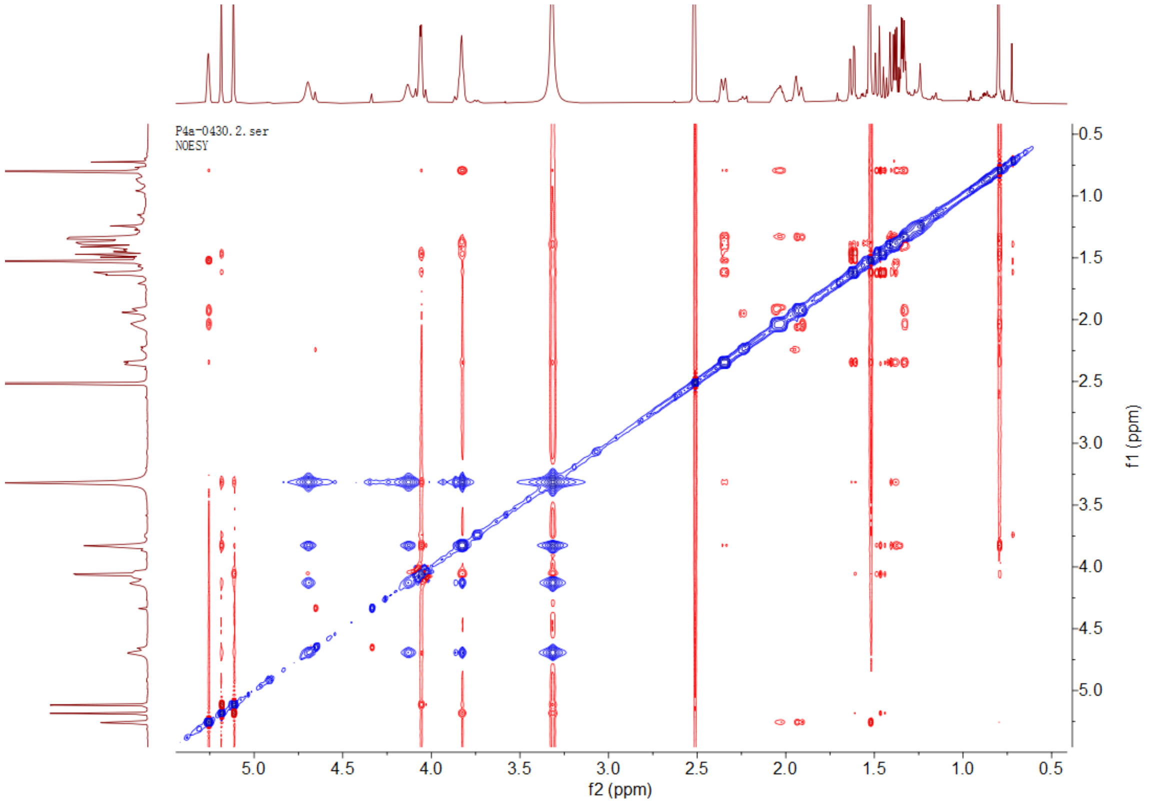

S97. HRESIMS of Eutypellaolide I (**9**)

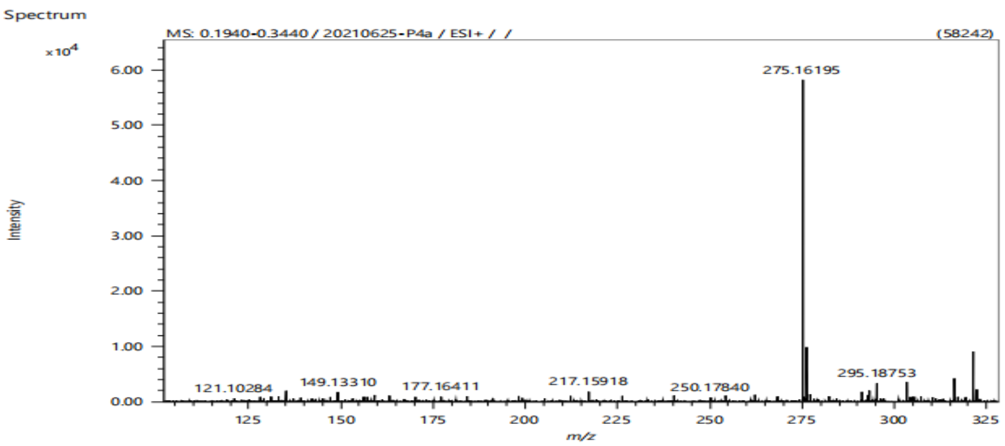

Elemental Composition

Parameters

Tolerance:	±5.00 ppm
Electron:	Odd/Even
Charge:	+1
DBE:	-1.5 - 200.0

Elements Set 1:

Symbol	C	H	N	O	Na	S	Cl	Br
Min	0	0	0	0	1	0	0	0
Max	200	200	3	8	1	0	0	0

Symbol	F	Si
Min	0	0
Max	0	0

Results

Mass	Intensity	Intensity [%]	Formula	Calculated Mass	Mass Difference [mDa]	Mass Difference [ppm]	DBE
275.16195	58242.15	74.05	C15 H24 O3 Na	275.16177	0.18	0.66	3.5

S98. UV spectrum of Eutypellaolide I (**9**) in MeOH

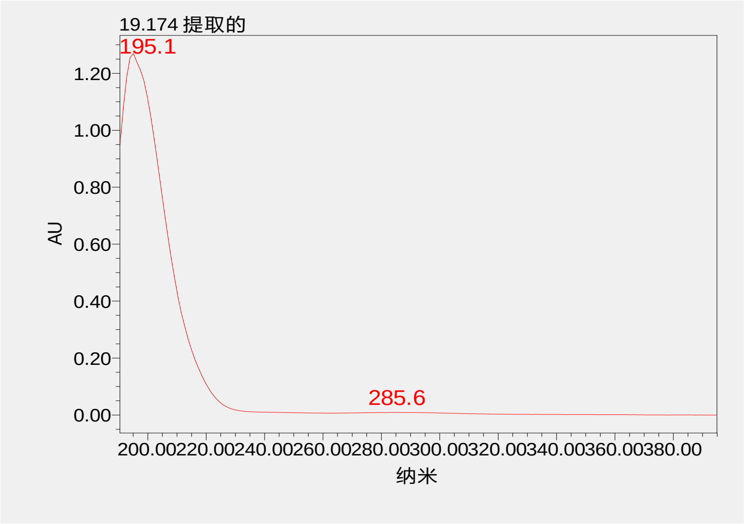

S99. IR spectrum of Eutypellaolide I (**9**)

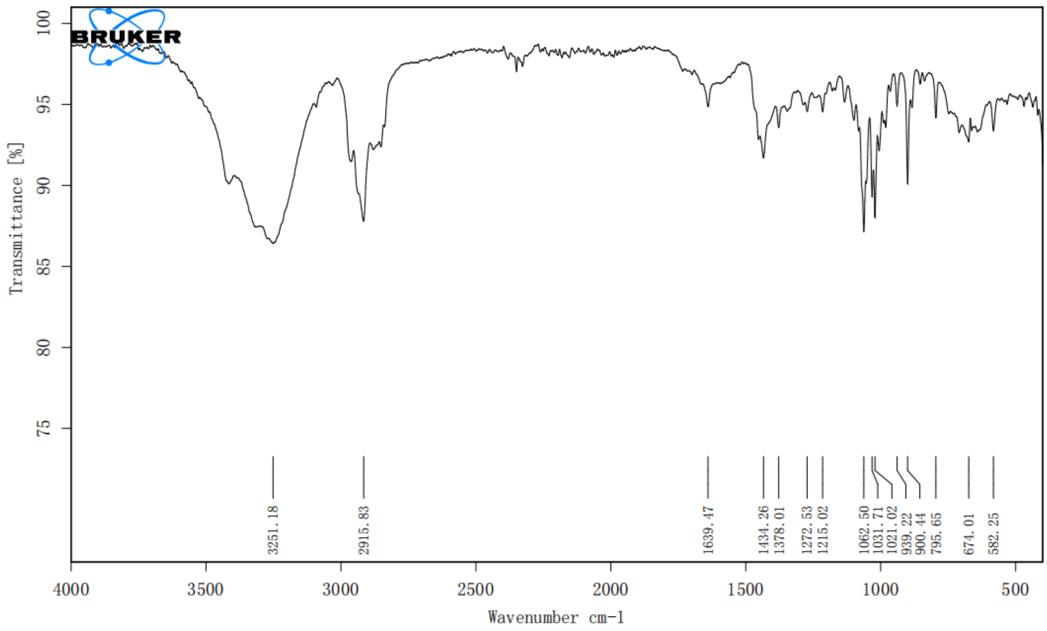

S100. ^{1}H NMR spectrum of Eutypellaolide J (**10**) in MeOH

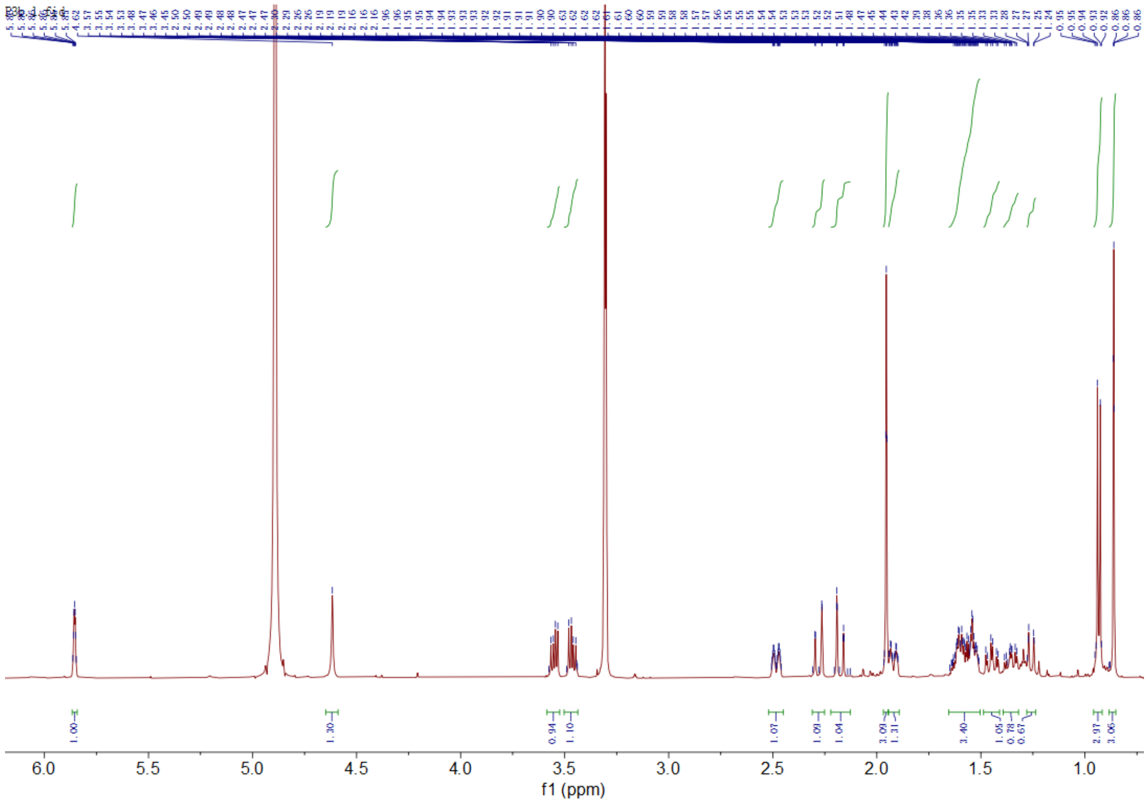

S101. ^{13}C NMR spectrum of Eutypellaolide J (**10**) in MeOH

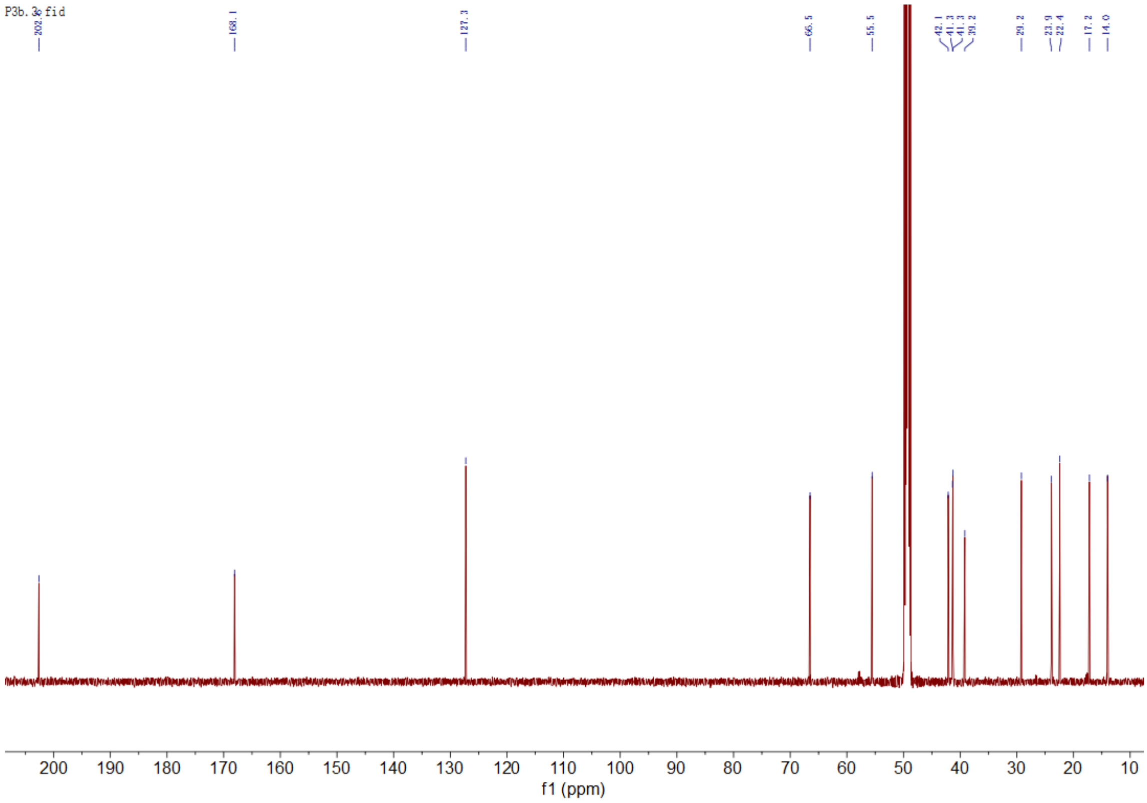

S102. DEPT135 spectrum of Eutypellaolide J (**10**) in MeOH

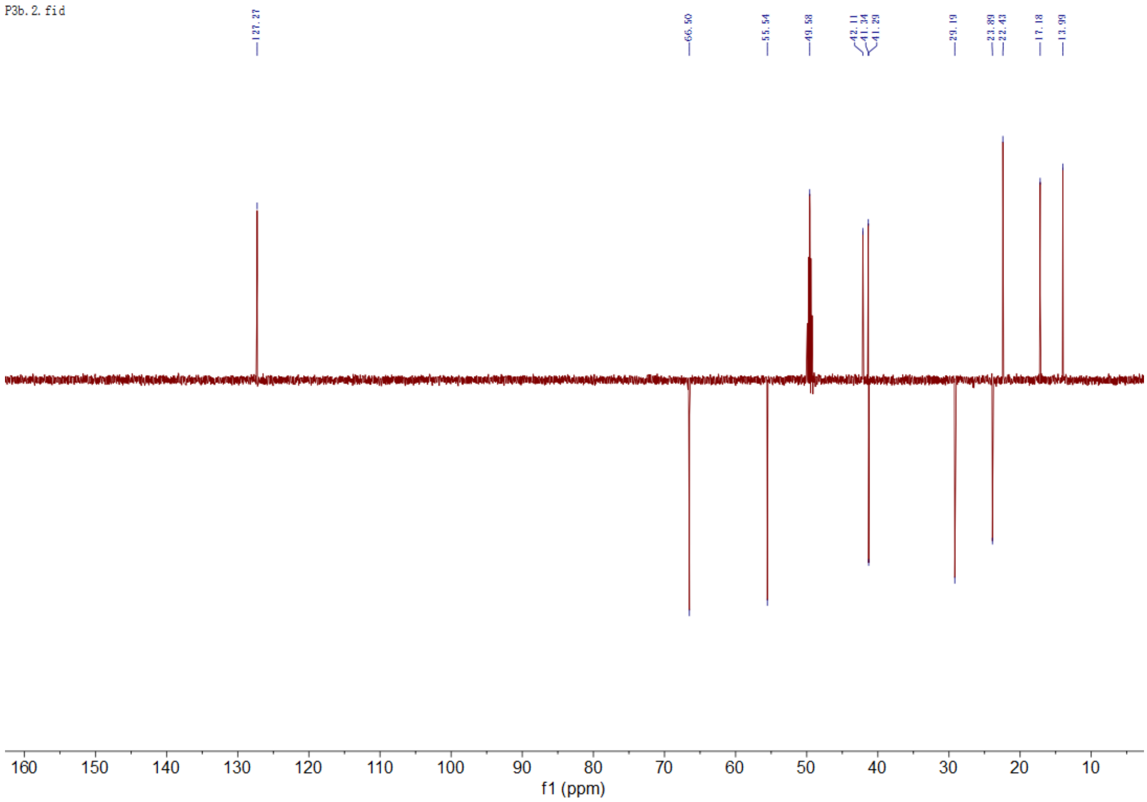

S103. HSQC spectrum of Eutypellaolide J (**10**) in MeOH

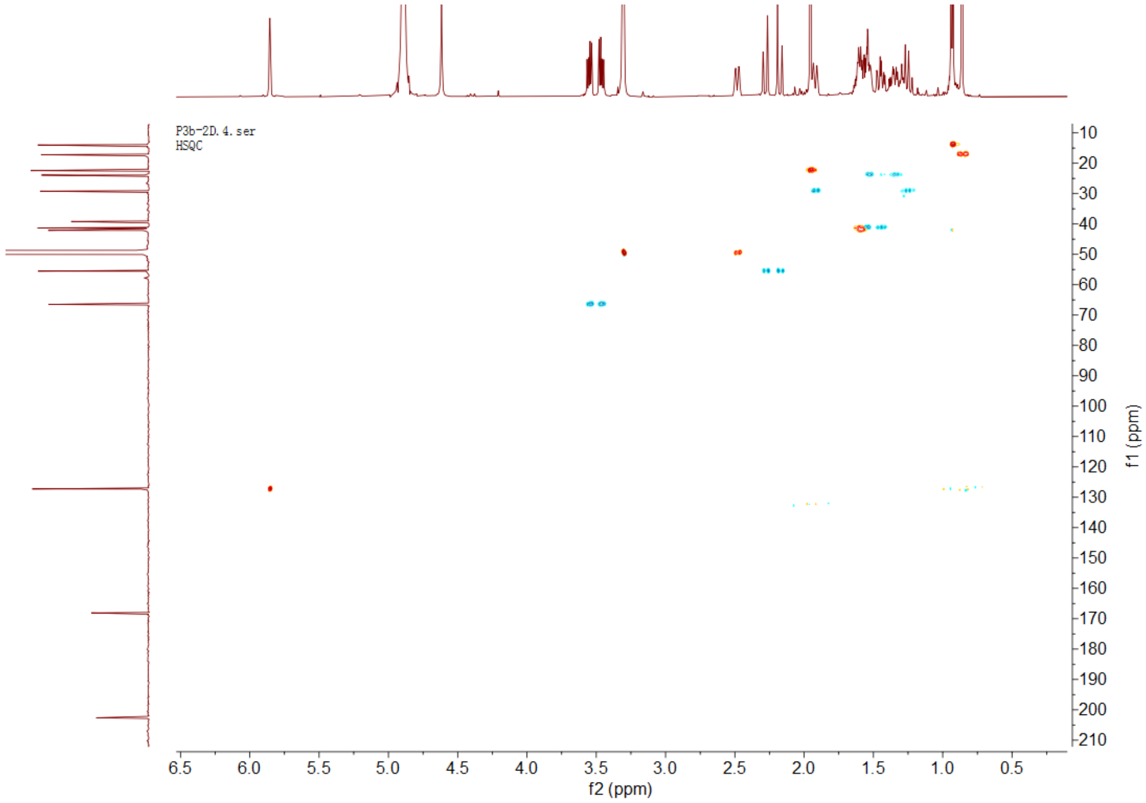

S104. COSY spectrum of Eutypellaolide J (**10**) in MeOH

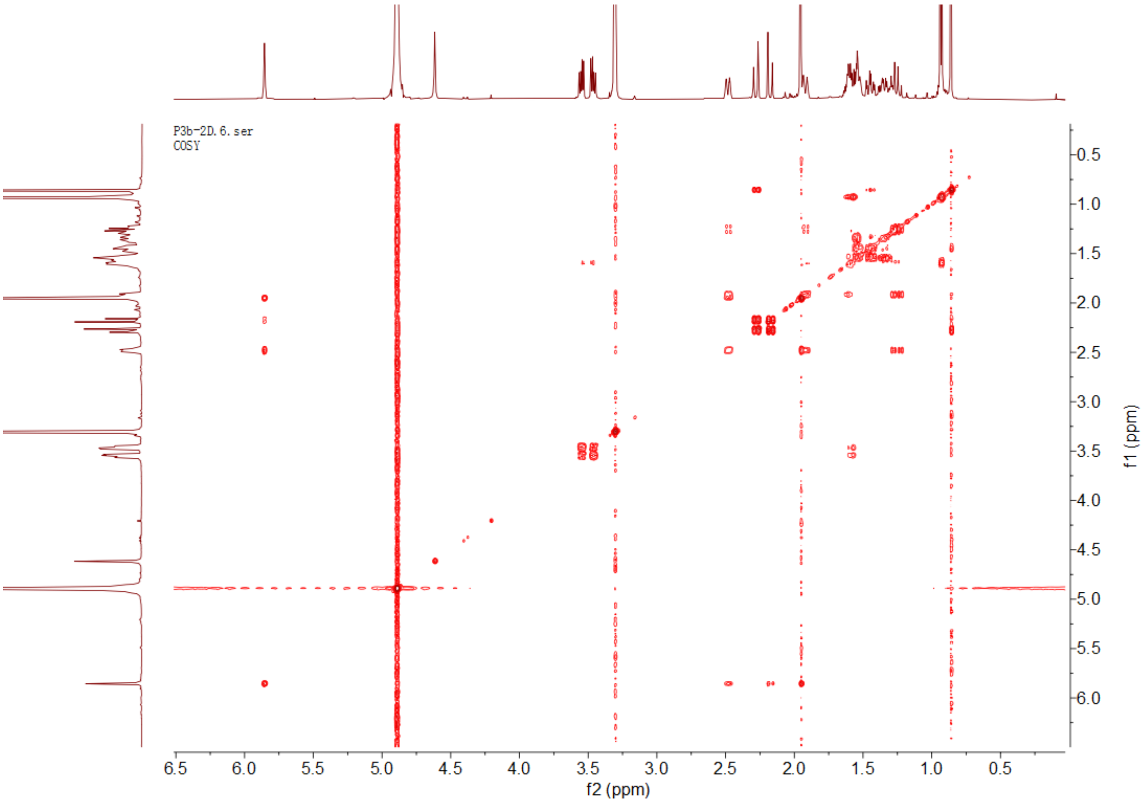

S105. HMBC spectrum of Eutypellaolide J (**10**) in MeOH

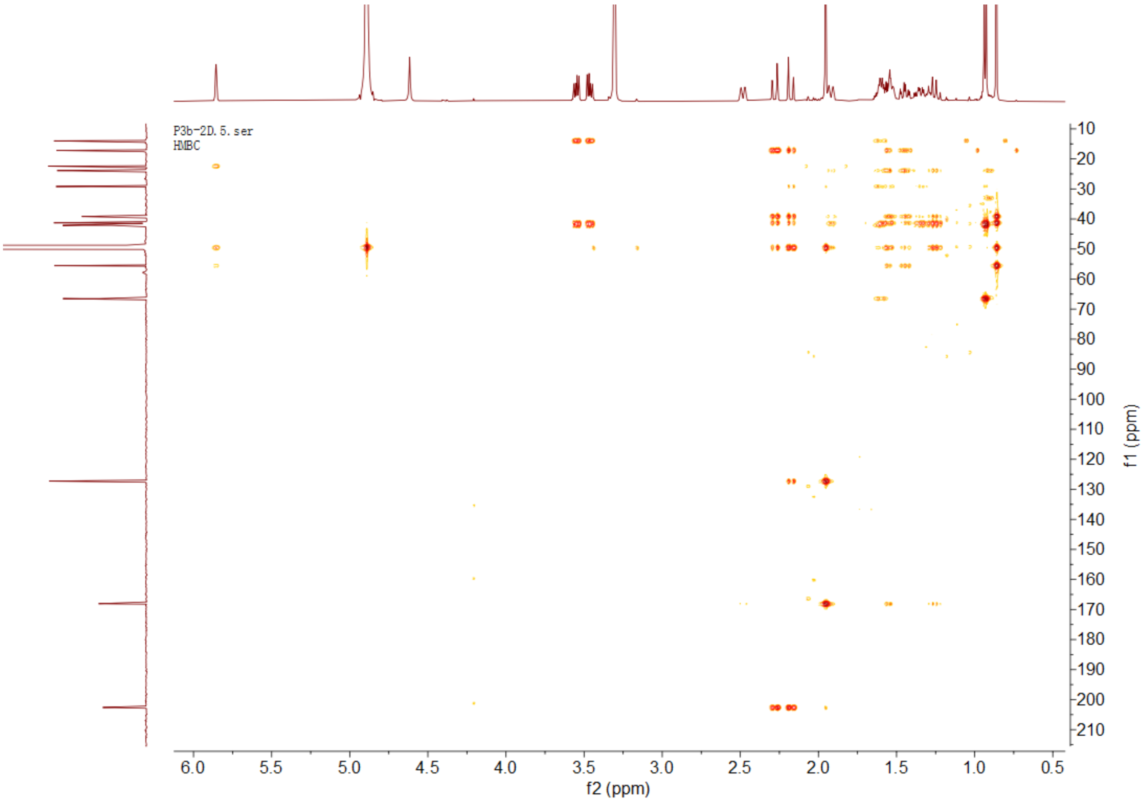

S106. NOESY spectrum of Eutypellaolide J (**10**) in MeOH

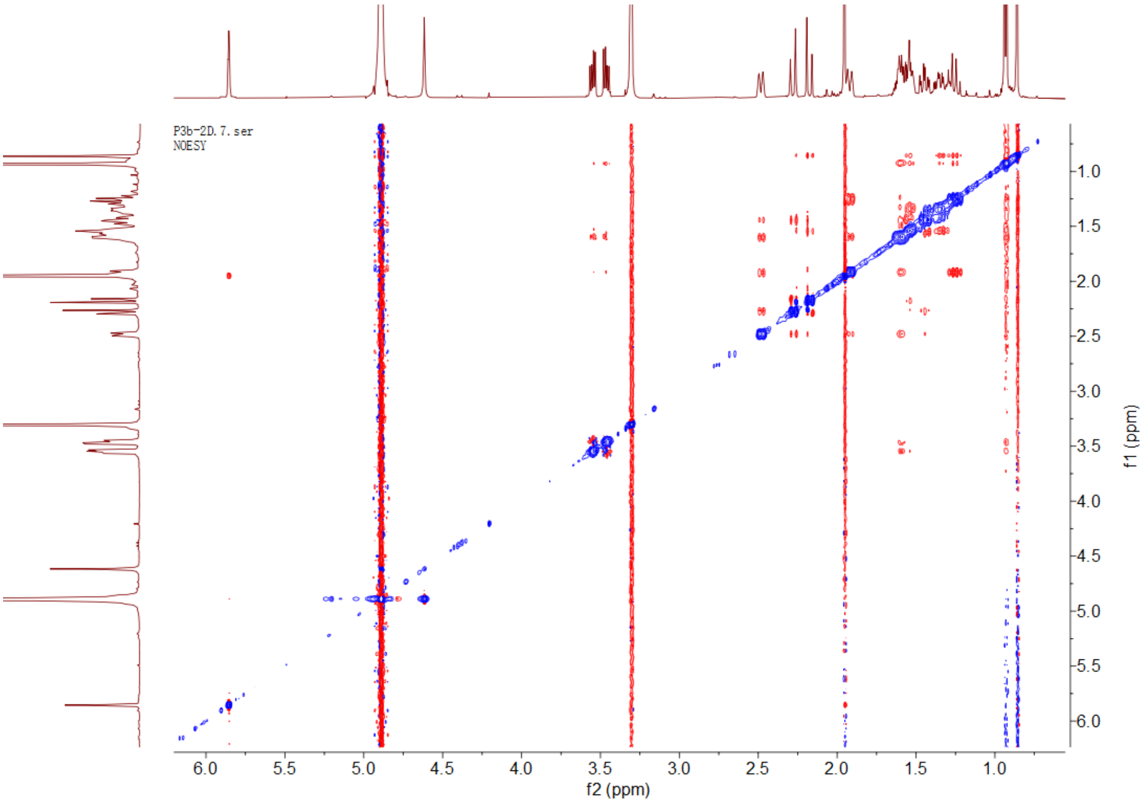

S107. HRESIMS of Eutypellaolide J (**10**)

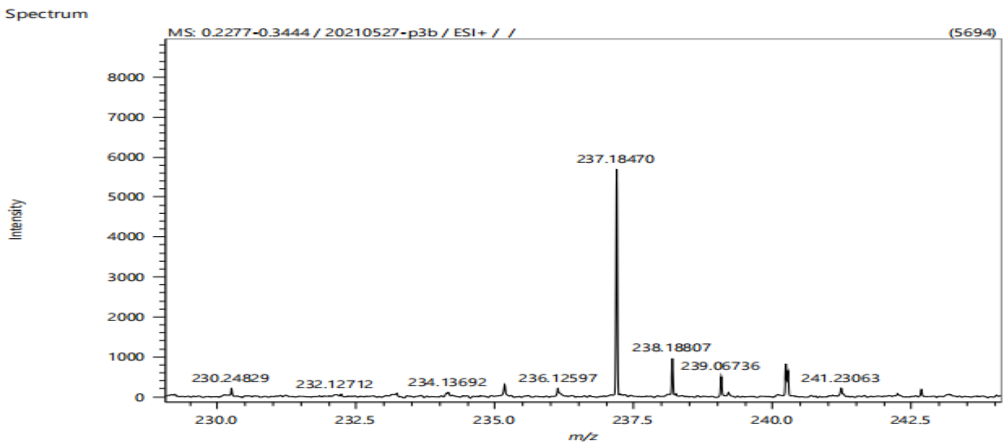

Elemental Composition

Parameters	
Tolerance:	±5.00 ppm
Electron:	Odd/Even
Charge:	+1
DBE:	-1.5 - 200.0

Elements Set 1:

Symbol	C	H	N	O	Na	S	Cl	Br
Min	0	0	0	0	0	0	0	0
Max	200	200	0	8	0	0	0	0

Symbol	F	Si
Min	0	0
Max	0	0

Results

Mass	Intensity	Intensity [%]	Formula	Calculated Mass	Mass Difference [mDa]	Mass Difference [ppm]	DBE
237.18470	5693.65	9.35	C15 H25 O2	237.18491	-0.21	-0.87	3.5

S108. UV spectrum of Eutypellaolide J (**10**) in MeOH

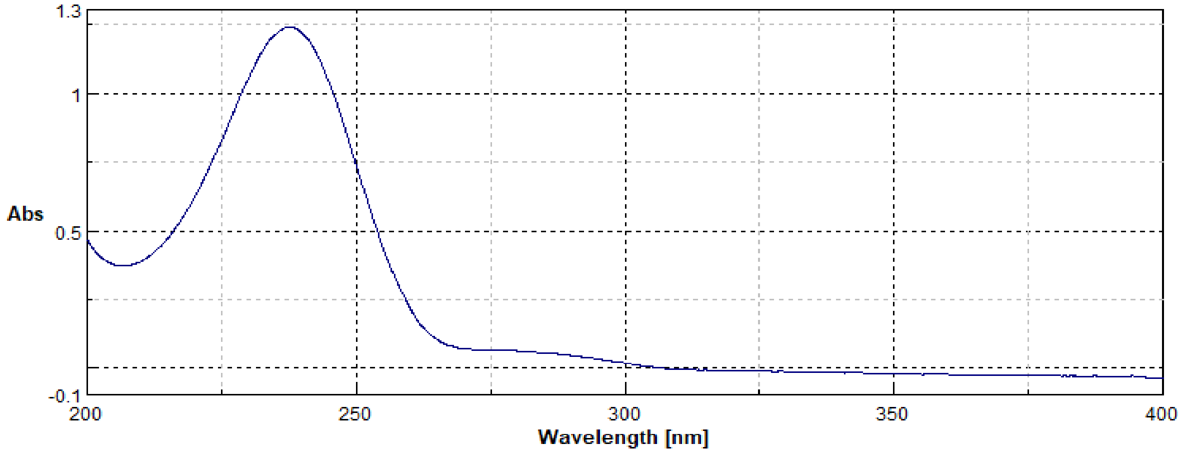

S109. IR spectrum of Eutypellaolide J (**10**)

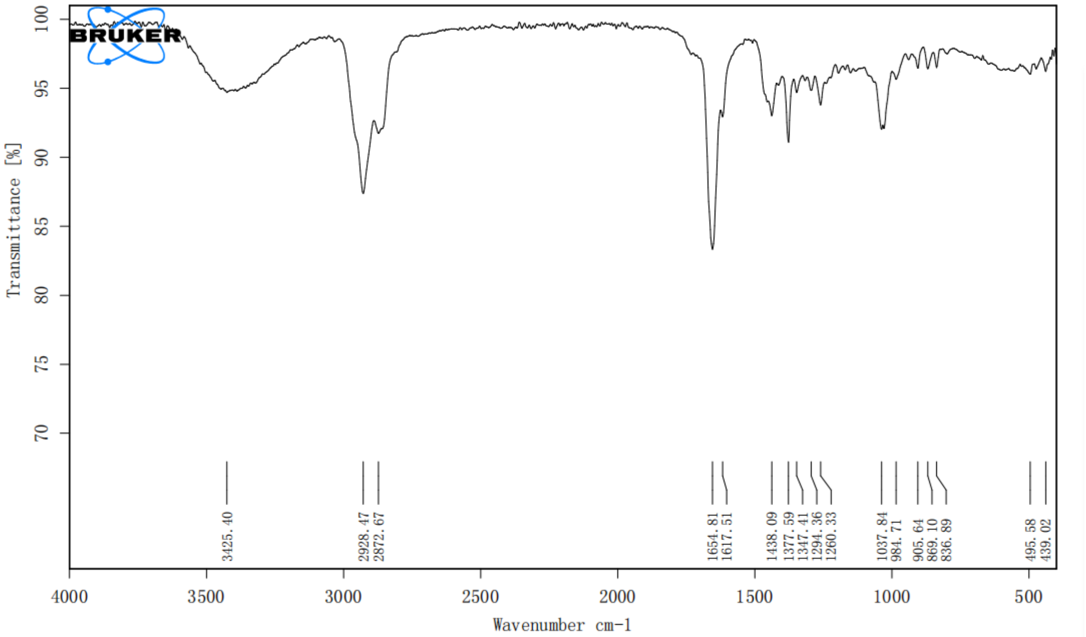

S110. The HPLC analysis of the extract at different culture conditions compared with standard substances (a: PDB medium; b: solid defined medium; c: compound **11**).

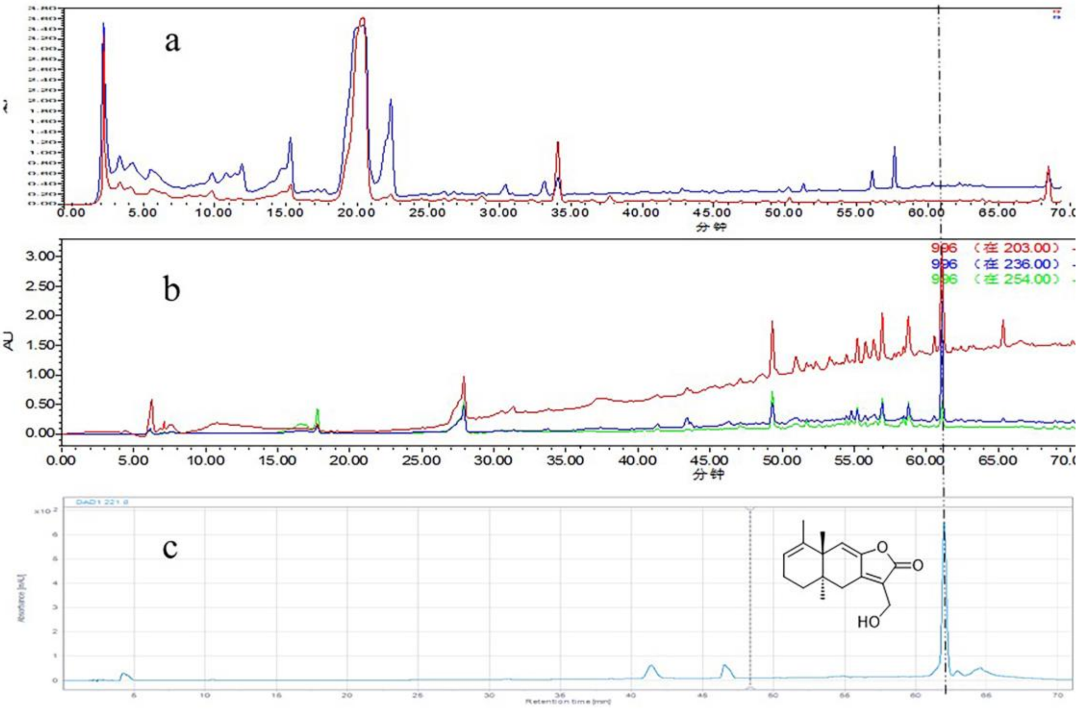
a
b
c
AU
分钟
996 （在 203.00）
996 （在 236.00）
996 （在 254.00）
HO
O
O
